# Sirt1 inhibits the recruitment of RNA polymerase II to metabolic gene promoter during pressure overload through competition with the preinitiation complex on Sub1

**DOI:** 10.1093/cvr/cvag178

**Published:** 2026-08-21

**Authors:** Shin-ichi Oka, Youssef Mourad, Samta Veera, Peiyong Zhai, Ralph Alcendor, Yunping Guo, Jamie Ades, Sanchita Kashyap, Xiaoyong Xu, Chengchen Hu, Toshihide Kashihara, Takanobu Yamamoto, Yoshiyuki Ikeda, Akihiro Shirakabe, Shohei Ikeda, Wataru Mizushima, Yasuki Nakada, Danish Sayed, Zhi Yang, Maha Abdellatif, Junichi Sadoshima

**Affiliations:** Department of Cell Biology and Molecular Medicine, Rutgers New Jersey Medical School, Newark, NJ 07103, USA; Department of Cell Biology and Molecular Medicine, Rutgers New Jersey Medical School, Newark, NJ 07103, USA; Department of Cell Biology and Molecular Medicine, Rutgers New Jersey Medical School, Newark, NJ 07103, USA; Department of Cell Biology and Molecular Medicine, Rutgers New Jersey Medical School, Newark, NJ 07103, USA; Department of Cell Biology and Molecular Medicine, Rutgers New Jersey Medical School, Newark, NJ 07103, USA; Department of Cell Biology and Molecular Medicine, Rutgers New Jersey Medical School, Newark, NJ 07103, USA; Department of Cell Biology and Molecular Medicine, Rutgers New Jersey Medical School, Newark, NJ 07103, USA; Department of Cell Biology and Molecular Medicine, Rutgers New Jersey Medical School, Newark, NJ 07103, USA; Department of Cell Biology and Molecular Medicine, Rutgers New Jersey Medical School, Newark, NJ 07103, USA; Department of Cell Biology and Molecular Medicine, Rutgers New Jersey Medical School, Newark, NJ 07103, USA; Department of Cell Biology and Molecular Medicine, Rutgers New Jersey Medical School, Newark, NJ 07103, USA; Department of Cell Biology and Molecular Medicine, Rutgers New Jersey Medical School, Newark, NJ 07103, USA; Department of Cell Biology and Molecular Medicine, Rutgers New Jersey Medical School, Newark, NJ 07103, USA; Department of Cell Biology and Molecular Medicine, Rutgers New Jersey Medical School, Newark, NJ 07103, USA; Department of Cell Biology and Molecular Medicine, Rutgers New Jersey Medical School, Newark, NJ 07103, USA; Department of Cell Biology and Molecular Medicine, Rutgers New Jersey Medical School, Newark, NJ 07103, USA; Department of Cell Biology and Molecular Medicine, Rutgers New Jersey Medical School, Newark, NJ 07103, USA; Department of Cell Biology and Molecular Medicine, Rutgers New Jersey Medical School, Newark, NJ 07103, USA; Department of Cell Biology and Molecular Medicine, Rutgers New Jersey Medical School, Newark, NJ 07103, USA; Department of Cell Biology and Molecular Medicine, Rutgers New Jersey Medical School, Newark, NJ 07103, USA; Department of Cell Biology and Molecular Medicine, Rutgers New Jersey Medical School, Newark, NJ 07103, USA

**Keywords:** Sirt1, Sub1, Pol II, Preinitiation complex, Metabolic remodelling

## Abstract

**Aims:**

Heart failure is marked by suppression of fatty acid oxidation (FAO) and mitochondrial ATP production gene expression. While transcriptional downregulation via PGC-1 and PPARα/ERRα has been well documented, the involvement of the general transcriptional machinery remains insufficiently understood. Distinct from its salutary role in many cardiac conditions, endogenous Sirt1 negatively affects cardiac function during pressure overload (PO). This study investigates how Sirt1 modulates preinitiation complex (PIC) assembly and RNA polymerase II (Pol II) recruitment during pathological PO.

**Methods and results:**

Cardiac-specific Sirt1 knock-out (Sirt1 cKO) mice were subjected to PO. Interaction between Sirt1 and Sub1, a protein recruiting GTFs to the gene promoter, was assessed with co-immunoprecipitation, protein pull-down, and molecular docking. Pol II recruitment was evaluated with chromatin immunoprecipitation-sequencing (ChIP-seq) and ChIP-qPCR analyses. The binding affinity of Sirt1 and GTF to the Sub1 GTF binding domain was assessed by immunoprecipitation and protein pull-down assays. Unbiased ChIP-seq and ChIP-qPCR analyses showed that Pol II binding to metabolic gene promoters was downregulated during PO, which was reversed in Sirt1 cKO mice. PO upregulated Sirt1 in the heart and increased its binding to Sub1, which interacts with PPARα and ERRα but not NF-kB. Sirt1 binding to Sub1 competitively displaced the interaction between Sub1 and GTFs, thereby inhibiting PIC formation at the Sub1-PPARα/ERRα complex.

**Conclusion:**

Sirt1 impairs metabolic gene transcription during PO by competitively inhibiting Sub1-mediated recruitment of GTFs and PIC formation. This repression of general transcriptional machinery contributes to the metabolic disturbance and may represent a maladaptive component of the heart failure phenotype.

## Graphical Abstract

**Graphical Abstract:**
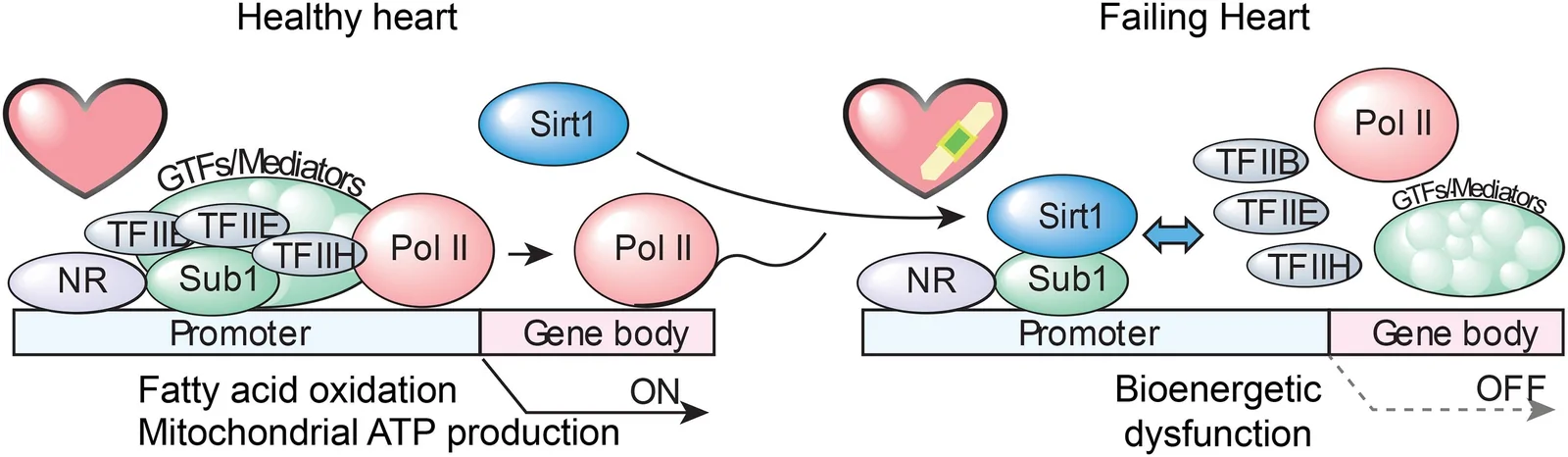

**Time of primary review: 47 days**

**See the editorial comment for this article ‘The unforeseen twist of Sirt1 in cardiac hypertrophy’, by T. Bozoglu**  ***et al*****., https://doi.org/10.1093/cvr/cvag218.**

## 1. Introduction

Metabolic disturbance coupled with the downregulation of metabolic genes in cardiomyocytes is a hallmark of the failing heart.^1,2^ Alteration of specific transcription factors (STFs) and epigenetic regulators acting on specific regulatory elements have been identified as a key mechanism driving transcriptional changes in heart failure. Past studies focused intensively on the role of STFs or transcription factor co-factor, such as PPAR, ERR, and PGC-1α in metabolic disturbance, with downregulation of the STFs or co-factors proposed as a responsible mechanism.^3^ However, normalization of PGC-1α expression has been shown to have only limited therapeutic efficacy,^4^ and cardiac-specific PPARα overexpression is actually detrimental in the failing heart.^5^

Besides STFs, the general transcriptional machinery termed preinitiation complex (PIC), consisting of general transcription factors (GTFs), such as TFIIB, TFIID, TFIIE, TFIIF, and TFIIH, RNA polymerase II (Pol II), the mediator coactivators, and chromatin remodelling complex, might be a mechanistic target responsible for the metabolic disturbance. GTFs assemble at core promoter regions in a defined sequential order to essentially mediate the recruitment and activation of Pol II. Among the components involved in this process, Sub1, also known as PC4, functions as a transcriptional coactivator that binds DNA in a sequence-independent manner.^6^ Sub1 is an evolutionarily conserved nuclear protein that mediates PIC formation via recruitment of GTFs.^7^ Sub1 directly binds to TFIIB, TFIIEβ as a TFIIE subunit, TFIIFα and TFIIFβ as a TFIIF subunit, and XPB as a TFIIH subunit.^8^ Using unbiased chromatin immunoprecipitation-sequencing (ChIP-seq) analyses, we have previously demonstrated that the recruitment of Pol II to metabolic gene promoter is inhibited in pressure overload (PO)-induced heart failure. Thus, the impairment of PIC formation may contribute to metabolic remodelling during heart failure.^9^ However, the molecular mechanism through which the recruitment of the PIC to the metabolic gene promoters remains to be elucidated.

Sirt1 is an NAD^+^-dependent protein deacetylase that regulates transcription of metabolic genes. Although Sirt1 generally plays a protective role in many organs, including the heart, during stress,^10,11^ overexpression of Sirt1 in the heart causes cardiac dysfunction at baseline in a dose-dependent manner,^12^ which is accompanied by downregulation of nuclear-encoded mitochondrial genes.^13^ Furthermore, Sirt1 is upregulated in the heart and mediates cardiac dysfunction as well as metabolic disturbances during PO.^13,14^ Specifically, PO-induced cardiac hypertrophy, assessed by heart weight-to-body weight ratio, was significantly attenuated in Sirt1^+^/^−^ mice at 4 weeks.^13^ In addition, cellular hypertrophy was also reduced.^13^ Since both Sirt1-mediated downregulation of mRNA expression and PO-induced downregulation of Pol II recruitment to their promoters are commonly observed in metabolic genes, we hypothesized that upregulation of Sirt1 during PO inhibits Pol II recruitment to metabolic genes during heart failure.^9^

The goal in this study was to investigate the molecular mechanism through which upregulation of Sirt1 downregulates genes involved in fatty acid oxidation (FAO) and mitochondrial oxidative phosphorylation during PO-induced cardiac hypertrophy. We investigated molecular mechanisms through which Sirt1 inhibits Pol II recruitment to the metabolic gene promoter. We discovered that Sirt1 interacts with Sub1, a nuclear protein involved in the recruitment of the PIC, thereby inhibiting the recruitment of Pol II to transcription factors, including PPARα and ERRα involved in FAO and mitochondrial oxidative phosphorylation.

## 2. Methods

### 2.1 Animals

Cardiac-specific Sirt1 heterozygous knock-out mice (C57BL/6) were generated using αMHC-Cre and Sirt1^flox/+^ alleles. Cardiac-specific Sirt1 overexpression mice were generated using the α-myosin heavy chain promoter (Tg-Sirt1, line 40), and a tetracycline response element–driven Sirt1 transgenic line (line 12) on an FVB background exhibiting basal overexpression without induction was also used. Non-transgenic littermates served as controls. All animal protocols were approved by the Institutional Animal Care and Use Committee of Rutgers New Jersey Medical School and conformed to the NIH Guide for the Care and Use of Laboratory Animals.

### 2.2 Transverse aortic constriction

Transverse aortic constriction (TAC) was performed as previously described to induce PO.^15^ Mice were anaesthetized with isoflurane inhalation (5% for induction and 1% for maintenance), intubated, and mechanically ventilated. Adequate depth of anaesthesia was confirmed by the absence of the pedal withdrawal reflex throughout the procedure. The transverse aorta was constricted between the innominate and left common carotid arteries using a 7-0 prolene suture tied against a 26- or 27-gauge needle, which was subsequently removed. Sham-operated mice underwent the same procedure without aortic constriction. At the experimental endpoints, animals were killed by cervical dislocation under deep isoflurane anaesthesia.

### 2.3 Echocardiography

Echocardiography was performed in isoflurane-anaesthetized mice using a high-frequency ultrasound system (13-MHz transducer). Two-dimensional guided M-mode images were used to measure left ventricular (LV) dimensions, and LV ejection fraction and fractional shortening were calculated.

### 2.4 ChIP-seq

ChIP-seq of Pol II was described previously.^16^ GSE5637 is the accession number of the ChIP-seq data, which is available on the Gene Expression Omnibus Website (https://www.ncbi.nlm.nih.gov/geo/query/acc.cgi?acc=GSE50637).

### 2.5 Fatty acid oxidation

FAO was assessed in ventricular tissue homogenates by measuring the production of ^3^H_2_O from [^3^H]-oleic acid. Briefly, tissue homogenates were incubated with a reaction buffer containing [^3^H]-oleic acid at 37°C, and the reaction was terminated with chloroform/methanol extraction. The aqueous phase containing ^3^H_2_O was collected, and radioactivity was quantified by scintillation counting.

### 2.6 Immunoblot analyses

Heart homogenates were prepared using a lysis buffer [50 mM Tris–HCl (pH 7.6), 1% Triton X-100, 10 mM EDTA, 150 mM NaCl, 50 mM NaF, 100 µM dithiothreitol, and protease inhibitor cocktail (Sigma)]. Equal amounts of protein (10–20 μg) were subjected to 7–14% SDS–PAGE. After proteins were transferred to a polyvinylidene fluoride membrane, immunoblots were probed with the indicated antibodies, including TFIIB(GTF2B) (Proteintech, 16467-1-AP), XPB (Novus, NB100-61060), SUB1 (Novus, NB100-59775), TFIIEβ (Novus, NBP1-87931), Sirt1 (EMD Millipore, 07-131), Pol II (CST, 14958S), NF-κB p65 (D14E12) (CST,8242S), Histone H3 (D2B12) (CST, 4620S), PGC-1α (Millipore Ab3242), PPARα (Cayman 101710), ERRα (Millipore ERR46Y), and Tubulin (Sigma, T6199). The immunoblot signal intensities were quantified using the ImageJ program.

### 2.7 Primary cultures of neonatal rat ventricular myocytes

Primary neonatal rat ventricular myocytes were isolated from 1-day-old rats and used for *in vitro* experiments following culture and treatment with reagents or adenovirus.

### 2.8 Adenovirus vectors

Adenoviral vectors were generated using the AdMax system (Microbix), and cardiomyocytes were utilized 2–5 days following transduction.

### 2.9 siRNA for Sub1 and Sirt1

siRNA targeting Sub1 (Qiagen, S02006466) and Sirt1 (Ambion, AM16708) was used for gene knockdown, with a non-targeting siRNA as control (Qiagen, 1022076). Cells were transfected using Lipofectamine RNAiMAX (Invitrogen), and experiments were performed 24 h after transfection.

### 2.10 Purification of bacterially expressed proteins

GST-tagged Sirt1 and its truncation mutants were expressed in BL21 *E. coli* and purified using glutathione-Sepharose affinity chromatography. Recombinant Sub1 protein was purchased commercially (Sino Biological, 14326-H07E).

### 2.11 *In vitro* DNA-binding assay

Protein–DNA interactions were assessed by a biotinylated DNA pull-down assay using a 380-bp Idh3a promoter fragment containing the transcription start site (TSS). Cardiomyocytes with Sirt1 overexpression or knockdown, or Sub1 knockdown, were lysed and incubated with the biotinylated DNA. Protein–DNA complexes were captured with streptavidin beads and analysed by Western blot.

### 2.12 Docking simulation

Computational docking simulations were performed by Creative Proteomics. Three-dimensional Sirt1 and Sub1 structures were solved and used for the simulation.^17,18^ The flexible protein-protein docking model was constructed with the LightDock programme, followed by coarse-grained-based free energy perturbations.^19^

### 2.13 Statistics

All values in tables and graphs are expressed as the mean ±S.E. Normality was tested with the Shapiro–Wilk or normality test. If the data exhibited a normal distribution, pairwise testing was performed with the two-tailed Student’s *t* test. Multiple group comparisons were performed by one-way ANOVA, followed by Tukey post-test. If the data failed normality testing, pairwise testing was performed with the non-parametric Mann–Whitney *U* test, and multiple group comparisons were performed by the non-parametric Kruskal–Wallis test, followed by Dunn’s post-test. A priori power calculations were performed based on data from published studies. *P* < 0.05 was defined as statistically significant and indicated by a filled asterisk. Mice that did not survive the TAC surgical procedures were excluded from the data.

## 3. Results

### 3.1 Pol II recruitment to specific metabolic gene promoters is downregulated during PO

We have shown previously that the recruitment of Pol II to genes regulated by PGC-1α is downregulated in the heart during PO, as evaluated with ChIP-seq analyses.^9^ To test whether downregulation of Pol II recruitment during PO occurs more frequently in groups of genes known to be downregulated during PO-induced cardiac hypertrophy, we extended bioinformatic analyses of the ChIP-seq data. After 4 days of PO induced by TAC, the Pol II occupancy at promoters of metabolic genes involved in fatty acid catabolism, the tricarboxylic acid (TCA) cycle, and the electron transport chain was decreased, indicating reduced Pol II recruitment in the PO model (*Figure 1A*). In contrast, RNA polymerase II recruitment to promoters of genes involved in other cellular functions, such as necrosis, remained largely unchanged, serving as a control to demonstrate that the reduced Pol II occupancy was not a global phenomenon affecting all gene classes (*Figure 1B*). In our previous work, ChIP-qPCR analyses showed the Pol II recruitment to the promoter of genes involved in fatty acid catabolism, the TCA cycle, and the electron transport chain, such as *Mcad*, *Idh3a*, *Sdha*, and *Atp5k,* was inhibited after 2 weeks of PO.^9^ We extended this observation and confirmed that the inhibition of the Pol II recruitment to *Cpt2, Mcad, Idh3a*, and *Atp5k* promoters was observed also after 4 weeks of PO (*Figure 1C*). Since downregulation of the Pol II recruitment occurs in those known to be downregulated during the chronic phase of PO, we hypothesized that the upstream molecular mechanism inhibiting the Pol II recruitment to the promoters of the genes involved in fatty acid catabolism, the TCA cycle, and the electron transport chain plays a key role in mediating the metabolic remodelling during PO.

**Figure 1:**
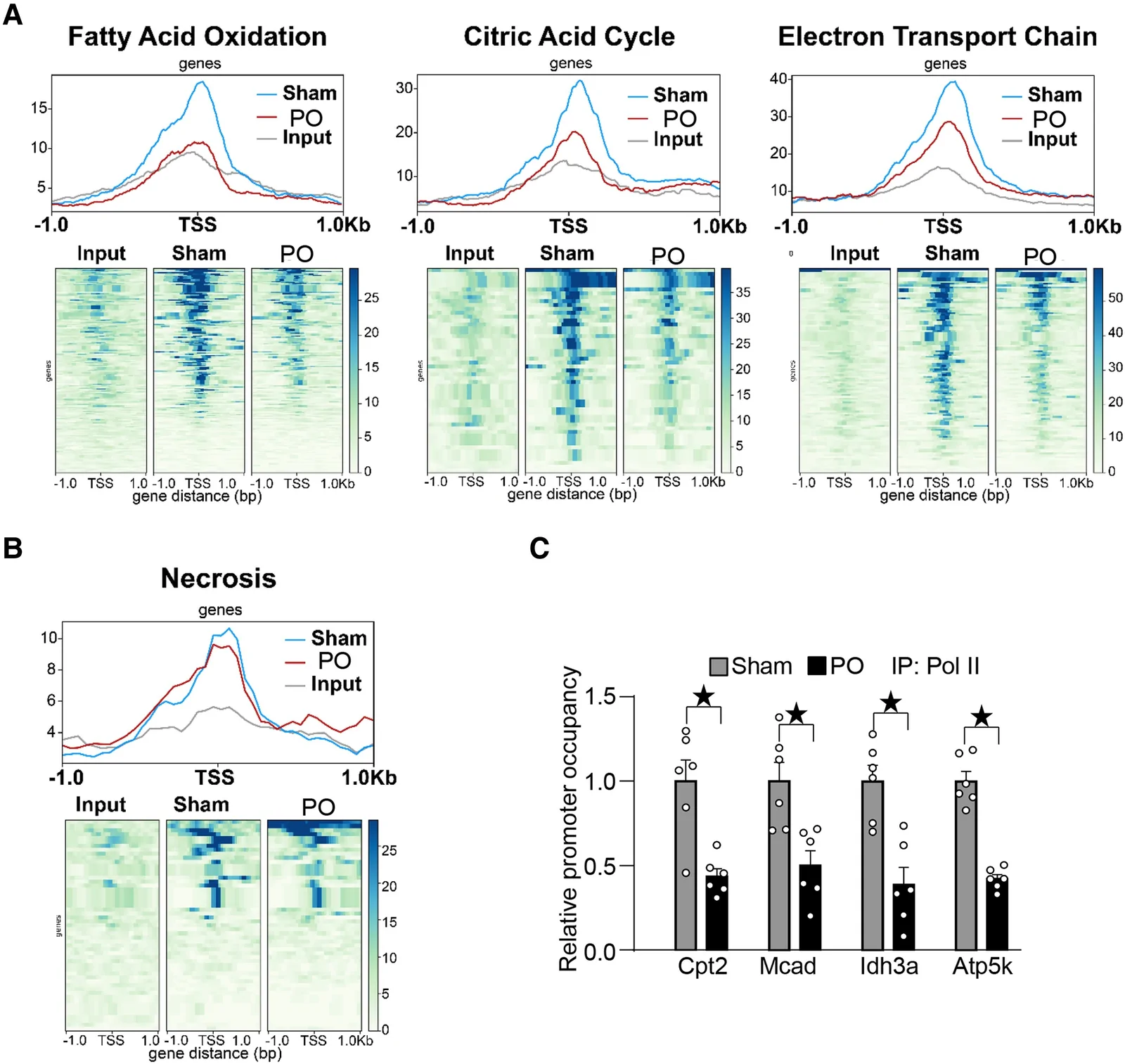
Pol II recruitment to metabolic gene promoter is inhibited in the PO model. (*A, B*) ChIP-seq with Pol II antibody was performed using mouse hearts subjected to 4 days of PO. Average Pol II occupancies in the promoter regions of genes involved in the fatty acid catabolic process, TCA cycle, electron transport chain, and necrosis (MSigDB (Molecular Signatures Database)) were estimated with bioinformatics analyses using the ChIP-seq data. (*C*) Pol II recruitment to the promoters of Cpt2, Mcad, Idh3a, and Atpk5 is inhibited during the 4 weeks of PO. *N* = 6. Statistical significance was determined with Student’s *t*-test.

### 3.2 The recruitment of Sirt1 to metabolic gene promoters in response to PO

What is the signalling mechanism through which the recruitment of Pol II to the metabolic gene promoter is inhibited during PO? In this study, we focused on Sirt1 because we and others have shown previously that endogenous Sirt1 negatively regulates expression of genes involved in fatty acid catabolism, the TCA cycle, and the electron transport chain during PO.^13,14^

As reporterted previsouly,^12,20^ Sirt1 protein levels are upregulated in the cytosol and nucleus after 4 weeks of PO (*Figure 2A* and *B*), and we confirmed significant upregulation after 4 days of PO as well (*Figure 2A*). Since Sirt1 can affect transcription of genes through interaction with the gene promoter,^5^ we analysed available ChIP-seq data conducted with a Sirt1 antibody to investigate whether Sirt1 interacts with gene promoters. The recruitment of Sirt1 to the promoters of genes involved in FAO, the TCA cycle, and the electron transport chain was increased in response to 2 weeks of PO (*Figure 2C*). In contrast, Sirt1 recruitment to promoters of necrosis-related genes was minimal under both basal and PO conditions compared to metabolic genes (*Figure 2D*). *Figure 2E* shows the Sirt1 recruitment to representative metabolic gene promoters (*Cpt2*, *Mcad*, *Idh3a*, and *Atp5k*) after 2 weeks of PO. ChIP-qPCR confirmed increased Sirt1 binding to these promoters in the mouse heart after 4 weeks of PO (*Figure 2F*). These results suggest that Sirt1 localizes to the metabolic gene promoter during PO.

**Figure 2:**
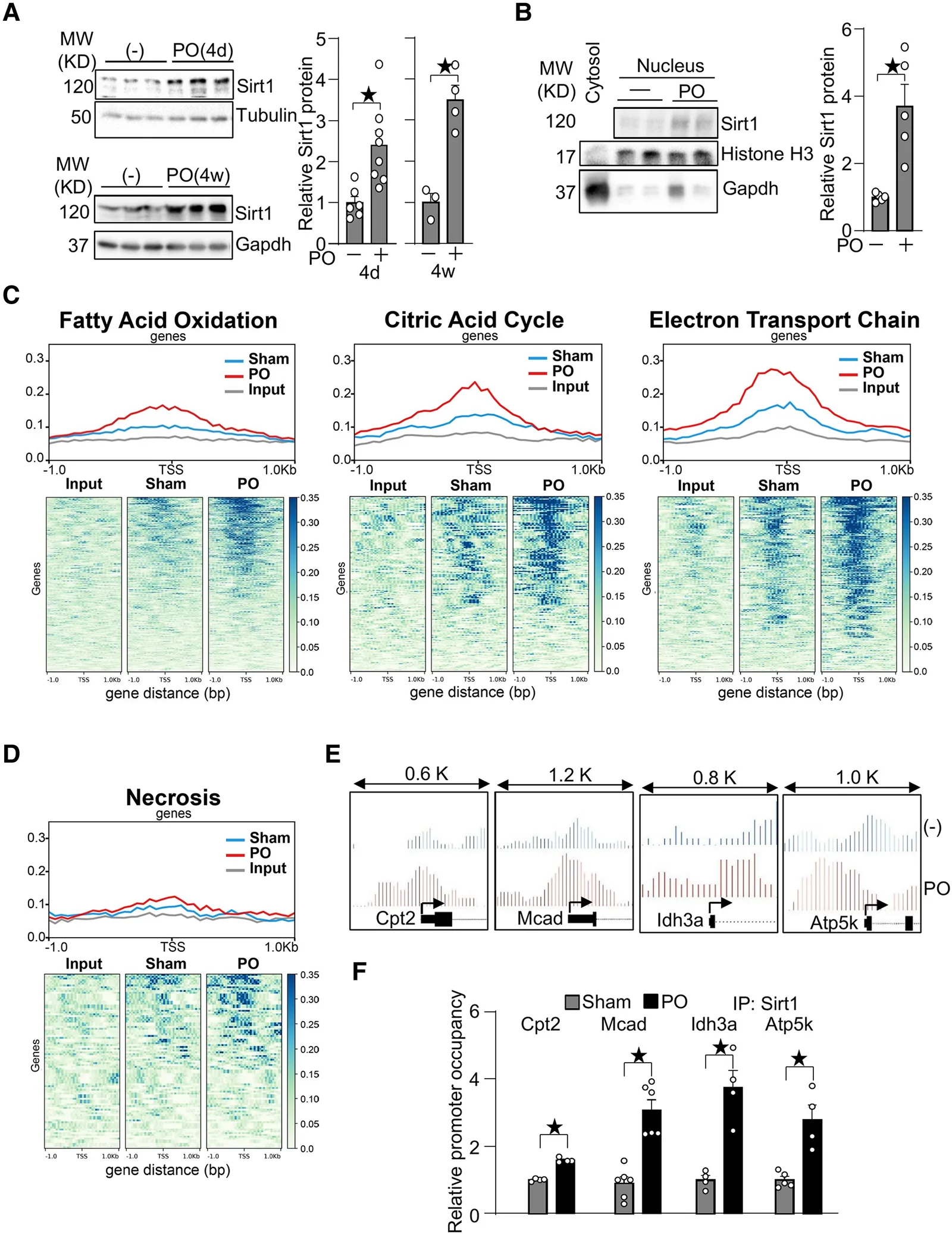
Sirt1 is recruited to metabolic gene promoters in response to PO. (*A*) Sirt1 expression is upregulated in the heart at both 4 days and 4 weeks of PO. (*B*) Sirt1 expression is upregulated in the nuclear fraction of the heart at 4 weeks of PO. (*C–E*) Sirt1 is recruited to metabolic gene promoters in response to PO. ChIP-seq with Sirt1 antibody was performed using mouse hearts subjected to 2 weeks of PO. Average Pol II occupancies in the promoter regions of genes involved in the fatty acid catabolic process, TCA cycle, electron transport chain (*C*), and necrosis (*D*) (MSigDB (Molecular Signatures Database)) were estimated with bioinformatics analyses using the ChIP-seq data. (*E*) Sirt1 occupancies in indicated gene promoters are shown by ChIP-seq. (*F*) Sirt1 occupancy at target gene promoters after 4 weeks of PO, assessed by ChIP-qPCR. The numbers of mice examined in each experimental group were 6–8 (*A*, 4d), 3–4(*A*, 4w), 5(*B*), and 4–6 (*F*). Statistical significance was determined with Mann–Whitney *U* test (*F*, Mcad) and Student’s *t*-test for all other comparisons.

### 3.3 Sirt1 inhibits the Pol II recruitment to the metabolic gene promoters during PO

To investigate whether endogenous Sirt1 regulates Pol II recruitment, cardiac-specific Sirt1 heterozygous knock-out (Sirt1*^+/−^*) mice were subjected to PO. ChIP assays using a Pol II antibody revealed that PO inhibited Pol II recruitment to metabolic gene promoters (*Cpt2, Atp5k,* and *Idh3a*) after 4 weeks of PO in wild-type mice, whereas this effect was reversed in Sirt1*^+/−^* mice (*Figure 3A*). Pol II recruitment to the *Mcad* promoter was significantly enhanced in Sirt1*+/−* mice under PO conditions (*Figure 3A*). To further examine the inhibitory role of Sirt1 on Pol II recruitment, ChIP-qPCR was performed in cardiac-specific Sirt1-overexpressing (Tg-Sirt1) mice. Sirt1 overexpression alone was sufficient to induce its recruitment to the promoters even in the absence of PO, while concurrently inhibiting Pol II recruitment at the same sites (*Figure 3B*). Collectively, these findings suggest that Sirt1 is recruited to metabolic gene promoters, where it inhibits Pol II recruitment in response to PO.

**Figure 3:**
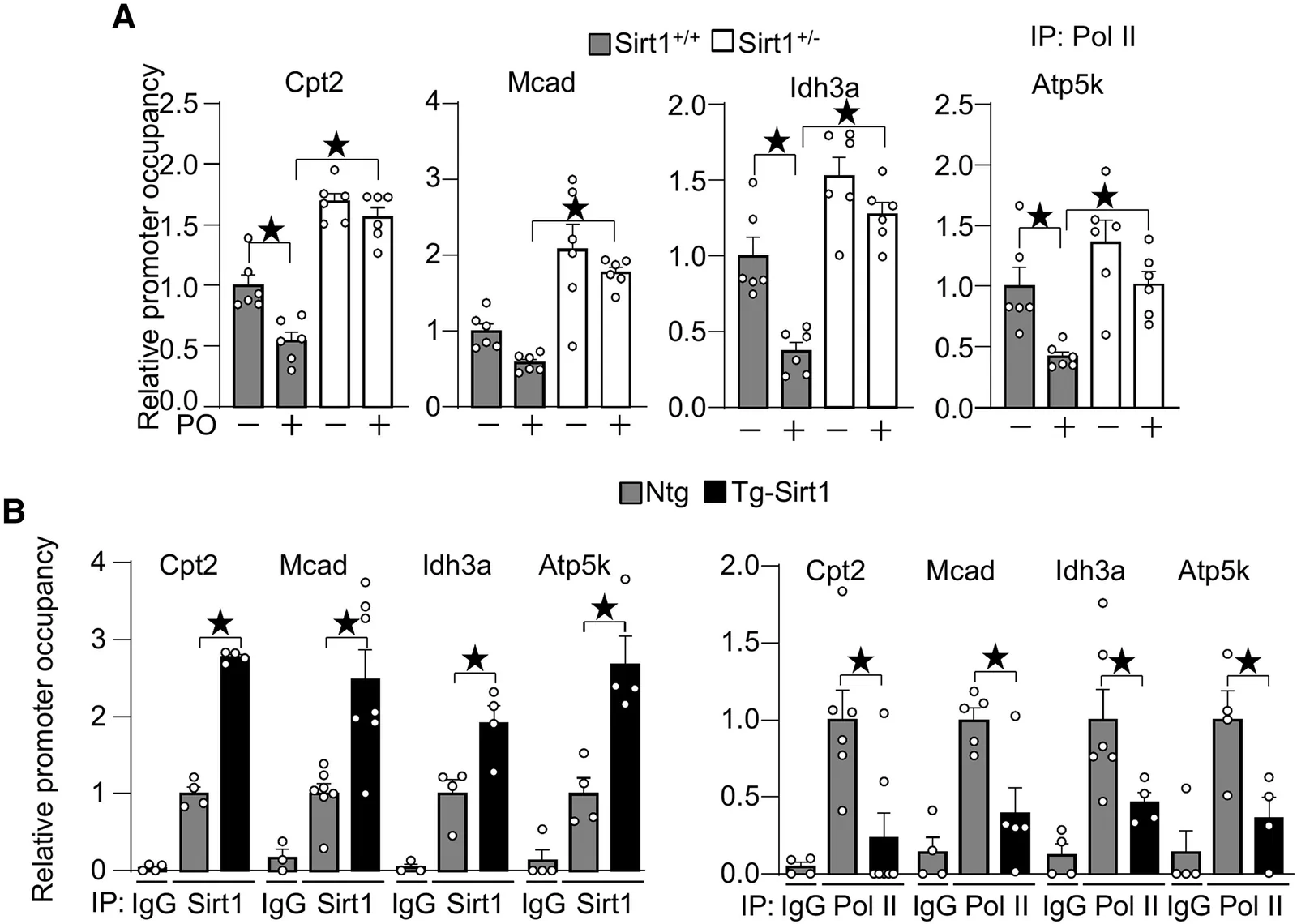
Sirt1 inhibits Pol II recruitment in a PO-induced heart failure model. (*A*) Pol II recruitment, inhibited by PO, was restored in Sirt1^+/−^ mice. (*B*) Tg-Sirt1 mice showed increased Sirt1 and decreased Pol II recruitment. The numbers of mice examined in each experimental group were: 6 (*A*) and 3–7. Statistical significance was determined with ANOVA (*A*), Student’s *t*-test (*B*: Sirt1-IP, Idh3a, Atp5k; *C*: Pol II-IP, Cpt2), Mann–Whitney *U* test for all other comparisons.

### 3.4 Sirt1 suppresses metabolic genes possibly through inhibition of Pol II recruitment

To validate the Pol II dynamics observed in PO and Sirt1 genetic models, we analysed mRNA levels. Under PO conditions, the expression of metabolic genes involved in fatty acid metabolism (e.g. *Cpt2* and *Mcad*) and mitochondrial ATP production (e.g. *Atp5k*, *Idh3a*, *Sdha*, *Sdhd*, and *Ndufv1*) was impaired. However, as reported previously,^5,13^ this dysregulation was normalized in Sirt1*^+/−^* mice (see Supplementary material online, *Figure S1A*). Additionally, *Ndufs1* expression followed the same trend (see Supplementary material online, *Figure S1A*). Sirt1 overexpression alone significantly inhibited the expression of several metabolic genes, including *Mcad*, *Atp5k*, *Idh3a*, and *Ndufs1*, while a similar trend was observed for the other genes examined (see Supplementary material online, *Figure S1B*). Furthermore, Sirt1 overexpression enhanced PO-induced downregulation of *Cpt2* and *Idh3a*, with a comparable trend seen in other genes analysed (see Supplementary material online, *Figure S1B*). Additionally, Sirt1 overexpression exacerbated PO-induced downregulation of mitochondrial genes at protein levels such as *Atp5a1*, *Uqcrc2*, *Sdhb*, and *Ndufb8* (see Supplementary material online, *Figure S1C*). These findings suggest that Sirt1 inhibits Pol II recruitment, thereby suppressing the expression of metabolic genes.

### 3.5 Sirt1 inhibits metabolic ability

To determine whether Pol II dynamics observed in PO and Sirt1 genetic models are associated with metabolic function and cardiac performance, Sirt1^+/−^ and Tg-Sirt1 mice were subjected to four weeks of PO. FAO was assessed using [^3^H]-labelled oleic acid. PO significantly reduced FAO in wild-type mice but not in Sirt1^+/−^ mice (*Figure 4A*). To investigate the pathological metabolic shift involving glycolysis, as well as mitochondrial glucose and FAO, we assessed the expression of pyruvate dehydrogenase kinase 4 (PDK4), a key regulator that promotes glycolysis and FAO by inhibiting glucose oxidation. *Pdk4* expression tended to increase under PO conditions. Importantly, *Pdk4* levels were significantly lower in Sirt1^+/−^ hearts compared to wild-type mice under PO (*Figure 4B*). Given that FAO is inhibited in the PO models (*Figure 4A*), the trend towards upregulation of Pdk4 likely reflects both a compensatory mechanism to support FAO and a shift towards glycolytic metabolism. These findings suggest that such pathological metabolic reprogramming is attenuated in Sirt1^+/−^ mice. Consistent with our previous report,^13^ PO-induced cardiac dysfunction, indicated by reduced fractional shortening (FS), was normalized in Sirt1^+/−^ mice (*Figure 4C*). Similarly, Sirt1 overexpression exacerbated PO-induced impairment of FAO (*Figure 4D*). As previously reported,^14^ Sirt1 overexpression also worsened PO-induced heart failure phenotypes, characterized by increased mortality (see Supplementary material online, *Figure S2A* and *E*), impaired contractile function (see Supplementary material online, *Figures S1F* and *S2B*), lung congestion (see Supplementary material online, *Figure S2C* and *G*), and cardiac hypertrophy (see Supplementary material online, *Figure S2D* and *H*). These adverse effects were consistent across two independent Tg-Sirt1 lines (lines 12 and 40) (see Supplementary material online, *Figure S1I* and *J*). Notably, Tg-Sirt1 line 12 exhibited similar myocyte size (see Supplementary material online, *Figure S2K*) but more severe fibrosis (see Supplementary material online, *Figure S2L*). To determine whether Sirt1 negatively regulates gene expression and mitochondrial respiration in a cell-autonomous manner, primary cultured cardiomyocytes were transduced with an adenoviral vector to overexpress Sirt1. This overexpression led to a reduction in mitochondrial protein levels (*Figure 4E*). Additionally, Sirt1 suppressed basal respiration, ATP production–coupled respiration, and maximal oxygen consumption in cultured cardiomyocytes (*Figure 4F*). Together, these findings suggest that Sirt1 exacerbates PO-induced metabolic disturbances and cardiac dysfunction, potentially by inhibiting Pol II recruitment.

**Figure 4:**
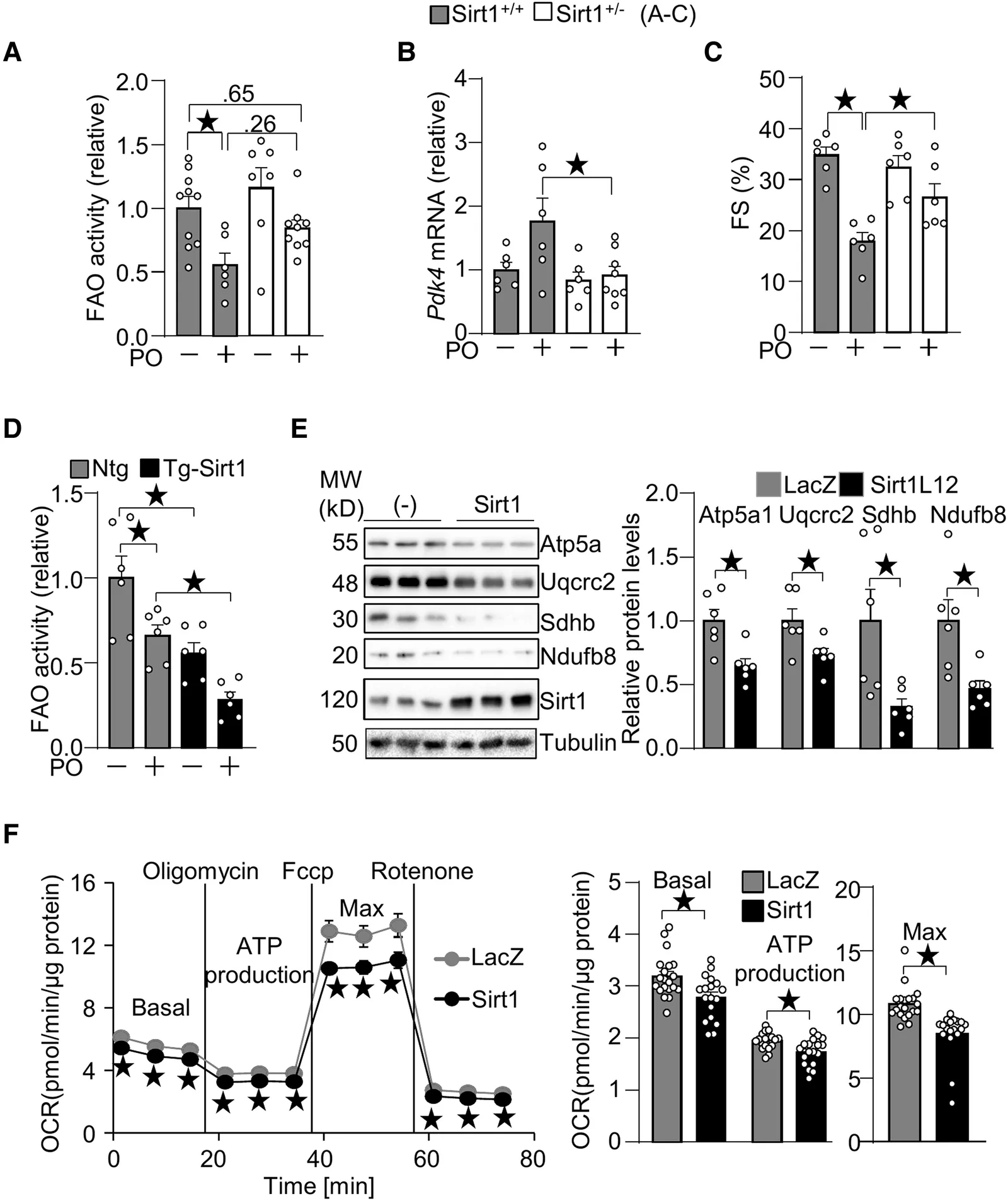
Sirt1 mediates PO-induced metabolic disturbance and cardiac dysfunction. (*A*) PO-induced inhibition of FAO was normalized in Sirt1^+/−^ mice. (*B*) The mRNA level of *Pdk4* was suppressed in Sirt1^+/−^ mice under PO conditions. (*C*) PO-induced contractile dysfunction was normalized in Sirt1^+/−^ mice. (*D*) Cardiac-specific Sirt1 overexpression impairs FAO. The numbers of mice examined in each experimental group were 6–10 (*A*), 6–8 (*B*), 6 (*C*), and 6 (*D*). (*E*) Sirt1 suppresses mitochondrial proteins in cardiomyocyte cell autonomous manner. *N* = 6. (*F*) Sirt1 inhibits mitochondrial oxygen consumption. *N* = 18–20. Statistical significance was determined with ANOVA (*A–C*), Student’s *t*-test (*D*, *E* (basal and ATP production)), and Mann–Whitney *U* test for all other comparisons.

### 3.6 Sirt1 interacts with Sub1 that mediates PIC formation in metabolic gene promoters

To clarify the mechanism by which Sirt1 inhibits Pol II recruitment, we screened Sirt1 binding proteins in cultured cardiomyocytes. HA-tagged Sirt1 was expressed in cultured cardiomyocytes using an adenovirus vector, and Sirt1 was immunoprecipitated with an anti-HA antibody. The Sirt1 immunoprecipitate was then subjected to mass spectrometric analyses to identify Sirt1-binding proteins. Using liquid chromatography-tandem mass spectrometry (LC-MS/MS) and the spectral counting method, we identified 739 proteins by LC-MS/MS, of which 528 proteins bound well to HA-Sirt1 (Ratio >1.5) with respect to the control group (see Supplementary material online, *Table S1*). The LC-MS/MS analysis showed that Sub1 was the protein that bound abundantly to Sirt1 in the cardiomyocytes. Sub1 is an evolutionarily conserved nuclear protein that mediates PIC formation via recruitment of GTFs.^7^ Sub1 directly binds to TFIIB, TFIIEβ as a TFIIE subunit, TFIIFα and TFIIFβ as a TFIIF subunit, and XPB as a TFIIH subunit.^8^ Co-immunoprecipitation assays showed that Sirt1 binds to Sub1 but not to Pol II or TFIIB, indicating a specific interaction between Sirt1 and Sub1 (*Figure 5A*). To investigate whether Sirt1 binds to Sub1 *in vitro*, we generated GST-Sirt1 and truncation mutants (*Figure 5B*). As shown in *Figure 5C*, full length Sirt1 bound to Sub1 *in vitro*, suggesting that Sirt1 directly binds to Sub1. In addition, the catalytic domain of Sirt1 strongly bound to Sub1, and N-terminal Sirt1 also bound to Sub1 to some extent. Although the apparent electrophoretic mobility of the Sirt1 truncation mutants did not strictly correspond to their predicted molecular weights based on amino acid length, anomalous migration of Sirt1 proteins in SDS-PAGE analyses has been commonly observed. In particular, the C-terminal fragment migrated substantially slower than expected despite being the shortest construct. Notably, full-length Sirt1 itself, whose predicted molecular weight is approximately 80 kDa, is widely observed to migrate near ∼120 kDa in SDS-PAGE analyses.^21^ One possible explanation is that the C-terminal region contains acidic and structurally disordered sequences that alter electrophoretic mobility, and this region may contribute substantially to the anomalously slow migration behaviour of Sirt1 proteins. Computational modelling with the LightDock program^19^ followed by coarse-grained-based free energy perturbations predicted several docking models. One model showed Sub1 binding to the N-terminal and catalytic domain of Sirt1, which corresponds well to the results of the *in vitro* binding assay (*Figure 5D*). Since Sub1 not only activates but also inhibits transcription in a context dependent manner,^22^ we examined the ability of Sub1 to promote transcription, using reporter genes driven by the endogenous promoters of metabolic genes and by the interleukin-6 (*Il-6*) as a control representing the non-metabolic gene. As shown in *Figure 5E*, the activity of reporter genes driven by the *Mcad, Ndufs1,* and *Ndufv1* promoters was inhibited in the presence of Sub1 knockdown. In sharp contrast, the activity of a reporter gene driven by the *Il-6* promoter was promoted by Sub1 knockdown. Knockdown of Sub1 inhibited the expression of metabolic genes such as *Mcad, Ndufs1,* and *Ndufv1* but promoted that of *Il-6* (*Figure 5F*). These results suggest that endogenous Sub1 is a positive transcriptional regulator of some metabolic genes but that may not be the case for other genes. To gain further insight into how Sub1 specifically promotes metabolic gene expression, we investigated whether Sub1 binds to specific nuclear factors that regulate transcription of metabolic genes, including PGC-1α, PPARα, and ERRα, and to the RelA subunit of NF-κB as a non-metabolic control. Flag-Sub1 was expressed in cultured cardiomyocytes using an adenovirus vector and immunoprecipitated with an anti-Flag antibody. Although Sub1 did bind to PPARα and ERRα, significant binding to PGC-1α and RelA was not observed, suggesting that Sub1 functions cooperatively with a subset of STFs for metabolic genes, including PPARα and ERRα (*Figure 5G*). To test whether Sub1 mediates the PIC formation at a metabolic gene promoter*, in vitro* DNA binding assays were conducted using biotin-labeled DNA comprising the endogenous *Idh3a* promoter and cardiomyocyte lysate as a source of GTFs and Pol II (*Figure 5H*). The biotinylated (Bi)-Idh3a was incubated with cardiomyocyte lysate to form a PIC at the promoter. To evaluate the role of Sub1 in the cardiomyocyte lysate, DNA binding assays were conducted in the presence of siRNA targeting Sub1 (siSub1) or control siRNA. As shown in *Figure 5I*, Bi-Idh3a successfully pulled down Pol II and GTFs, such as XPB, TFIIB, and TFIIEβ, in the presence of control siRNA, indicating PIC formation. However, Bi-Idh3a failed to pull down Pol II and GTFs, such as XPB, in the presence of siSub1, suggesting that Sub1 is essential for PIC formation. Interestingly, the recruitment of Sirt1 to Bi-Idh3a was only partially inhibited by Sub1 knockdown, suggesting the existence of Sub1-independent mechanisms for Sirt1 recruitment. Potential Sirt1 recruiting factors, such as ERRs and PGC-1α, may account for this residual recruitment.^23^ Because the initial experiment did not clearly determine whether Sub1 directly recruits Sirt1, an additional binding assay was performed using recombinant Sirt1 and Sub1. While Sirt1 alone was unable to bind the Idh3a promoter, it successfully bound to the promoter in the presence of Sub1 (*Figure 5J*). This result suggests that Sub1 directly recruits Sirt1 to the Idh3a promoter. Taken together, these results suggest that Sirt1 interacts with Sub1 which mediates PIC formation.

**Figure 5:**
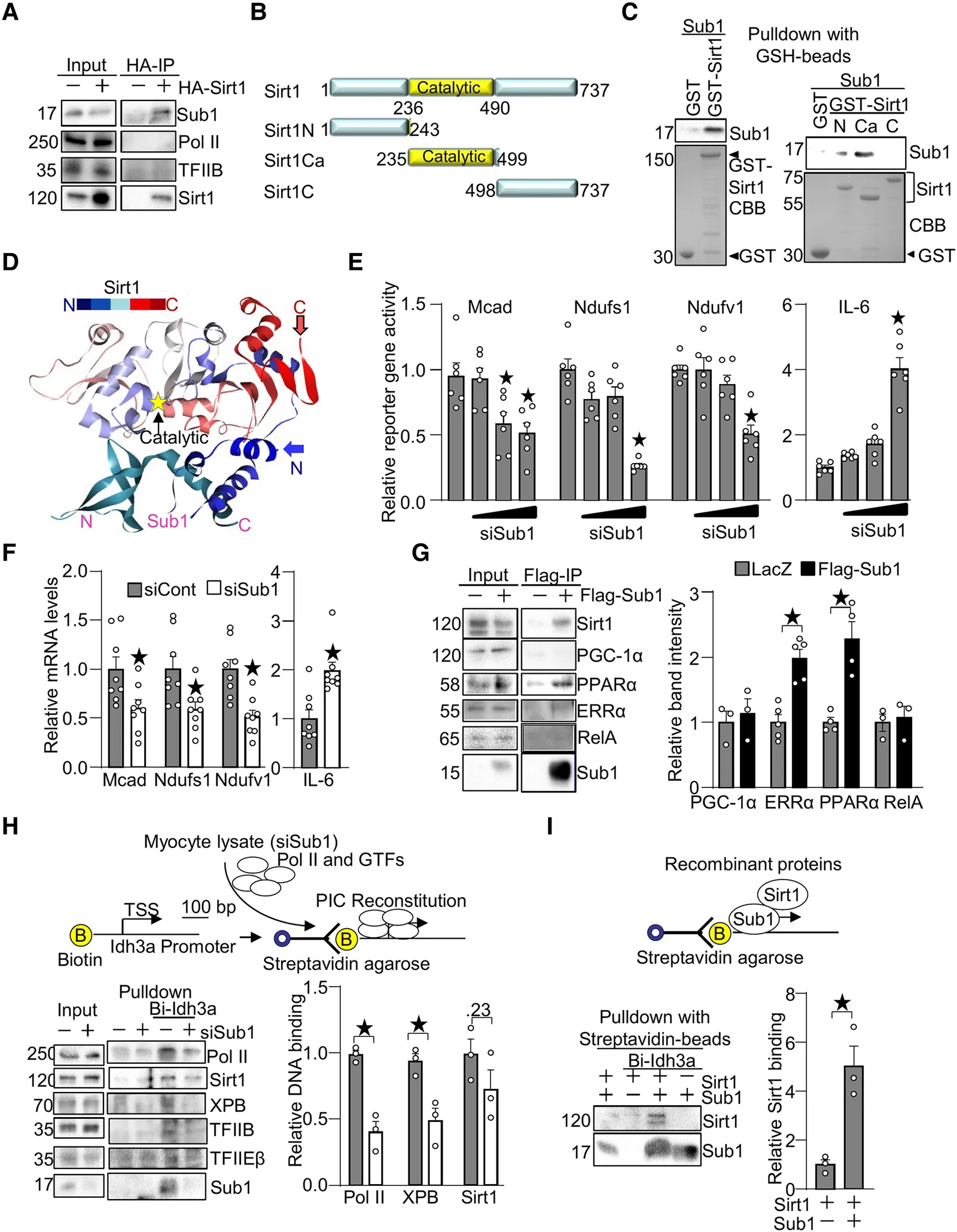
Sub1 is a Sirt1 binding protein. (*A*) Sirt1 binds to Sub1. HA-Sirt1 was expressed in cultured cardiomyocytes using an adenovirus vector and immunoprecipitated with anti-HA antibody. The immunocomplex was subjected to immunoblot with the indicated antibodies. (*B*) Schematic representation of Sirt1 recombinant proteins. (*C*) Sub1 binds to the N-terminal and catalytic domain of Sirt1 *in vitro*. (*D*) Computational model of binding between Sirt1 and Sub1. (*E, F*) Sub1 is required for metabolic gene promoter activity. (*E*) Reporter gene assays were performed using a luciferase reporter gene driven by the endogenous promoter sequences of the indicated genes. *N* = 6. (*F*) Knockdown of Sub1 suppresses mRNA expression of Mcad, Ndufs1, and Ndufv1 but not IL-6. *N* = 8. (*G*) Sub1 binds to PPARα and ERRα. Flag-Sub1 was expressed in cultured cardiomyocytes using an adenovirus vector and immunoprecipitated with anti-Flag antibody. The immunocomplex was subjected to immunoblot with the indicated antibodies. *N* = 3–5. (*H–I*) Sub1 is required for PIC formation in Idh3a promoter. (*H*) Schematic representation of the DNA-binding assay. Biotin-labeled Idh3a promoter containing ERR response elements (ERRE) and a transcription starting site (TSS) was incubated with cardiomyocyte lysates. The DNA-binding proteins were detected by Western blot analyses. (*I*) Sub1 is required for the recruitment of Pol II, XPB, TFIIB, and TFIIEβ to the Idh3a promoter *in vitro*. *N* = 3. (*J*) Sub1 recruits Sirt1 to the Idh3a promoter *in vitro*. Recombinant Sub1 and Sirt1 proteins were pulled down with the biotin-labelled Idh3a promoter. *N* = 3. Statistical significance was determined with ANOVA (*E*) and Student’s *t*-test for all other comparisons.

### 3.7 Sirt1 negatively affects the recruitment of the PIC to metabolic gene promoters without affecting the DNA binding of Sub1

DNA binding of Sub1 is promoted by acetylation.^24^ Since Sirt1 is a protein deacetylase, Sirt1 may deacetylate Sub1, thereby inhibiting DNA binding as well as PIC formation. Sirt1 binds to Sub1 partly through its catalytic domain (*Figure 5C*), while the computational binding model predicts that no region of Sub1 fits into the catalytic groove in Sirt1 (*Figure 5D*).

To determine whether Sirt1 deacetylates Sub1, Flag-Sub1 was expressed in cultured cardiomyocytes with an adenovirus vector. Acetylation of Sub1 was assessed with an anti-acetyl-lysine antibody after immunoprecipitation with an anti-Flag antibody. Overexpression of Sirt1 did not significantly decrease Sub1 acetylation (*Figure 6A*). In addition, Ex527, a Sirt1 inhibitor, did not significantly promote Sub1 acetylation (*Figure 6B*). These results suggest that Sirt1 does not significantly deacetylate Sub1 in cardiomyocytes. Because Sirt1 directly binds to Sub1, we next hypothesized that Sirt1 competitively inhibits the binding of Sub1 to GTFs, thereby inhibiting PIC formation.

**Figure 6:**
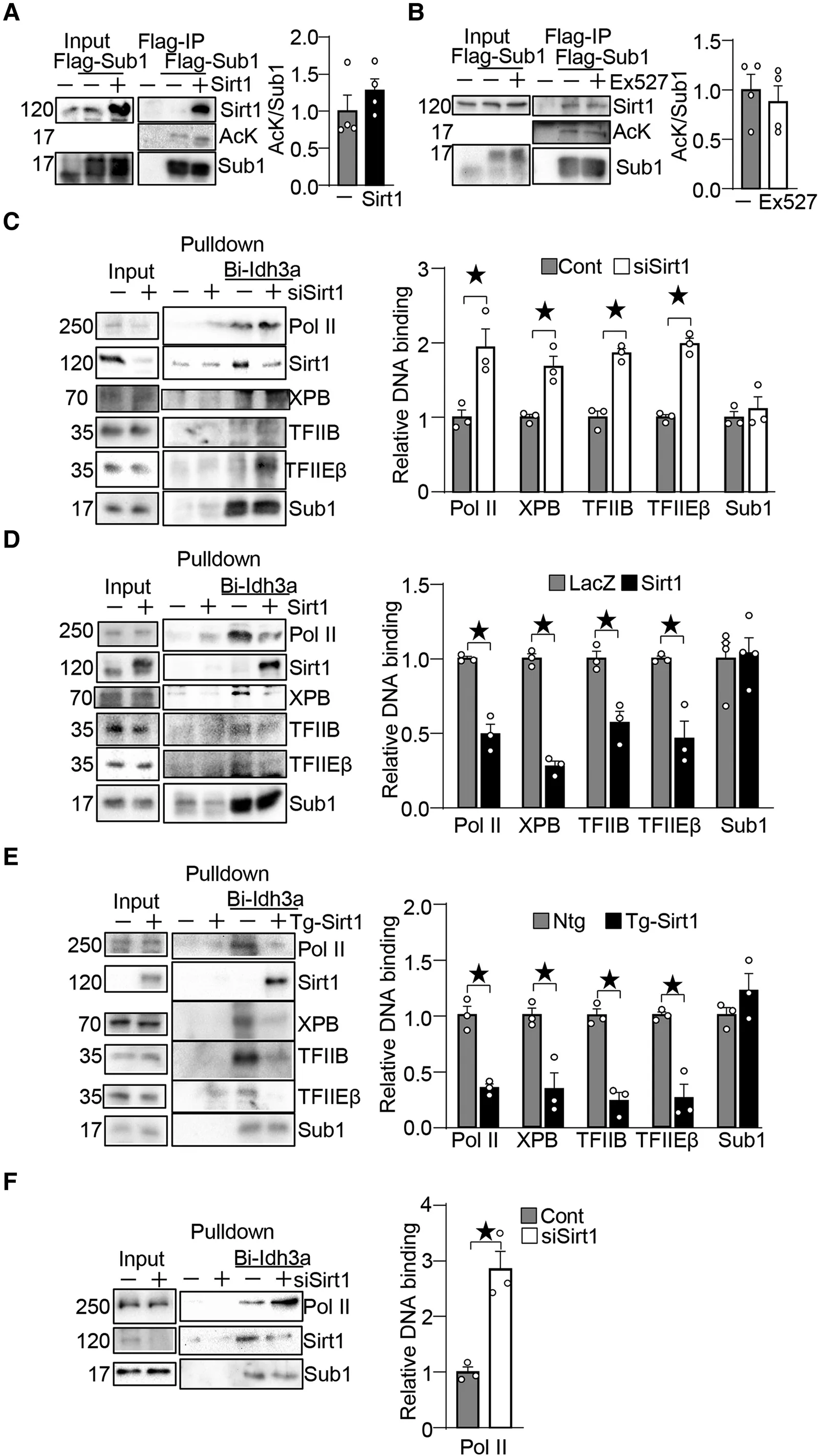
Sirt1 inhibits Sub1-induced PIC formation. (*A, B*) Sirt1 overexpression (*A*) and inhibition with Ex527 (*B*) did not affect Sub1 acetylation. *N* = 4. (*C*) Sirt1 knockdown promoted the recruitment of XPB, TFIIB, and TFIIEβ, but not Sub1, to the Idh3a promoter *in vitro*. *N* = 3. (*D*) Sirt1 overexpression inhibited the recruitment of Pol II, XPB, TFIIB, and TFIIEβ, but not Sub1, to the Idh3a promoter *in vitro*. *N* = 3–4. (*E*) Sirt1 overexpression inhibited the recruitment of Pol II, XPB, TFIIB, and TFIIEβ, but not Sub1, to the Idh3a promoter in the heart *in vivo*. *N* = 3. (*F*) Sirt1 knockdown promoted Pol II recruitment in AC16 cells. *N* = 3. Statistical significance was determined with Student’s *t* test for all comparisons.

To test whether Sirt1 directly inhibits PIC formation, DNA binding assays were performed using cardiomyocyte lysates. As shown in *Figure 6C* and *D*, Sirt1 overexpression inhibited the recruitment of Pol II, XPB, TFIIB, and TFIIEβ to the Bi-Idh3a promoter, whereas Sirt1 knockdown promoted their binding to the Idh3a promoter. Consistent with the fact that Sirt1 does not deacetylate Sub1, the binding of Sub1 to the Idh3a promoter was not significantly changed by Sirt1 overexpression or knockdown. Sirt1 also inhibited the recruitment of Pol II, XPB, TFIIB, and TFIIEβ, but not Sub1, to the Bi-Idh3a promoter in Tg-Sirt1 heart lysates (*Figure 6E*). Sirt1 overexpression of PO did not significantly affect the expression of Sub1, Pol II, or TFIIB (see Supplementary material online, *Figure S3*). To assess the relevance of these findings in human cardiomyocytes, we next examined AC16 cells. Consistent with the results obtained using cardiomyocyte lysates, Sirt1 knockdown enhanced Pol II recruitment to the Idh3a promoter (*Figure 6F*). These results suggest that Sirt1 competitively inhibits the recruitment of GTFs, but not Sub1, to the promoter, thereby inhibiting PIC formation.

### 3.8 Sirt1 competitively inhibits the binding of the GTFs to Sub1

To test whether Sirt1 competitively inhibits the binding of GTFs to Sub1, co-immunoprecipitation assays were performed. As shown in *Figure 7A*, Sirt1 overexpression significantly reduced Sub1 binding to XPB, TFIIB, and TFIIEβ in a dose-dependent manner. As shown in *Figure 7B*, knockdown of Sirt1 promoted the binding of Sub1 to XPB, TFIIB, and TFIIEβ. Again, knockdown of Sirt1 did not promote Sub1 acetylation. To test whether Sirt1 inhibits the binding of Sub1 to GTFs *in vitro*, a binding assay was performed with recombinant GST-TFIIB. The binding of Sub1 to TFIIB was competitively inhibited by Sirt1 in a dose-dependent manner *in vitro* (*Figure 7C*). To test whether Sirt1 inhibits transcription of metabolic genes through inhibition of a Sub1-dependent mechanism, reporter gene assays were performed. Sirt1 knockdown promoted reporter gene activity driven by endogenous *Mcad, Ndufa1, Ndufs1,* and *Ndufv1* promoters, an effect that was abolished in the presence of Sub1 knockdown (*Figure 7D*). Taken together, these results suggest that Sirt1 competitively inhibits the binding of Sub1 to GTFs, thereby inhibiting PIC formation at the metabolic gene promoter in the failing heart (*Figure 7E*).

**Figure 7:**
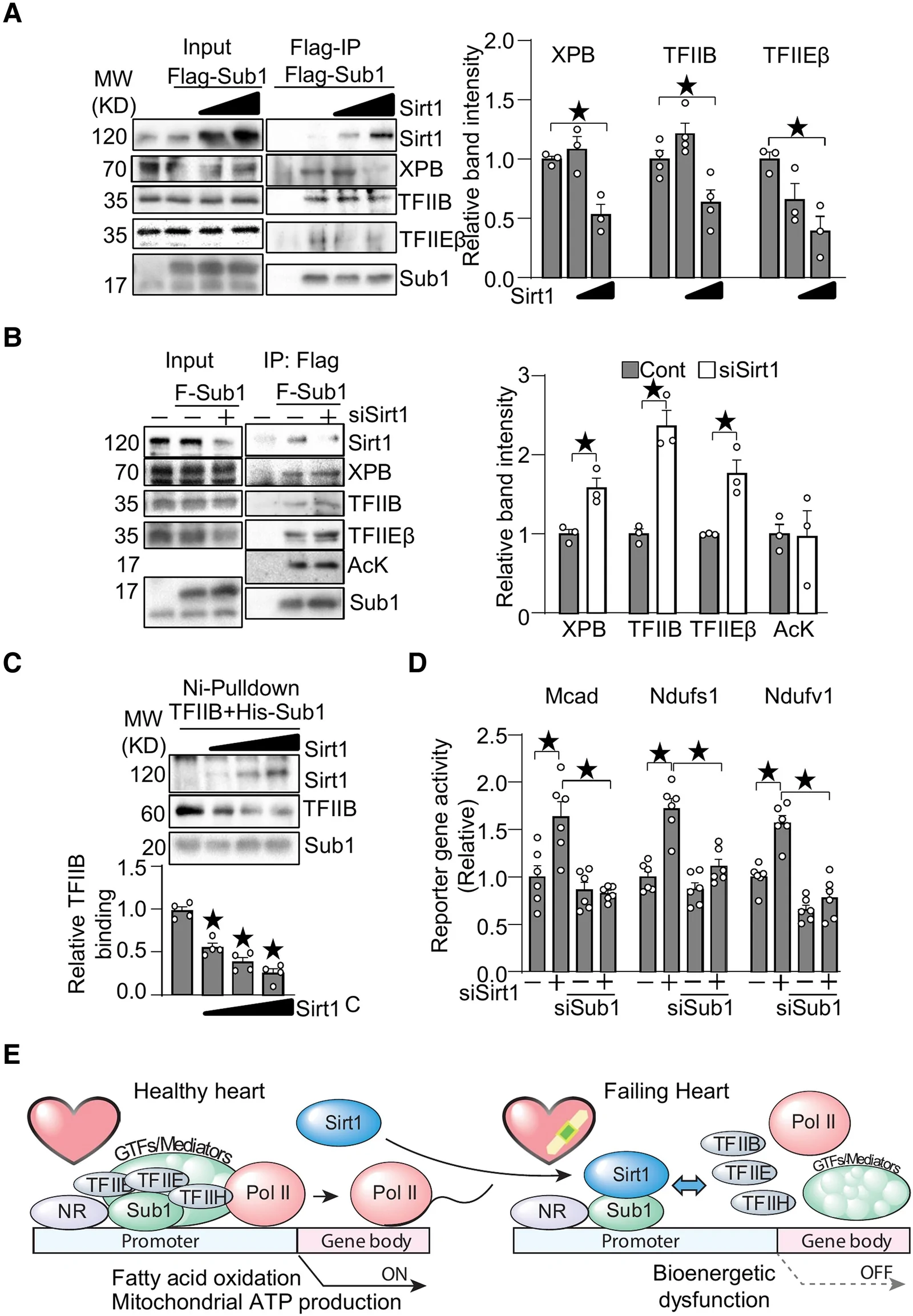
Sirt1 inhibits the binding of Sub1 to general transcription factors. (*A*) Sirt1 overexpression inhibited the binding of Sub1 to XPB and TFIIEβ. *N* = 3–4. (*B*) Sirt1 knockdown enhanced the binding of Sub1 to XPB and TFIIEβ. *N* = 3. (*C*) Sirt1 competitively inhibits the binding of Sub1 to TFIIB *in vitro*. *In vitro* binding assays were performed with His-Sub1, GST-TFIIB, Sirt1, and Nickel-agarose. *N* = 4. (*D*) Sirt1 inhibits transcription via Sub1. Reporter gene assays were performed with a luciferase reporter gene driven by the indicated gene promoters. *N* = 6. (*E*) Schematic representation of Sirt1-induced inhibition of Pol II recruitment. Statistical significance was determined with Student’s *t* test (*B*) and ANOVA for all other comparisons.

## 4. Discussion

Metabolic remodelling, including downregulation of genes involved in FAO, is commonly observed during the chronic phase of heart failure. Since downregulation of PPARα ERRα, and PGC-1α, which are involved in cardiac metabolism, is commonly observed during heart failure, much effort has focused on identifying their transcriptomes to normalize their function. However, rescuing the expression levels of the metabolic transcription factors alone often fails to achieve complete normalization of metabolic remodeling.^4,5^ Here, we show that dysregulation of the general transcriptional machinery is involved in the metabolic disturbance in the failing heart. Since the general transcriptional machinery is downstream of STFs, our results suggest that, besides the specific transcriptional machinery, interventions to normalize the function of the general transcriptional machinery need to be considered to address the metabolic remodelling during heart failure.

We here show that Sub1, a transcriptional regulator, acts as an adapter protein, connecting the transcriptome of metabolic genes to the general transcriptional machinery containing Pol II. Despite the fundamental role of Sub1 in PIC formation,^7^ the function of Sub1 is gene-specific and can either positively or negatively affect transcription.^22^ Since Sub1 generally binds to DNA in a sequence-independent manner,^7^ its binding to the promoter of specific genes may be mediated through binding to STFs. We here show that Sub1 interacts with PPARα and ERRα, but not NF-κB, in cardiomyocytes (*Figure 5G*). Sub1 recruits the PIC containing Pol II to the promoters of metabolic genes through its interaction with PPARα and ERRα. Since Sub1 does not interact with NF-κB, the Sub1-mediated Pol II recruitment may be gene-specific. We and others have shown previously that Sirt1 binds to PPARα and ERRα.^5,13,25^ Thus, both Sirt1 and Sub1 may be enriched on the promoters of metabolic genes, and Sirt1 and the components of the PIC compete for binding to Sub1. Although low to modest levels of Sirt1 may not displace the PIC, upregulation of Sirt1 during PO and heart failure appears to be sufficient to displace PIC binding to Sub1. On the other hand, how promoter localization of Sub1 is regulated by Sirt1 in the failing heart remains unknown. In our promoter pull-down assay using the Idh3a promoter, Sirt1 overexpression did not significantly affect DNA binding of Sub1 (*Figure 6E*). However, this *in vitro* assay may not fully recapitulate the chromatin context *in vivo*, possibly because Sirt1-mediated deacetylation is halted due to the absence of NAD in the assay conditions. Given that Sub1 may localize not only to promoter regions but also to gene bodies and transcription termination regions during active transcription,^7^ genome-wide ChIP-seq analysis could provide a more straightforward approach to address this question.

Sirt1 can also play beneficial roles in ageing, fasting, diabetes, and ischaemia. We have shown previously that Sirt1 is upregulated in the aged heart, where it plays a protective role.^12^ Caloric restriction, which reduces the metabolic rate, is known to extend lifespan.^26^ Fasting-induced downregulation of metabolic genes, such as *Sdha,* is normalized in Sirt1^+/−^ mice, indicating that endogenous Sirt1 mediates the metabolic adaptation to nutrient scarcity.^13^ During myocardial ischaemia, excessive FAO and elevated metabolic activity may exacerbate injury, possibly due to increased oxidative stress driven by mitochondrial metabolism.^27^ Whether the salutary actions of Sirt1 are also mediated through regulation of RNA Pol II recruitment remains to be elucidated.

We have previously demonstrated that deacetylation of FoxO by Sirt1 plays a critical role in mediating starvation-induced autophagy in cardiomyocytes.^28^ Given that excessive activation of autophagy can be detrimental to the heart during the acute phase of PO,^29^ it is plausible that Sirt1 may contribute to the progression of heart failure, at least in part, through stimulation of autophagy. However, autophagy activation in response to PO is transient and is rapidly suppressed to below basal levels, largely due to the activation of potent negative regulators such as Mst1,^30^ despite sustained upregulation of Sirt1. Therefore, we propose that autophagy is unlikely to be a major contributor to the deleterious effects of Sirt1 on cardiac function during the chronic phase of PO.

Sirt1 overexpression impaired metabolic capacity under baseline conditions without affecting cardiac function, but exacerbated heart failure under PO, with its detrimental effects more pronounced under increased energy demand. We here show that Sub1-mediated PIC recruitment is competitively inhibited by Sirt1 and, as a result, transcription of metabolic genes is suppressed in the heart during PO. Since Sirt1, an NAD-dependent enzyme, inhibits the binding of Sub1 to TFIIB *in vitro* without NAD (*Figure 7C*), its enzymatic activity may not be essential for the competitive inhibition. Whether this mechanism also minimizes nutrient consumption during caloric restriction and fasting, thereby contributing to longevity and healthy ageing, remains to be clarified.

Both Sirt1 and Sub1 are evolutionarily conserved from prokaryotes. Sir2, a yeast homologue of Sirt1, regulates gene silencing at the ribosomal DNA locus.^31^ Sub1 also localizes to the ribosomal DNA locus.^32^ Both Sirt1 and Sub1 localize to DNA damage sites and mediate DNA repair.^33,34^ Both Sirt1 and Sub1 mediate histone H1 recruitment for heterochromatin formation.^22,35^ In addition to promoting PIC formation at the TSS, Sub1 may also recruit Sirt1 to specific chromatin regions such as the ribosomal DNA locus, DNA damage sites, and heterochromatin. Thus, Sirt1 and Sub1 may share several biological functions. However, Sirt1 is generally thought of as an anti-ageing factor, whereas Sub1 functions as a pro-ageing factor.^36^ Thus, as with the inhibitory effect of Sirt1 on Sub1-induced PIC formation, Sirt1 may counteract Sub1 functions in some contexts.

This study has some limitations. First, the role of the enzymatic activity of Sirt1 in Sirt1–Sub1-mediated transcriptional suppression remains to be fully elucidated. Sirt1 recruited by Sub1 may not only inhibit PIC formation but also deacetylate substrate proteins nearby, such as histones. Testing this hypothesis is challenging, as Sub1 knockdown may reduce histone acetylation indirectly by disrupting the formation of the PIC, which may interact with histone acetyltransferases.^37^ Second, the relation between the effect of Sirt1 upon Pol II recruitment and the previously reported effect of Sirt1 upon transcription factors/co-factors, such as inhibition of PGC-1α^38^ or Sirt1/PPARα heterodimer-driven histone deacetylation,^39^ remain undefined. These mechanisms may operate simultaneously or interdependently. Although we could speculate that the stimulatory effect of Sirt1 upon transcription factors/co-factors could be overridden by the inhibitory effect of Sirt1 upon Pol II recruitment in the presence of the excessive upregulation Sirt1, further investigations are required to clarify these unresolved mechanisms and their contribution to the transcriptional regulation of metabolic genes during PO. Third, the methodologies used in this study have inherent technical limitations. ChIP-seq analyses performed using whole-heart tissue do not permit the assessment of cell type-specific chromatin occupancy. In addition, although the direct interaction between Sirt1 and Sub1 was confirmed by *in vitro* binding assays, other candidate proteins identified by mass spectrometry may interact with Sirt1 indirectly rather than through direct physical association. Thus, additional studies employing complementary approaches, including cell type-specific chromatin profiling and orthogonal methods for validating protein–protein interactions, will be necessary to confirm these molecular interactions and further refine the mechanistic interpretation of our findings.

In summary, we here demonstrate that Sirt1-mediated displacement of the PIC from the Sub1-PPARα/ERRα complex and consequent inhibition of Pol II recruitment contributes to the metabolic disturbance in heart failure. These findings suggest that restoring Pol II recruitment to metabolic gene promoters, through selective disruption of the Sirt1–Sub1 interaction, may offer a more targeted therapeutic approach than global Sirt1 knockdown or inhibition, as it may alleviate metabolic dysfunction while preserving the beneficial effects of Sirt1.

Translational perspective Metabolic remodelling is a hallmark of heart failure, yet existing therapies fail to adequately address the transcriptional mechanisms that drive this process. This study identifies Sirt1 as a negative regulator of metabolic gene expression through inhibition of RNA polymerase II recruitment via the Sub1 complex. Under conditions of PO, Sirt1 disrupts the general transcriptional machinery, unveiling a previously unrecognized maladaptive pathway that contributes to cardiac dysfunction. Therapeutically, targeting the Sirt1–Sub1 interaction could restore metabolic gene transcription and improve myocardial energetics, offering a novel strategy to treat heart failure driven by impaired mitochondrial metabolism.

## Supplementary Material

cvag178_Supplementary_Data

## Data Availability

The data that support the findings of this study are available from the corresponding author upon reasonable request.
