# Supplementary material for: Sirt1 inhibits the recruitment of RNA polymerase II to metabolic gene promoter during pressure overload through competition with the preinitiation complex on Sub1
Source: Cardiovasc Res. 2026 Aug 21;122(15):2116–29. doi: 10.1093/cvr/cvag178 (PMC13742060; doi:10.1093/cvr/cvag178)
Supplement: Supplementary file 1 [file cvag178_supplementary_data.zip › Sub1 Supplemental Material R3 (clean).pdf]

SUPPLEMENTAL MATERIAL

Sirt1 inhibits the recruitment of RNA polymerase II to metabolic gene promoter during pressure overload through competition with the preinitiation complex on Sub1

Short title: ***Sirt1 inhibits Pol II recruitment***

Shin-ichi Oka, Youssef Mourad, Samta Veera, Peiyong Zhai, Ralph Alcendor, Yunping Guo, Jamie Ades, Sanchita Kashyap, Xiaoyong Xu, Chengchen Hu, Toshihide Kashihara, Takanobu Yamamoto, Yoshiyuki Ikeda, Akihiro Shirakabe, Shohei Ikeda, Wataru Mizushima, Yasuki Nakada, Danish Sayed, Zhi Yang, Maha Abdellatif, Junichi Sadoshima.

Department of Cell Biology and Molecular Medicine, Rutgers New Jersey Medical School, Newark, NJ 07103.

Correspondence:
Junichi Sadoshima, MD PhD
Cardiovascular Research Institute,
Rutgers New Jersey Medical School
185 South Orange Ave., MSB G609
Newark, NJ 07103
Tel: (973)972-8619
Fax: (973)972-8919
Email: sadoshju@njms.rutgers.edu

Supplemental Methods

Animals

Cardiac-specific Sirt1 heterozygous knockout mice were generated by crossing αMHC-Cre mice with $Sirt1^{flox/+}$ mice on a C57BL/6 background. Cardiac-specific Sirt1 overexpression mice (Tg-Sirt1, line 40) were generated using the α-myosin heavy chain promoter [1]. In addition, a transgenic line carrying Sirt1 under the control of a tetracycline response element (line 12) on an FVB background exhibited increased Sirt1 expression in the absence of induction and was therefore used without tetracycline treatment. Non-transgenic (NTg) littermates from the same breeder were used as controls.

Transverse aortic constriction (TAC)

This method used to impose PO in mice has been described previously [2]. Mice were randomized and anesthetized with isoflurane inhalation (5% for induction and 1% for maintenance) and mechanically ventilated. The adequacy of anesthesia was assessed by absence of pedal withdrawal reflex throughout the procedure. Analgesia was provided with Ethiqa XR (3.25 mg/kg, subcutaneous injection, once), Meloxicam SR (4 mg/kg, subcutaneous injection, every 24 h for 3 days), and local analgesia along the incision using either Lidocaine (3 mg/kg, once) or Bupivacaine (2.5 mg/kg, once). Adequacy of analgesia was assessed by monitoring the activity and general condition of the animals. The left side of the chest was opened at the second intercostal space. Aortic constriction was performed by ligation of the transverse thoracic aorta between the innominate artery and left common carotid artery using a 7-0 prolene suture tied against a 26- or 27-gauge needle under an operating microscope. The needle was removed, the chest was closed, and the mice were extubated and allowed to recover in a warming unit. After surgery, mice were monitored at least once daily. Sham-

operated mice underwent the same procedure without aortic constriction. At the end of each experiment, animals were euthanized by cervical dislocation under deep isoflurane anesthesia.

Echocardiography

Mice were anesthetized using isoflurane inhalation. Echocardiography was performed using ultrasonography (Acuson Sequoia C256, Siemens Medical Solutions USA Inc., Malvern, PA). A 13-MHz linear ultrasound transducer was used. Two-dimension guided M-mode measurements of left ventricular (LV) internal diameter were taken from at least three beats and averaged. LV end-diastolic diameter (LVEDD) was measured at the time of the apparent maximal LV diastolic dimension, while LV end-systolic diameter (LVESD) was measured at the time of the most anterior systolic excursion of the posterior wall. LV ejection fraction and fractional shortening were calculated as follows: Ejection fraction=$[(LVEDD)^3-(LVESD)^3]/(LVEDD)^3 \times 100$; Fractional shortening=(LVEDD-LVESD)/LVEDD x 100.

Histological analyses

Heart specimens were fixed with 10% formalin and sectioned at 6-μm thickness. Fibrosis was evaluated by picric acid sirius red (PASR) staining. The positively stained fibrotic area was measured with the ImageJ program and normalized by total area. Cell size was evaluated by wheat germ agglutinin (WGA) staining using the ImageJ program. At least 10 images were used for each slide. Apoptosis was evaluated by TUNEL staining. DAPI mounting solution was used to stain the myocyte nuclei. Under 200X magnification, slides were analyzed for positive TUNEL staining in the nucleus. The number of TUNEL positive nuclei number was normalized by total area. Mitochondrial size and aspect ratio were evaluated by electron microscopic images using the ImageJ program.

Fatty acid oxidation

Dissected ventricular tissue was weighed and washed with PBS. The ventricular tissue was homogenized in 400 μl [0.25 M Sucrose, 1 mM EDTA]/100 mg tissue weight. The heart homogenate was dispensed 40 μl per tube and centrifuged at 800g for 1.5 minutes. The precipitates were incubated with 200 μl Reaction buffer [10 mM Hepes pH 7.4, 150 mM KCl, 0.1 mM EDTA, 1 mM K_2HPO_4, 10 mM $MgCl_2$, 1 mM Malate, 10 mM Glucose, and 1 mCi/20 pmol ^{3}H-Oleic acid] at 37 °C for 10 minutes. The reaction was terminated by the addition of 1 ml chloroform/methanol (2:1) and mixed well. The samples were kept at -20 °C for 2 to 24 hours and centrifuged at 12,000g at room temperature for 10 minutes. The supernatant (approximately 400 μl) containing ^3H_2O was mixed with 4 ml scintillation fluid and the radioactivity was measured with a scintillation counter.

Primary cultures of neonatal rat ventricular myocytes

Neonatal rat ventricular cardiomyocytes were used for all in vitro cell culture experiments. Primary myocytes were prepared from 1-day-old Crl: (WI) BR-Wistar rats (Harlan). A cardiac myocyte-rich fraction was obtained by centrifugation through a discontinuous Percoll gradient. Cells were cultured in complete medium for 1 - 2 days. Cells were then treated with reagents or adenovirus for further studies. The complete medium contains Dulbecco's modified Eagle's medium (DMEM)/F-12 supplemented with 5% horse serum, 4 µg/ml transferrin, 0.7 ng/ml sodium selenite (Life Technologies, Inc.), 2 g/l bovine serum albumin (fraction V), 3 mM pyruvic acid, 15 mM Hepes, 100 µM ascorbic acid, 100 µg/ml ampicillin, 5 µg/ml linoleic acid, and 100 µM 5-bromo-2'-deoxyuridine (Sigma).

AC16 cells

AC16 cells derived from human cardiomyocytes were purchased from PromoCell (cat no. C-128010) and maintained according to the manufacturer's instructions.

Reporter gene assay

Luciferase assays were performed in primary cultured rat cardiac myocytes. Reporter plasmids (0.3 μg) and Sirt1 expression vector (pDC316-Sirt1, 0.1 to 0.7 μg) were transfected into cells plated on a 12 well plate using LipofectAmine 2000 (Invitrogen). To avoid a squelching effect, the total expression vector driven by the CMV promoter was kept constant with the control backbone vector. For Sirt1 knockdown experiments, adenovirus vector expressing shSirt1 was transduced into the cells after 16 hours of transfection with reporter plasmids. Luciferase assays were performed 2 to 3 days after transfection and 4 days after adenovirus transduction using a luciferase assay system (Promega).

Plasmid vectors

The mammalian expression and shuttle vector for pDC316-Sirt1 was described previously [3]. The mammalian expression and shuttle vector for pDC316-Flag-Sub1 was generated by insertion of Flag-tagged mouse Sub1 cDNA into pDC316 (Microbix). The bacterial expression vector GST-fused Sirt1 (pCold-GST-Sirt1) was described previously [4]. GST-fused Sirt1 truncate mutants were generated by standard methods using PCR.

The reporter gene constructs for intrinsic promoters were generated by insertion of the promoter regions into pGL3Basic. The promoter regions were amplified by PCR from mouse genome DNA using the following primers (5' to 3'): Ndufs1, CCGGPOCATGGCGAAGGCTGGAACG and CCAGATCTTCCTCCGGAGCCCACCGA; Ndufv1, AAGGGGGGPOCTTTAGTGGGCGAGGCCGAGGACA and GGGGAAAGATCTCGCGGGAGCCAGACGGGTCAC. Underlined sequences represent

endogenous promoter sequences for amplification. Non-underlined sequences are exogenous sequences added to allow enzyme digestion for insertion into pGL3Basic. The reporter gene driven by the Mcad promoter was described previously [5].

Oxygen consumption

Oxygen consumption rate (OCR) was measured with a Seahorse XFe96 Analyzer (Agilent, Santa Clara, CA, USA). Cardiomyocytes were plated at a density of $1X10^5$ cells/well in 96-well Seahorse assay plates. The cells were transduced with adenovirus vectors for shTrx1 and the scrambled control 6 days prior to measurements. The medium was replaced with XF Base medium supplemented with 10 mM glucose and incubated for 1 hour in a 37°C incubator without CO_2. OCR was sequentially measured at baseline, followed by injection with oligomycin (1 μM) to measure ATP-linked OCR. Carbonylcyanide-p-trifluoromethoxyphenylhydrazone (FCCP, 3 μM), an uncoupler, was used to determine maximal respiration, and rotenone (1 μM) was injected to determine the non-mitochondrial respiration.

siRNA for Sub1 and Sirt1

Small interfering RNA (siRNA) for rat Sub1 (S02006466) and control siRNA (1022076) were obtained from Qiagen. siRNA targeting mouse Sirt1 (AM16708) was obtained from Ambion and was used for Sirt1 knockdown experiments, including those performed in AC16 human cardiomyocyte-derived cells. The siRNA was transfected with Lipofectamine RNAiMax (Invitrogen). Forty μl of Lipofectamine RNAiMax was diluted with 750 μl OPTI-MEM. Twenty-two μl of 20 μM siRNA was diluted with 750 μl OPTI-MEM. The diluted Lipofectamine RNAiMax and siRNA was then mixed and incubated at room temperature for 20 minutes. The mixture was added to cardiomyocytes plated on a 10 cm dish with 8.5 ml DMEM/F21 medium. The cells were used for experiments after 24 hours of transfection.

Quantitative real time polymerase chain reaction (qRT-PCR)

Total RNA was extracted from mouse hearts using the RNeasy fibrosis kit (Qiagen) and cDNA was generated using the High Capacity cDNA Reverse Transcription Kit (Applied Bioscience). Gene expression was assessed by qRT-PCR. Ribosomal Protein S15 (RPS15) was used as an internal control. The following primers were used for investigating mouse genes : Cpt2 - GGCCAGGAAGCTGTAAATCATGCAT and TGAGATGTAGCTGGTGTGCTTATTCTG; Mcad - AACACTPOTATGCCTCGATTGCA and CCATAGCCT CCGAAAATCTGAA; Atp5k - CGCCAAGCGCPOAGTPOCTAAAAC and GACGCCTCACTTGAGAATGCTGTC; Idh3a - CTTCGCCACATGGGACTTTTTGACC-3 and CCAGCAGGAGTGCTAATCTAAGTCTTTGA; Sdha - CACCCTCTCATATGTGGACATCAAGACT and GAAGTGACTCCTTGTTCATGAGAGCAG; Sdhd - CTGTGGTGGACPOTCTCTGGCT and CATCGTGGTAATTGAAGTAGCAAAGCCC; Ndufs1 - TGAAAATCCAGGTAGCACAAAGAPOC and ACGTGGCAGTATTTGAAGCAGTTTCT; Ndufv1 - CAGAAGTCCAGCATGCTGAGGT and GCCCCAGATATAAGAGTTTAAGAGTTTTC; and RPS15 - TTCGCAAGTTCACCPOC and GAGAACTCGCCCAGGTAGTGG.

Chromatin immunoprecipitation (ChIP) assay

Heart tissue or cultured cardiomyocytes were cross-linked with formaldehyde. The nuclear fraction was isolated and sonicated to generate a chromatin solution that was then used for immunoprecipitation with Pol II antibody (Millipore 05-623). The following primers were used for investigating the mouse genome: (5' to 3'). Cpt2 - ACAAGGATAGGTCTGCCAAATGCCA and AGTAAGCCCAGCTTCTTGCCCGAA; Mcad - ATCTAGCCCAGAATTTGTTGTTCCAGTG and TCTAGGCCAGAGGGCGCAG; Atp5k - TATCCTCCAGTCGCTAGGGGG and ATTTTGTCCGTGACCTTCGCGC; and Idh3a - TGATGGATGGCGGAGGCGAGC and TGGGGACACCCGGAGCAGPO. The collected chromatin fragments were validated by qPCR with Maxima SYBR Green qPCR master mix (Fermentas).

Purification of bacterially expressed proteins

BL21 *E.coli* strain was transformed with pCold-GST-Sirt1 and its truncate mutants. The *E.coli* was grown in 3 ml LB medium overnight at 37 °C, and then transferred to 250 ml LB medium. After overnight culture at 15 °C, the *E.coli* was lysed in lysis buffer (1% Triton X-100, 1 mM dithiothreitol (DTT), PBS) with sonication. The lysate was incubated with 0.5 ml Glutathione-sepharose 4B (GE Healthcare) for 1 hour at 4 °C. The sepharose was washed 3 times with 1 ml of lysis buffer. The recombinant GST-Sirt1 proteins were stored at -80 °C. Recombinant Sub1 was purchased from SnoBiological (14326-H07E).

LC-MS/MS analysis

Cultured cardiomyocytes (10 cm dish) were infected with either HA-Sirt1 adenovirus or control adenovirus. Cell lysates were prepared using lysis buffer containing 50 mM Tris-HCl (pH 7.6), 1% Triton X-100, 10 mM EDTA, 150 mM NaCl, 50 mM NaF, 100 µM DTT, and protease inhibitor cocktail (Sigma). HA-Sirt1 and associated proteins were immunoprecipitated using anti-HA antibody-conjugated magnetic beads (Thermo Scientific #88836). Control samples underwent the identical immunoprecipitation procedure using lysates prepared from cardiomyocytes infected with control adenovirus. The immunocomplexes were washed three times with 1 ml of the same lysis buffer prior to analysis. The immune complex containing HA-Sirt1 was separated by SDS-PAGE and subjected to in-gel trypsin digestion. The resulting peptides were desalted using a C18 spin Cartridge (Pierce) and further analyzed by LC-MS/MS using an Orbitrap Fusion Lumos mass spectrometer coupled with an Ultimate 3000 Nano UHPLC System (Thermo Fisher Scientific). MS/MS spectra were searched against the UniProt Rat database using Sequest search engine on the Proteome Discoverer (V2.4) platform, with a protein false discovery rate (FDR) of less than 1%. Protein relative quantitation was performed using label

free quantitation (LFQ) method, and proteins enriched in the HA-Sirt1 immunoprecipitate relative to the control immunoprecipitate were identified as candidate Sirt1-associated proteins.

***In vitro* DNA binding assay**

Biotin-labeled Idh3a promoter (380 bp) was prepared by PCR with a biotin-labeled primer as follows: biotin-CGAGAGACCCGAGCTAGGG and TGGGGACACCCGGAGCAGPO. Sirt1 was expressed or knocked down in primary cultured cardiomyocytes using an adenovirus vector. Alternatively, Sub1 was knocked down with siRNA. After 2-3 days of transduction or trasfection, the cells were lysed with 300 μl of a nuclear lysis buffer [25 mM Hepes pH 7.9, 0.1 mM EDTA, 10 mM Sodium Butyrate, 10 mM NaF, 1 mM DTT, 1% NP-40 substitute, 420 mM KCl, 1x Proteinase inhibitor cocktail]. The 100 μl lysate was diluted with 300 μl of a dilution buffer [10 mM Hepes pH 7.9, 2.5 mM $MgCl_2$, 5% Glycerol, 1 mM DTT, 0.1% NP-40]. The total 400 μl of diluted lysate was incubated with 1 μg biotin-labeled DNA and 15 μl streptavidin beads (Thermo Scientific) at 4 °C for 2-4 hours with rotation. The protein-DNA complex was washed 3 times with 1 ml of a washing buffer [10 mM Hepes pH 7.9, 2.5 mM $MgCl_2$, 5% Glycerol, 50 mM KCl, 1 mM DTT, 0.1% NP-40]. The protein bound to the DNA was analyzed by Western blot analyses.

Supplemental Data

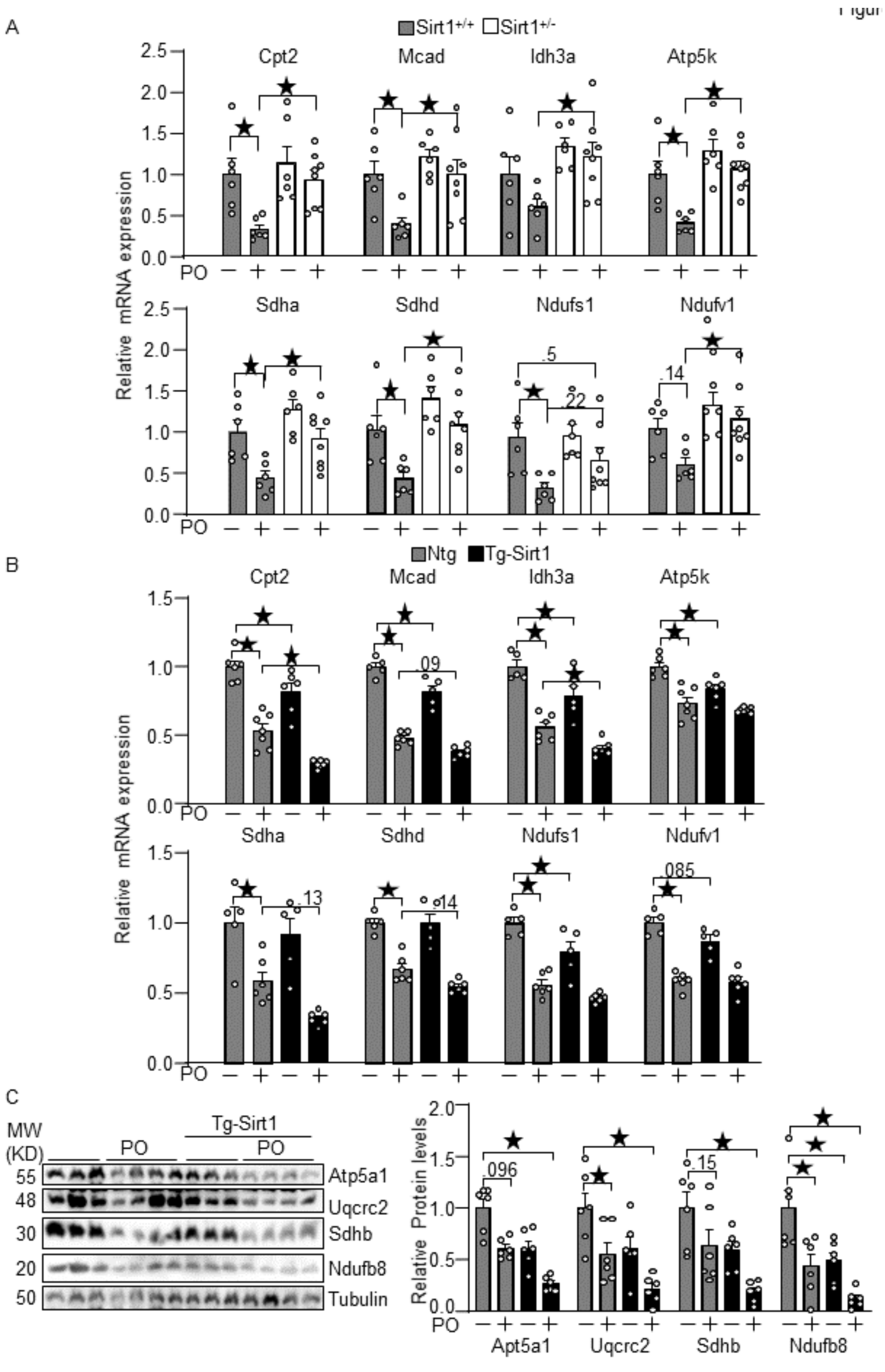

Figure S1. Sirt1 mediates PO-induced suppression of metabolic genes. (A) PO-induced suppression of metabolic genes is normalized in $Sirt1^{+/-}$ mice. (B) Cardiac specific Sirt1 overexpression negatively regulates metabolic gene expression. (C) Sirt1 overexpression suppresses indicated mitochondrial proteins. Sirt1 occupancy at target gene promoters after 4 weeks of PO, assessed by ChIP-qPCR. After 4 weeks of PO, gene expression (A-B) and western blot analyses (C) were performed. The numbers of mice examined in each experimental group were: 6 (A), 5-7 (B) and 6-8 (C). Statistical significance was determined with Kruskal-Wallis test (C (Atp5a1)) and ANOVA for all other comparisons.

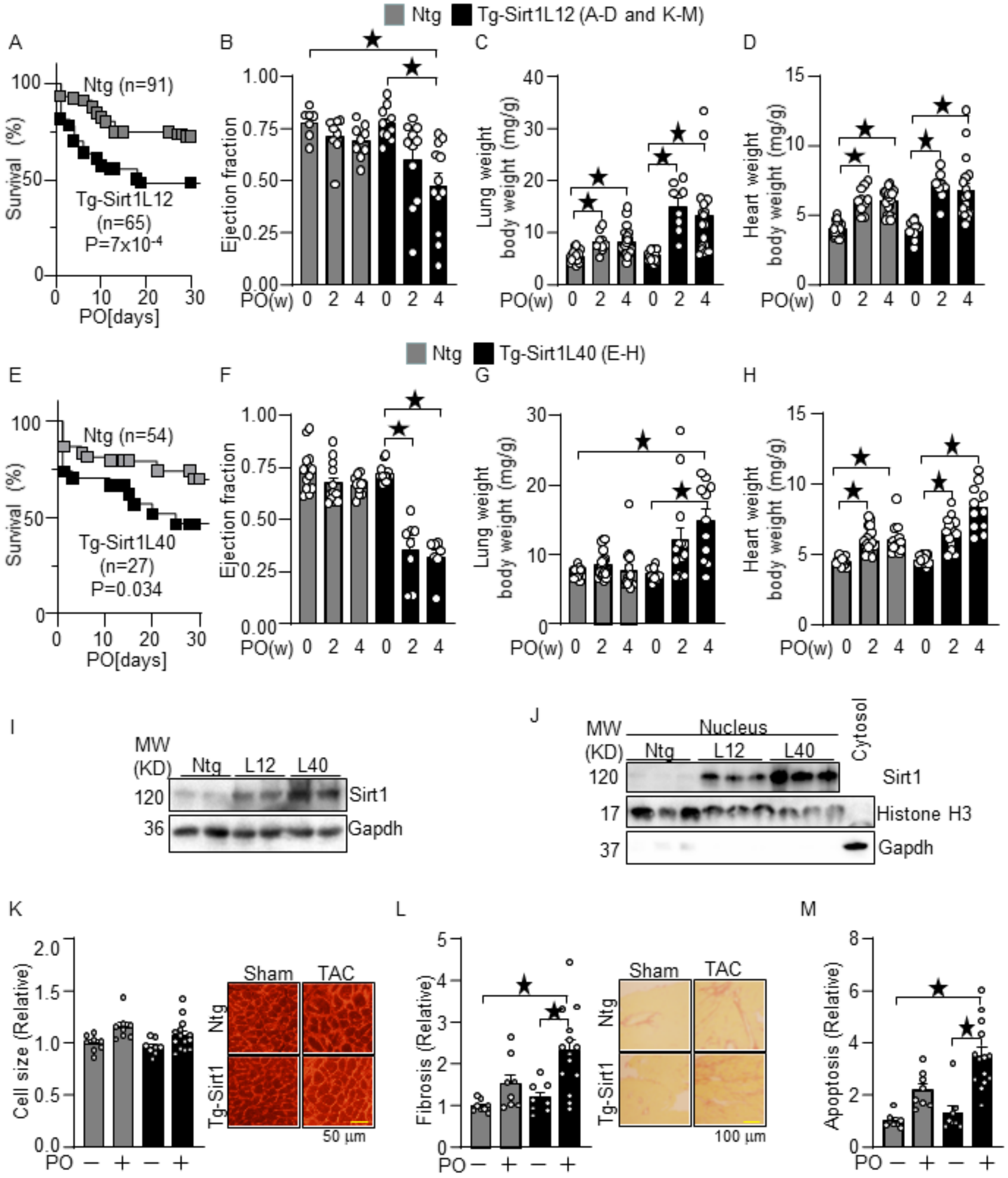

Ntg
Tg-Sirt1L12 (A-D and K-M)
A
B
C
D
Ntg (n=91)
Tg-Sirt1L12
(n=65)
P=7x10-4
Survival (%)
PO[days]
Ejection fraction
PO(w)
Lung weight
body weight (mg/g)
Heart weight
body weight (mg/g)
Ntg
Tg-Sirt1L40 (E-H)
E
F
G
H
Ntg (n=54)
Tg-Sirt1L40
(n=27)
P=0.034
I
MW
(KD)
Ntg
L12
L40
Sirt1
Gapdh
J
Nucleus
Cytosol
Histone H3
K
Cell size (Relative)
PO
Sham
TAC
Tg-Sirt1
50 µm
L
Fibrosis (Relative)
100 µm
M
Apoptosis (Relative)

Figure S2. Cardiac-specific Sirt1 overexpression promotes PO-induced failing heart phenotypes. (A and E) Tg-Sirt1 mouse lines show a higher mortality rate during PO conditions. (B-D and F-H) PO-induced contractile dysfunction (B and F), Lung congestion (C and G) and cardiac hypertrophy (D and H) were exacerbated in Tg-Sirt1 mice. (I-J) Upregulation of Sirt1 in whole heart lysate (I) and nuclear extraction (J) in Tg-Sirt1 mice. (K) Comparative cardiomyocyte hypertrophy in Tg-Sirt1 mice. (L) PO-induced cardiac fibrosis is promoted in Tg-Sirt1 mice. (M) PO-induced apoptotic cell death (tunel positive cells) is increased in Tg-Sirt1 mice. (K-M) FVB genetic background of wild type and Tg-Sirt1 mice were subjected to 4 weeks of PO. The numbers of mice examined in each experimental group were: 34-70 (A), 8-23 (B-C), 10-17 (D), 27-54 (E), 7-14 (F), 11-20 (G-H) and 8-14 (K-M). Statistical significance was determined with Kaplan-Meier (A and E) and Kruskal-Wallis test for all other comparisons.

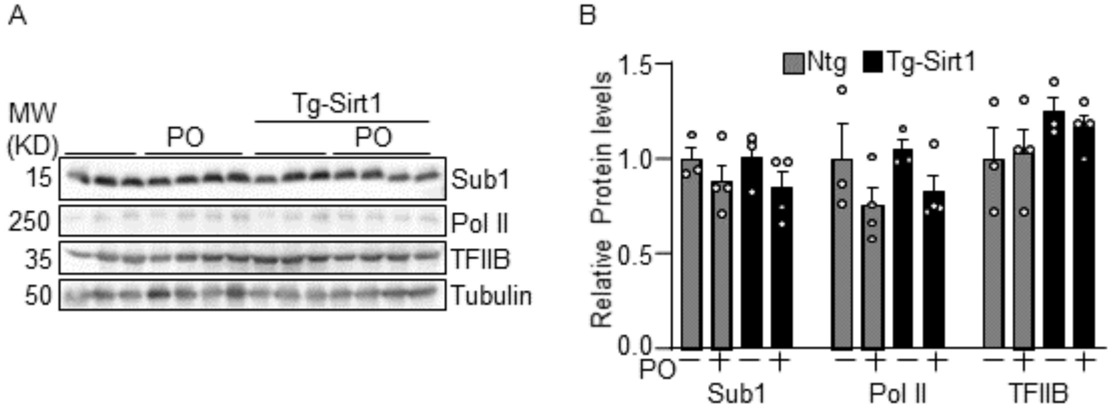

Figure S3. Sirt1 overexpression and PO do not alter Sub1, Pol II, or TFIID expression in the heart (N=3–4).

Supplemental References

1. Alcendor RR, Gao S, Zhai P, Zablocki D, Holle E, Yu X, Tian B, Wagner T, Vatner SF, Sadoshima J. Sirt1 regulates aging and resistance to oxidative stress in the heart. *Circ Res* 2007;**100**:1512-1521.
2. Oka S, Alcendor R, Zhai P, Park JY, Shao D, Cho J, Yamamoto T, Tian B, Sadoshima J. PPARalpha-Sirt1 Complex Mediates Cardiac Hypertrophy and Failure through Suppression of the ERR Transcriptional Pathway. *Cell Metab* 2011;**14**:598-611.
3. Alcendor RR, Kirshenbaum LA, Imai S, Vatner SF, Sadoshima J. Silent information regulator 2alpha, a longevity factor and class III histone deacetylase, is an essential endogenous apoptosis inhibitor in cardiac myocytes. *Circ Res* 2004;**95**:971-980.
4. Oka SI, Zhai P, Yamamoto T, Ikeda Y, Byun J, Hsu CP, Sadoshima J. PPARalpha Association With Sirt1 Suppresses Cardiac Fatty Acid Metabolism in the Failing Heart. *Circ Heart Fail* 2015.
5. Huang CY, Oka SI, Xu X, Chen CF, Tung CY, Chang YY, Mourad Y, Vehra O, Ivessa A, Yehia G, Romanienko P, Hsu CP, Sadoshima J. PERM1 regulates genes involved in fatty acid metabolism in the heart by interacting with PPARalpha and PGC-1alpha. *Sci Rep* 2022;**12**:14576.
