# Supplementary material for: Sirt1 inhibits the recruitment of RNA polymerase II to metabolic gene promoter during pressure overload through competition with the preinitiation complex on Sub1
Source: Cardiovasc Res. 2026 Aug 21;122(15):2116–29. doi: 10.1093/cvr/cvag178 (PMC13742060; doi:10.1093/cvr/cvag178)
Supplement: Supplementary file 1 [file cvag178_supplementary_data.zip › TableS1.docx]

Supplementary Table 1: Comparison of identified proteins and their relative quantitation between Ctrl and IP:HA-Sirt1 (sorted by Ratio)

| Description | Accession | Coverage [%] | # Unique Peptides | # AAs | MW [kDa] | calc. pI | Abundances  (Ctrl) | Abundances (IP:HA‐Sirt1) | Ratio (IP:HASirt1/Ctrl) |
| --- | --- | --- | --- | --- | --- | --- | --- | --- | --- |
| Cullin‐5 OS=Rattus norvegicus OX=10116 G | N Q9JJ31 | 2.95 | 2 | 780 | 90.8 | 7.94 | 0.9 | 199.1 | 200.0 |
| NAD‐dependent protein deacetylase sirtuin | ‐ A0A0G2JZ79 | 17.84 | 6 | 555 | 62 | 4.89 | 0 | 200 | 200.0 |
| Keratin 84 OS=Rattus norvegicus OX=10116 | A0A0G2K0I4 | 8.06 | 2 | 608 | 65.3 | 7.93 | 0 | 200 | 200.0 |
| NADH dehydrogenase [ubiquinone] 1 alpha | Q5BK63 | 5.04 | 1 | 377 | 42.5 | 9.82 | 0 | 200 | 200.0 |
| Cytochrome c oxidase subunit 5B, mitochon | P12075 | 15.50 | 1 | 129 | 13.9 | 7.78 | 0 | 200 | 200.0 |
| Carboxylic ester hydrolase OS=Rattus norve | D4AA05 | 2.66 | 1 | 563 | 62.3 | 5.22 | 0 | 200 | 200.0 |
| Plexin B2 OS=Rattus norvegicus OX=10116 | G D3ZQ57 | 1.25 | 2 | 1841 | 205.8 | 5.99 | 0 | 200 | 200.0 |
| Zinc‐binding alcohol dehydrogenase, domai | D4A264 | 3.98 | 1 | 377 | 40.5 | 7.77 | 0 | 200 | 200.0 |
| DNA polymerase delta‐interacting protein 2 | E9PT51 | 3.26 | 1 | 368 | 41.8 | 8.63 | 0 | 200 | 200.0 |
| Propionyl‐CoA carboxylase beta chain, mito | P07633 | 2.40 | 1 | 541 | 58.6 | 7.47 | 0 | 200 | 200.0 |
| Programmed cell death 6‐interacting protei | nA0A140TAA4 | 2.42 | 2 | 868 | 95.8 | 6.52 | 0 | 200 | 200.0 |
| S‐phase kinase‐associated protein 1 OS=Rat | A0A0G2K4X8 | 7.27 | 1 | 165 | 18.9 | 4.54 | 0 | 200 | 200.0 |
| cAMP‐dependent protein kinase type I‐alph | P09456 | 4.72 | 1 | 381 | 43.1 | 5.35 | 0 | 200 | 200.0 |
| ADP‐ribose glycohydrolase MACROD1 (Frag | Q8K4G6 | 4.26 | 1 | 258 | 28.6 | 8.41 | 0 | 200 | 200.0 |
| Laminin subunit alpha 2 OS=Rattus norvegic | F1M614 | 0.70 | 2 | 3121 | 343.6 | 6.19 | 0 | 200 | 200.0 |
| Non‐specific serine/threonine protein kinas | e Q5I0I1 | 4.54 | 2 | 551 | 62.7 | 6.02 | 0 | 200 | 200.0 |
| Poly [ADP‐ribose] polymerase 1 OS=Rattus n | P27008 | 1.28 | 1 | 1014 | 112.6 | 8.97 | 0 | 200 | 200.0 |
| Leucine‐rich PPR motif‐containing protein, | m F1LM33 | 1.15 | 1 | 1392 | 156.6 | 6.61 | 0 | 200 | 200.0 |
| Uncharacterized protein OS=Rattus norvegi | c F1LTJ5 | 0.45 | 1 | 2449 | 262.9 | 5.99 | 0 | 200 | 200.0 |
| Malonyl‐CoA decarboxylase, mitochondrial | Q920F5 | 2.24 | 1 | 492 | 54.7 | 8.72 | 0 | 200 | 200.0 |
| Flotillin‐2 OS=Rattus norvegicus OX=10116 | G Q9Z2S9 | 3.04 | 1 | 428 | 47 | 5.2 | 0 | 200 | 200.0 |
| Methylcrotonoyl‐CoA carboxylase subunit a | F1LP30 | 1.40 | 1 | 715 | 79.2 | 7.14 | 0 | 200 | 200.0 |

| Kynurenine‐‐oxoglutarate transaminase 3 O | Q58FK9 | 2.64 | 1 | 454 | 51 | 8.35 | 0 | 200 | 200.0 |
| --- | --- | --- | --- | --- | --- | --- | --- | --- | --- |
| Glycogen debrancher OS=Rattus norvegicus | D4AEH9 | 0.65 | 1 | 1532 | 174.2 | 6.74 | 0 | 200 | 200.0 |
| RNA‐splicing ligase RtcB homolog OS=Rattu | s Q6AYT3 | 2.18 | 1 | 505 | 55.2 | 7.23 | 0 | 200 | 200.0 |
| Dehydrogenase/reductase (SDR family) me | m Q6AXY8 | 3.83 | 1 | 313 | 34 | 8.21 | 0 | 200 | 200.0 |
| Lamb2 protein OS=Rattus norvegicus OX=10 | Q5M7W9 | 0.69 | 1 | 1593 | 173.4 | 7.03 | 0 | 200 | 200.0 |
| Protein arginine N‐methyltransferase 1 OS= | Q63009 | 3.12 | 1 | 353 | 40.5 | 5.6 | 0 | 200 | 200.0 |
| Peroxiredoxin‐6 OS=Rattus norvegicus OX=1 | O35244 | 5.36 | 1 | 224 | 24.8 | 5.94 | 0 | 200 | 200.0 |
| Atypical kinase COQ8A, mitochondrial OS=R | Q5BJQ0 | 1.54 | 1 | 649 | 72.2 | 6.46 | 0 | 200 | 200.0 |
| Formin‐like 3 OS=Rattus norvegicus OX=101 | A0A0G2JXC2 | 0.96 | 1 | 934 | 106.9 | 7.28 | 0 | 200 | 200.0 |
| Aspartate‐‐tRNA ligase, cytoplasmic OS=Rat | P15178 | 3.99 | 1 | 501 | 57.1 | 6.44 | 0 | 200 | 200.0 |
| Complex I‐23kD OS=Rattus norvegicus OX=1 | B0BNE6 | 5.19 | 1 | 212 | 24 | 6.21 | 0 | 200 | 200.0 |
| Netrin‐1 OS=Rattus norvegicus OX=10116 G | Q924Z9 | 1.66 | 1 | 604 | 67.8 | 8.79 | 0 | 200 | 200.0 |
| Low molecular weight cytosolic acid phosph | Z4YNF4 | 5.66 | 1 | 159 | 18.2 | 6.32 | 0 | 200 | 200.0 |
| Protein transport protein Sec31A OS=Rattus | Q9Z2Q1 | 1.04 | 1 | 1249 | 135.2 | 7.09 | 0 | 200 | 200.0 |
| Glycerol kinase OS=Rattus norvegicus OX=1 | 0 Q63060 | 2.48 | 1 | 524 | 57.4 | 5.66 | 0 | 200 | 200.0 |
| NF‐kappa‐B‐activating protein OS=Rattus no | A0A0G2K4P5 | 2.41 | 1 | 415 | 47.5 | 10.2 | 0 | 200 | 200.0 |
| Eukaryotic translation initiation factor 4, ga | m F1LN59 | 1.10 | 1 | 906 | 102 | 7.14 | 0 | 200 | 200.0 |
| Myomesin 2 OS=Rattus norvegicus OX=101 | 1 G3V7K1 | 1.78 | 3 | 1464 | 164.6 | 5.88 | 5.4 | 194.6 | 36.0 |
| Cathepsin B OS=Rattus norvegicus OX=1011 | Q6IN22 | 7.67 | 2 | 339 | 37.5 | 5.76 | 7.2 | 192.8 | 26.8 |
| Perilipin OS=Rattus norvegicus OX=10116 G | M0RA08 | 2.51 | 1 | 438 | 47.3 | 5.72 | 7.3 | 192.7 | 26.4 |
| Dynamin‐like 120 kDa protein, mitochondri | a Q2TA68 | 0.94 | 1 | 960 | 111.2 | 7.53 | 10.6 | 189.4 | 17.9 |
| 5‐demethoxyubiquinone hydroxylase, mitoc | A0A0U1RRX6 | 11.22 | 1 | 98 | 10.7 | 9.6 | 10.8 | 189.2 | 17.5 |
| Creatine kinase OS=Rattus norvegicus OX=1 | A0A0G2JVQ1 | 15.35 | 4 | 417 | 47.2 | 9.17 | 13.9 | 186.1 | 13.4 |
| Cytochrome c oxidase subunit 2 OS=Rattus | n P00406 | 11.45 | 2 | 227 | 25.9 | 4.73 | 14.8 | 185.2 | 12.5 |

| Mitochondrial proton/calcium exchanger pr | Q5XIN6 | 1.76 | 1 | 739 | 83 | 6.6 | 14.9 | 185.1 | 12.4 |
| --- | --- | --- | --- | --- | --- | --- | --- | --- | --- |
| von Willebrand factor A domain‐containing | A0A0G2K3W1 | 2.08 | 1 | 481 | 53.7 | 8.76 | 15 | 185 | 12.3 |
| Collagen type VI alpha 1 chain OS=Rattus no | D3ZUL3 | 2.44 | 2 | 1025 | 108.7 | 5.36 | 15.2 | 184.8 | 12.2 |
| Propanoyl‐CoA:carbon dioxide ligase subuni | A0A0G2K401 | 1.94 | 1 | 723 | 79.8 | 7.21 | 15.4 | 184.6 | 12.0 |
| Flotillin‐1 OS=Rattus norvegicus OX=10116 | G Q9Z1E1 | 10.51 | 4 | 428 | 47.5 | 7.15 | 15.7 | 184.3 | 11.7 |
| Nebulin‐related‐anchoring protein OS=Ratt | u D4A4K6 | 2.20 | 3 | 1727 | 196.2 | 9.31 | 16 | 184 | 11.5 |
| Gup1, glycerol uptake/transporter homolog | D4A9P9 | 4.17 | 2 | 503 | 56.3 | 6.6 | 17.2 | 182.8 | 10.6 |
| Cadherin‐13 OS=Rattus norvegicus OX=1011 | Q8R490 | 1.68 | 1 | 714 | 78 | 5.15 | 17.7 | 182.3 | 10.3 |
| Sodium/calcium exchanger 1 OS=Rattus nor | Q01728 | 4.94 | 3 | 971 | 108.1 | 5 | 17.8 | 182.2 | 10.2 |
| ATP synthase F1 subunit alpha OS=Rattus n | oA0A0G2K099 | 30.12 | 1 | 508 | 54.3 | 9.13 | 18 | 182 | 10.1 |
| NADH dehydrogenase [ubiquinone] flavopr | o Q5XIH3 | 7.97 | 3 | 464 | 50.7 | 8.07 | 19.9 | 180.1 | 9.1 |
| Alpha‐1,4 glucan phosphorylase OS=Rattus | G3V6Y6 | 5.08 | 2 | 846 | 96.7 | 6.9 | 19.9 | 180.1 | 9.1 |
| Multifunctional fusion protein OS=Rattus no | A0A0G2JVM0 | 1.42 | 1 | 562 | 61.8 | 7.91 | 20.3 | 179.7 | 8.9 |
| 4‐trimethylaminobutyraldehyde dehydroge | Q9JLJ3 | 9.11 | 4 | 494 | 53.6 | 6.92 | 20.9 | 179.1 | 8.6 |
| Pyruvate dehydrogenase E1 component sub | P49432 | 12.53 | 3 | 359 | 39 | 6.65 | 21 | 179 | 8.5 |
| Complex I‐B18 OS=Rattus norvegicus OX=10 | D3ZLT1 | 13.87 | 1 | 137 | 16.6 | 8.46 | 21.4 | 178.6 | 8.3 |
| Ryanodine receptor 2 OS=Rattus norvegicus | F1LS89 | 0.97 | 4 | 4938 | 560.9 | 6.14 | 21.7 | 178.3 | 8.2 |
| Short‐chain specific acyl‐CoA dehydrogenas | P15651 | 11.65 | 4 | 412 | 44.7 | 8.28 | 21.8 | 178.2 | 8.2 |
| 3‐ketoacyl‐CoA thiolase, mitochondrial OS= | R G3V9U2 | 12.09 | 4 | 397 | 41.9 | 8.15 | 22 | 178 | 8.1 |
| Lon protease homolog, mitochondrial OS=R | Q924S5 | 1.05 | 1 | 950 | 105.7 | 6.6 | 22.3 | 177.7 | 8.0 |
| Succinate dehydrogenase [ubiquinone] flav | o Q920L2 | 14.94 | 7 | 656 | 71.6 | 7.17 | 22.5 | 177.5 | 7.9 |
| Vacuolar protein sorting‐associated protein | G3V8A5 | 1.38 | 1 | 796 | 91.7 | 5.44 | 23.1 | 176.9 | 7.7 |
| Carnitine O‐acetyltransferase OS=Rattus no | Q704S8 | 4.79 | 2 | 626 | 70.8 | 8.54 | 23.4 | 176.6 | 7.5 |
| Cytochrome c oxidase subunit 5A, mitochon | P11240 | 16.44 | 2 | 146 | 16.1 | 6.54 | 24.1 | 175.9 | 7.3 |

| NADH‐ubiquinone oxidoreductase 75 kDa s | u Q66HF1 | 10.73 | 5 | 727 | 79.4 | 5.9 | 24.2 | 175.8 | 7.3 |
| --- | --- | --- | --- | --- | --- | --- | --- | --- | --- |
| Heat shock protein 75 kDa, mitochondrial O | Q5XHZ0 | 5.38 | 2 | 706 | 80.4 | 7.03 | 24.8 | 175.2 | 7.1 |
| Electron transfer flavoprotein subunit beta | O Q68FU3 | 14.90 | 4 | 255 | 27.7 | 7.75 | 24.9 | 175.1 | 7.0 |
| Guanine nucleotide‐binding protein G(i) sub | P04897 | 14.93 | 3 | 355 | 40.5 | 5.45 | 25.6 | 174.4 | 6.8 |
| Dynactin subunit 1 OS=Rattus norvegicus O | X D4A8U7 | 0.94 | 1 | 1281 | 141.9 | 5.9 | 25.6 | 174.4 | 6.8 |
| NADH dehydrogenase [ubiquinone] iron‐sul | Q641Y2 | 8.64 | 4 | 463 | 52.5 | 6.99 | 26.3 | 173.7 | 6.6 |
| Electron transfer flavoprotein‐ubiquinone o | Q66HF3 | 9.42 | 4 | 616 | 68.1 | 7.56 | 26.3 | 173.7 | 6.6 |
| Alpha‐1,4 glucan phosphorylase OS=Rattus | G3V8V3 | 10.21 | 6 | 842 | 97.2 | 7.11 | 26.4 | 173.6 | 6.6 |
| Cytoplasmic dynein 1 heavy chain 1 OS=Rat | t M0R9X8 | 0.90 | 3 | 4644 | 531.7 | 6.42 | 26.4 | 173.6 | 6.6 |
| Tripartite motif‐containing protein 72 OS=R | a A0JPQ4 | 7.76 | 3 | 477 | 52.8 | 6.27 | 26.5 | 173.5 | 6.5 |
| Aspartate aminotransferase, mitochondrial | P00507 | 16.28 | 7 | 430 | 47.3 | 9 | 26.6 | 173.4 | 6.5 |
| Hexokinase‐2 OS=Rattus norvegicus OX=101 | P27881 | 1.20 | 1 | 917 | 102.5 | 6.1 | 26.8 | 173.2 | 6.5 |
| Dystrobrevin OS=Rattus norvegicus OX=101 | D4A772 | 2.24 | 1 | 626 | 70.9 | 8.54 | 26.8 | 173.2 | 6.5 |
| Actin, aortic smooth muscle OS=Rattus norv | P62738 | 87.53 | 1 | 377 | 42 | 5.39 | 26.9 | 173.1 | 6.4 |
| 2‐oxoglutarate dehydrogenase, mitochondr | Q5XI78 | 7.72 | 6 | 1023 | 116.2 | 6.77 | 27.2 | 172.8 | 6.4 |
| Succinyl‐CoA:3‐ketoacid coenzyme A transf | e B2GV06 | 7.31 | 3 | 520 | 56.2 | 8.47 | 27.3 | 172.7 | 6.3 |
| Complex I‐30kD OS=Rattus norvegicus OX=1 | D3ZG43 | 9.85 | 2 | 264 | 30.2 | 7.53 | 27.6 | 172.4 | 6.2 |
| Aconitate hydratase, mitochondrial OS=Ratt | Q9ER34 | 33.21 | 20 | 780 | 85.4 | 7.83 | 27.7 | 172.3 | 6.2 |
| Pcolce protein OS=Rattus norvegicus OX=10 | Q5RJN2 | 6.69 | 2 | 493 | 53.1 | 8.18 | 27.9 | 172.1 | 6.2 |
| AP‐2 complex subunit alpha OS=Rattus norv | D3ZUY8 | 0.92 | 1 | 977 | 107.6 | 7.06 | 28.2 | 171.8 | 6.1 |
| Tissue‐type plasminogen activator‐like OS=R | A0A0G2K1V0 | 3.88 | 1 | 309 | 34.8 | 8.53 | 28.5 | 171.5 | 6.0 |
| Aspartate aminotransferase, cytoplasmic O | S P13221 | 10.41 | 4 | 413 | 46.4 | 7.21 | 28.7 | 171.3 | 6.0 |
| Nucleoside diphosphate kinase B OS=Rattus | P19804 | 25.00 | 3 | 152 | 17.3 | 7.44 | 28.7 | 171.3 | 6.0 |
| Glucose‐6‐phosphate isomerase OS=Rattus | Q6P6V0 | 6.81 | 3 | 558 | 62.8 | 7.77 | 29 | 171 | 5.9 |

| U5 small nuclear ribonucleoprotein 200 kDa | F1LNJ2 | 0.89 | 2 | 2139 | 244.7 | 6.09 | 29.1 | 170.9 | 5.9 |
| --- | --- | --- | --- | --- | --- | --- | --- | --- | --- |
| Phosphoglycerate mutase 2 OS=Rattus norv | P16290 | 7.91 | 2 | 253 | 28.7 | 8.72 | 29.6 | 170.4 | 5.8 |
| Pyruvate dehydrogenase E1 component sub | P26284 | 25.90 | 9 | 390 | 43.2 | 8.19 | 29.7 | 170.3 | 5.7 |
| Medium‐chain specific acyl‐CoA dehydroge | n P08503 | 11.40 | 4 | 421 | 46.5 | 8.41 | 30 | 170 | 5.7 |
| Protein NDRG2 OS=Rattus norvegicus OX=1 | 0A0A0G2JSV0 | 3.77 | 1 | 371 | 40.7 | 5.3 | 30.1 | 169.9 | 5.6 |
| Collagen type VI alpha 2 chain OS=Rattus no | F1LNH3 | 0.97 | 1 | 1027 | 109.6 | 6.61 | 30.1 | 169.9 | 5.6 |
| Serine protease inhibitor A3F‐like OS=Rattu | s F1M8F5 | 3.41 | 1 | 410 | 46.3 | 7.8 | 31 | 169 | 5.5 |
| Dolichyl‐diphosphooligosaccharide‐‐protein | P07153 | 7.77 | 4 | 605 | 68.3 | 6.51 | 31.7 | 168.3 | 5.3 |
| Acyl‐Coenzyme A dehydrogenase, very long | Q5M9H2 | 12.21 | 5 | 655 | 70.8 | 8.81 | 31.8 | 168.2 | 5.3 |
| Omega‐amidase NIT2 OS=Rattus norvegicus | Q497B0 | 5.43 | 1 | 276 | 30.7 | 7.3 | 32.3 | 167.7 | 5.2 |
| Eukaryotic translation initiation factor 2 sub | Q6P685 | 5.11 | 2 | 333 | 38.2 | 5.8 | 32.3 | 167.7 | 5.2 |
| Transcriptional activator protein Pur‐beta O | Q68A21 | 5.40 | 1 | 315 | 33.4 | 5.43 | 32.6 | 167.4 | 5.1 |
| Mitochondrial carnitine/acylcarnitine carrie | P97521 | 10.96 | 3 | 301 | 33.1 | 9.48 | 32.7 | 167.3 | 5.1 |
| Ras‐related protein Rap‐1b OS=Rattus norve | Q62636 | 5.43 | 1 | 184 | 20.8 | 5.78 | 33.1 | 166.9 | 5.0 |
| Fumarate hydratase, mitochondrial OS=Ratt | P14408 | 8.68 | 3 | 507 | 54.4 | 8.73 | 33.2 | 166.8 | 5.0 |
| Dolichyl‐diphosphooligosaccharide‐‐protein | P61805 | 9.73 | 1 | 113 | 12.5 | 7.08 | 33.9 | 166.1 | 4.9 |
| NADH dehydrogenase [ubiquinone] 1 alpha | F1LZC5 | 6.99 | 2 | 143 | 16.8 | 9.79 | 34.2 | 165.8 | 4.8 |
| ATP synthase membrane subunit K, mitocho | Q9JJW3 | 44.83 | 2 | 58 | 6.4 | 9.83 | 34.6 | 165.4 | 4.8 |
| Myosin regulatory light chain 2, ventricular/ | P08733 | 44.58 | 6 | 166 | 18.9 | 4.93 | 35 | 165 | 4.7 |
| Creatine kinase OS=Rattus norvegicus OX=1 | A0A0G2JSP8 | 21.52 | 7 | 381 | 43 | 7.06 | 35.2 | 164.8 | 4.7 |
| Solute carrier family 25 member 12 OS=Ratt | A0A0G2K2J7 | 10.70 | 2 | 383 | 41.5 | 9.55 | 35.9 | 164.1 | 4.6 |
| Keratin 36 OS=Rattus norvegicus OX=10116 | Q6IFV5 | 7.91 | 2 | 468 | 52.2 | 5.05 | 36.6 | 163.4 | 4.5 |
| NADH dehydrogenase [ubiquinone] flavopr | o P19234 | 4.44 | 1 | 248 | 27.4 | 6.68 | 36.9 | 163.1 | 4.4 |
| AFG3‐like matrix AAA peptidase subunit 2 O | F1LN92 | 3.62 | 3 | 802 | 89.3 | 8.6 | 37 | 163 | 4.4 |

| Annexin OS=Rattus norvegicus OX=10116 G | Q6IMZ3 | 9.36 | 6 | 673 | 75.7 | 5.57 | 37.4 | 162.6 | 4.3 |
| --- | --- | --- | --- | --- | --- | --- | --- | --- | --- |
| Small nuclear ribonucleoprotein Sm D2 OS= | M0R8K2 | 16.95 | 2 | 118 | 13.5 | 9.99 | 37.6 | 162.4 | 4.3 |
| ATP‐dependent 6‐phosphofructokinase OS= | Q52KS1 | 3.08 | 1 | 780 | 85.3 | 7.88 | 37.6 | 162.4 | 4.3 |
| Isocitrate dehydrogenase [NAD] subunit alp | Q99NA5 | 6.01 | 2 | 366 | 39.6 | 6.92 | 37.7 | 162.3 | 4.3 |
| Acyl‐coenzyme A thioesterase 2, mitochond | O55171 | 7.28 | 3 | 453 | 49.7 | 7.83 | 37.8 | 162.2 | 4.3 |
| Long‐chain‐fatty‐acid‐‐CoA ligase 1 OS=Ratt | P18163 | 7.58 | 4 | 699 | 78.1 | 6.99 | 37.9 | 162.1 | 4.3 |
| Succinate‐‐CoA ligase [ADP‐forming] subuni | t F1LM47 | 14.62 | 7 | 465 | 50.3 | 7.69 | 38.8 | 161.2 | 4.2 |
| Sodium/potassium‐transporting ATPase sub | P06685 | 15.93 | 6 | 1023 | 113 | 5.45 | 39.8 | 160.2 | 4.0 |
| Pyruvate kinase PKM OS=Rattus norvegicus | P11980 | 18.64 | 2 | 531 | 57.8 | 7.06 | 40 | 160 | 4.0 |
| Eukaryotic translation initiation factor 3 sub | A0A0G2JU77 | 5.12 | 1 | 215 | 24.8 | 4.97 | 40.2 | 159.8 | 4.0 |
| Phospholemman OS=Rattus norvegicus OX= | O08589 | 13.04 | 1 | 92 | 10.4 | 8.54 | 40.3 | 159.7 | 4.0 |
| Cullin‐associated NEDD8‐dissociated protei | n P97536 | 0.89 | 1 | 1230 | 136.3 | 5.78 | 40.6 | 159.4 | 3.9 |
| Solute carrier family 25 member 13 OS=Ratt | F1LZW6 | 8.47 | 2 | 484 | 54.1 | 7.25 | 40.7 | 159.3 | 3.9 |
| Guanine nucleotide‐binding protein G(o) su | P59215 | 13.84 | 3 | 354 | 40 | 5.53 | 40.8 | 159.2 | 3.9 |
| Rho GDP‐dissociation inhibitor 1 OS=Rattus | Q5XI73 | 7.84 | 1 | 204 | 23.4 | 5.2 | 41 | 159 | 3.9 |
| Ubiquitin‐like modifier‐activating enzyme 1 | Q5U300 | 3.31 | 3 | 1058 | 117.7 | 5.57 | 41.4 | 158.6 | 3.8 |
| Sarcalumenin OS=Rattus norvegicus OX=10 | 1 F1LWG8 | 8.63 | 6 | 904 | 98.3 | 4.44 | 41.5 | 158.5 | 3.8 |
| Phosphoglucomutase‐1 OS=Rattus norvegic | P38652 | 8.90 | 4 | 562 | 61.4 | 6.76 | 41.5 | 158.5 | 3.8 |
| Coronin OS=Rattus norvegicus OX=10116 G | N G3V624 | 4.64 | 2 | 474 | 53.1 | 7.08 | 41.5 | 158.5 | 3.8 |
| EH‐domain‐containing 4 OS=Rattus norvegi | c Q8R3Z7 | 12.57 | 6 | 541 | 61.4 | 6.76 | 42.3 | 157.7 | 3.7 |
| ATP synthase subunit f, mitochondrial OS=R | D3ZAF6 | 23.86 | 2 | 88 | 10.4 | 10.04 | 42.3 | 157.7 | 3.7 |
| Protein phosphatase 2 (Formerly 2A), regula | Q5XI34 | 9.51 | 5 | 589 | 65.3 | 5.11 | 42.9 | 157.1 | 3.7 |
| Guanine nucleotide‐binding protein G(I)/G( | S P54311 | 2.94 | 1 | 340 | 37.4 | 6 | 43.3 | 156.7 | 3.6 |
| Elongation factor Tu, mitochondrial OS=Rat | t P85834 | 32.96 | 11 | 452 | 49.5 | 7.56 | 43.8 | 156.2 | 3.6 |

| Chaperonin containing Tcp1, subunit 6A (Ze | Q3MHS9 | 9.79 | 4 | 531 | 58 | 7.08 | 44.2 | 155.8 | 3.5 |
| --- | --- | --- | --- | --- | --- | --- | --- | --- | --- |
| Cytochrome b‐c1 complex subunit 1, mitoch | Q68FY0 | 17.08 | 6 | 480 | 52.8 | 5.88 | 44.3 | 155.7 | 3.5 |
| Fibroblast growth factor 18 OS=Rattus norv | e O88182 | 20.29 | 3 | 207 | 23.9 | 9.83 | 44.3 | 155.7 | 3.5 |
| 26S proteasome regulatory subunit 8 OS=Ra | P62198 | 8.62 | 3 | 406 | 45.6 | 7.55 | 44.6 | 155.4 | 3.5 |
| Collagen‐binding protein OS=Rattus norvegi | Q5RJR9 | 6.47 | 2 | 417 | 46.5 | 8.82 | 44.8 | 155.2 | 3.5 |
| 26S proteasome non‐ATPase regulatory sub | Q4FZT9 | 2.97 | 2 | 908 | 100.1 | 5.2 | 45.2 | 154.8 | 3.4 |
| RCG45649, isoform CRA_a OS=Rattus norve | A0A0G2QC59 | 9.28 | 3 | 377 | 41.9 | 8.32 | 45.3 | 154.7 | 3.4 |
| Phosphate carrier protein, mitochondrial OS | P16036 | 11.80 | 4 | 356 | 39.4 | 9.33 | 45.5 | 154.5 | 3.4 |
| Hydroxysteroid dehydrogenase‐like protein | Q4V8F9 | 2.48 | 1 | 524 | 58.3 | 6.19 | 45.8 | 154.2 | 3.4 |
| Uncharacterized protein OS=Rattus norvegi | c F1M7L9 | 13.76 | 10 | 792 | 88.5 | 9.03 | 45.9 | 154.1 | 3.4 |
| Zinc finger protein 512 OS=Rattus norvegicu | D3ZIF0 | 2.67 | 1 | 562 | 63.9 | 9.63 | 45.9 | 154.1 | 3.4 |
| T‐complex protein 1 subunit alpha OS=Rattu | P28480 | 9.35 | 4 | 556 | 60.3 | 6.21 | 46 | 154 | 3.3 |
| Myadm protein OS=Rattus norvegicus OX=1 | Q05BA4 | 8.49 | 2 | 318 | 35.1 | 8.31 | 46 | 154 | 3.3 |
| ATP synthase F(0) complex subunit B1, mito | P19511 | 12.50 | 3 | 256 | 28.8 | 9.36 | 46.3 | 153.7 | 3.3 |
| Fibrinogen beta chain OS=Rattus norvegicus | P14480 | 1.88 | 1 | 479 | 54.2 | 7.78 | 46.4 | 153.6 | 3.3 |
| Treacle ribosome biogenesis factor 1 OS=Ra | D4A206 | 4.66 | 6 | 1395 | 144.6 | 9.1 | 46.6 | 153.4 | 3.3 |
| Transitional endoplasmic reticulum ATPase | P46462 | 6.58 | 4 | 806 | 89.3 | 5.26 | 46.6 | 153.4 | 3.3 |
| RAS‐related 2 OS=Rattus norvegicus OX=10 | 1 Q5BJU0 | 13.73 | 2 | 204 | 23.4 | 6.01 | 46.6 | 153.4 | 3.3 |
| WD repeat‐containing protein 1 OS=Rattus | n Q5RKI0 | 10.56 | 4 | 606 | 66.1 | 6.62 | 46.7 | 153.3 | 3.3 |
| Synaptopodin OS=Rattus norvegicus OX=10 | A0A0H2UHQ9 | 3.43 | 2 | 963 | 103.1 | 9.26 | 46.7 | 153.3 | 3.3 |
| ATPase family AAA domain‐containing prote | Q3KRE0 | 5.08 | 3 | 591 | 66.7 | 9.29 | 46.8 | 153.2 | 3.3 |
| Transmembrane protein 33 OS=Rattus norv | Q9Z142 | 4.86 | 1 | 247 | 28 | 9.66 | 46.8 | 153.2 | 3.3 |
| Oxysterol‐binding protein OS=Rattus norveg | D3ZHZ3 | 3.16 | 2 | 886 | 100.5 | 7.08 | 46.9 | 153.1 | 3.3 |
| Lamin‐B1 OS=Rattus norvegicus OX=10116 | G G3V7U4 | 5.96 | 2 | 587 | 66.6 | 5.16 | 47 | 153 | 3.3 |

| 26S proteasome non‐ATPase regulatory sub | F1LMZ8 | 4.74 | 2 | 422 | 47.4 | 6.48 | 47.2 | 152.8 | 3.2 |
| --- | --- | --- | --- | --- | --- | --- | --- | --- | --- |
| Unconventional myosin‐6 OS=Rattus norveg | D4A5I9 | 16.03 | 15 | 1285 | 148 | 8.41 | 47.3 | 152.7 | 3.2 |
| Myl6 protein OS=Rattus norvegicus OX=101 | B2GV99 | 73.68 | 10 | 152 | 17 | 4.65 | 47.6 | 152.4 | 3.2 |
| Sodium/potassium‐transporting ATPase sub | P07340 | 8.22 | 2 | 304 | 35.2 | 8.65 | 48.1 | 151.9 | 3.2 |
| Cardiac troponin C OS=Rattus norvegicus O | X Q4PP99 | 15.53 | 3 | 161 | 18.4 | 4.18 | 48.5 | 151.5 | 3.1 |
| Mitochondrial 2‐oxoglutarate/malate carrie | G3V6H5 | 11.15 | 3 | 314 | 34.1 | 9.96 | 48.8 | 151.2 | 3.1 |
| Coatomer subunit beta' OS=Rattus norvegic | O35142 | 5.19 | 4 | 905 | 102.5 | 5.27 | 48.9 | 151.1 | 3.1 |
| Myosin‐binding protein C, cardiac‐type OS= | P56741 | 21.59 | 20 | 1274 | 140.7 | 6.54 | 49.3 | 150.7 | 3.1 |
| Tubulin alpha‐8 chain OS=Rattus norvegicus | Q6AY56 | 30.51 | 1 | 449 | 50 | 5.1 | 49.5 | 150.5 | 3.0 |
| CCHC‐type zinc finger nucleic acid binding p | P62634 | 4.52 | 1 | 177 | 19.4 | 7.71 | 49.5 | 150.5 | 3.0 |
| Tropomyosin 1, alpha, isoform CRA_p OS=R | A0A0G2JSQ4 | 48.39 | 2 | 248 | 28.5 | 4.77 | 49.6 | 150.4 | 3.0 |
| Protein disulfide‐isomerase OS=Rattus norv | eA0A0H2UHM5 | 12.16 | 6 | 510 | 57 | 6.21 | 49.6 | 150.4 | 3.0 |
| LUC7‐like OS=Rattus norvegicus OX=10116 | G G3V9R0 | 10.78 | 2 | 371 | 44 | 9.94 | 49.6 | 150.4 | 3.0 |
| Supervillin OS=Rattus norvegicus OX=10116 | F1M155 | 28.55 | 46 | 2165 | 241.9 | 6.98 | 49.8 | 150.2 | 3.0 |
| Clathrin heavy chain 1 OS=Rattus norvegicu | s P11442 | 8.36 | 10 | 1675 | 191.5 | 5.72 | 49.9 | 150.1 | 3.0 |
| Four and a half LIM domains protein 2 OS=R | O35115 | 22.58 | 5 | 279 | 32.1 | 7.3 | 49.9 | 150.1 | 3.0 |
| Aminopeptidase OS=Rattus norvegicus OX= | F1M9V7 | 2.39 | 2 | 920 | 103.3 | 5.77 | 50 | 150 | 3.0 |
| Sarcoplasmic/endoplasmic reticulum calciu | m P11507 | 28.09 | 24 | 1043 | 114.7 | 5.34 | 50.1 | 149.9 | 3.0 |
| Troponin I, cardiac muscle OS=Rattus norve | g P23693 | 39.34 | 8 | 211 | 24.1 | 9.55 | 50.2 | 149.8 | 3.0 |
| Leucine‐rich repeat flightless‐interacting pr | o Q66HF9 | 9.62 | 5 | 738 | 80 | 4.78 | 50.4 | 149.6 | 3.0 |
| Uncharacterized protein OS=Rattus norvegi | c D4A8Y1 | 5.80 | 3 | 655 | 71 | 9.41 | 50.4 | 149.6 | 3.0 |
| Spectrin alpha chain, non‐erythrocytic 1 OS | =A0A0G2K1Y8 | 25.98 | 1 | 2498 | 287.5 | 5.35 | 50.5 | 149.5 | 3.0 |
| Unconventional myosin‐Id OS=Rattus norve | Q63357 | 14.91 | 13 | 1006 | 116 | 9.41 | 50.5 | 149.5 | 3.0 |
| Nestin OS=Rattus norvegicus OX=10116 GN | = D3ZWJ2 | 2.93 | 3 | 1502 | 164 | 4.21 | 50.6 | 149.4 | 3.0 |

| 40S ribosomal protein S12 OS=Rattus norve | Q6PDW1 | 24.24 | 3 | 132 | 14.5 | 7.21 | 50.7 | 149.3 | 2.9 |
| --- | --- | --- | --- | --- | --- | --- | --- | --- | --- |
| 60S ribosomal protein L36a OS=Rattus norv | e P83883 | 21.70 | 2 | 106 | 12.4 | 10.58 | 50.8 | 149.2 | 2.9 |
| 60S ribosomal protein L13a OS=Rattus norv | e Q5RK10 | 11.33 | 2 | 203 | 23.4 | 11.02 | 51 | 149 | 2.9 |
| Unconventional myosin‐Ie OS=Rattus norve | g Q63356 | 17.89 | 18 | 1107 | 126.7 | 9.07 | 51.1 | 148.9 | 2.9 |
| LUC7‐like 2 (S. cerevisiae) OS=Rattus norveg | B2RYP6 | 18.88 | 5 | 392 | 46.6 | 10.1 | 51.3 | 148.7 | 2.9 |
| Dihydrolipoyllysine‐residue succinyltransfer | Q01205 | 9.91 | 4 | 454 | 48.9 | 8.69 | 51.3 | 148.7 | 2.9 |
| ATPase Get3 OS=Rattus norvegicus OX=101 | G3V9T7 | 2.87 | 1 | 348 | 38.8 | 4.91 | 51.3 | 148.7 | 2.9 |
| Zinc finger protein 593 OS=Rattus norvegicu | B2GV37 | 19.40 | 2 | 134 | 15.3 | 9.85 | 51.8 | 148.2 | 2.9 |
| Midkine OS=Rattus norvegicus OX=10116 G | Q9R1S9 | 26.43 | 3 | 140 | 15.4 | 9.7 | 51.9 | 148.1 | 2.9 |
| Heat shock protein beta‐2 OS=Rattus norve | g O35878 | 6.04 | 1 | 182 | 20.3 | 5.43 | 52 | 148 | 2.8 |
| Leiomodin‐2 OS=Rattus norvegicus OX=101 | 1 A1A5Q0 | 4.19 | 2 | 549 | 61.7 | 5.82 | 52.1 | 147.9 | 2.8 |
| Cytochrome b‐c1 complex subunit 8 OS=Rat | Q7TQ16 | 18.29 | 1 | 82 | 9.8 | 10.52 | 52.1 | 147.9 | 2.8 |
| LIM domain 7 OS=Rattus norvegicus OX=10 | 1A0A0G2K174 | 30.04 | 33 | 1395 | 156.7 | 6.21 | 52.2 | 147.8 | 2.8 |
| Coronin‐6 OS=Rattus norvegicus OX=10116 | Q920J3 | 2.97 | 1 | 472 | 52.9 | 6.05 | 52.5 | 147.5 | 2.8 |
| RB‐binding protein 6, ubiquitin ligase OS=Ra | G3V953 | 7.27 | 9 | 1789 | 199.7 | 9.63 | 52.6 | 147.4 | 2.8 |
| Trifunctional enzyme subunit beta, mitocho | Q60587 | 8.00 | 4 | 475 | 51.4 | 9.47 | 52.6 | 147.4 | 2.8 |
| Clusterin OS=Rattus norvegicus OX=10116 G | G3V836 | 8.05 | 3 | 447 | 51.4 | 5.81 | 52.6 | 147.4 | 2.8 |
| 2,4‐dienoyl‐CoA reductase [(3E)‐enoyl‐CoA‐ | Q64591 | 2.99 | 1 | 335 | 36.1 | 8.94 | 52.6 | 147.4 | 2.8 |
| Cleavage and polyadenylation specificity fac | Q5FVR7 | 11.93 | 2 | 243 | 27.4 | 8.22 | 52.8 | 147.2 | 2.8 |
| 60S ribosomal protein L23 OS=Rattus norve | g P62832 | 62.14 | 10 | 140 | 14.9 | 10.51 | 52.9 | 147.1 | 2.8 |
| Isocitrate dehydrogenase [NADP], mitochon | P56574 | 40.71 | 17 | 452 | 50.9 | 8.69 | 53 | 147 | 2.8 |
| Calmodulin‐3 OS=Rattus norvegicus OX=101 | P0DP31 | 28.19 | 3 | 149 | 16.8 | 4.22 | 53.1 | 146.9 | 2.8 |
| 26S proteasome regulatory subunit 4 OS=Ra | P62193 | 2.73 | 1 | 440 | 49.2 | 6.21 | 53.2 | 146.8 | 2.8 |
| LIM domain‐binding 3 OS=Rattus norvegicu | sA0A096MJ01 | 16.12 | 9 | 726 | 76.8 | 7.75 | 53.3 | 146.7 | 2.8 |

| Superoxide dismutase [Mn], mitochondrial | O P07895 | 6.31 | 1 | 222 | 24.7 | 8.81 | 53.4 | 146.6 | 2.7 |
| --- | --- | --- | --- | --- | --- | --- | --- | --- | --- |
| Unconventional myosin‐Ib OS=Rattus norve | Q05096 | 35.21 | 33 | 1136 | 131.8 | 9.36 | 53.5 | 146.5 | 2.7 |
| T‐complex protein 1 subunit eta OS=Rattus | D4AC23 | 2.21 | 1 | 544 | 59.6 | 8 | 53.6 | 146.4 | 2.7 |
| Heat shock protein HSP 90‐alpha OS=Rattus | P82995 | 19.37 | 3 | 733 | 84.8 | 5.01 | 53.8 | 146.2 | 2.7 |
| ATP synthase subunit beta, mitochondrial O | P10719 | 53.69 | 21 | 529 | 56.3 | 5.34 | 53.9 | 146.1 | 2.7 |
| 40S ribosomal protein S5 OS=Rattus norveg | B0BN81 | 18.63 | 3 | 204 | 22.9 | 9.72 | 53.9 | 146.1 | 2.7 |
| Natriuretic peptides A OS=Rattus norvegicu | s P01161 | 8.55 | 1 | 152 | 16.5 | 7.3 | 54.1 | 145.9 | 2.7 |
| Metavinculin OS=Rattus norvegicus OX=101 | A0A0G2K8V2 | 12.61 | 11 | 1134 | 123.5 | 5.71 | 54.5 | 145.5 | 2.7 |
| Protein LTV1 homolog OS=Rattus norvegicu | Q68FR7 | 7.87 | 3 | 470 | 53.5 | 4.98 | 54.6 | 145.4 | 2.7 |
| Catenin alpha 1 OS=Rattus norvegicus OX=1 | A0A0G2JYF7 | 7.04 | 4 | 810 | 89.3 | 5.47 | 54.6 | 145.4 | 2.7 |
| Band 4.1‐like protein 4B OS=Rattus norvegic | A0A0G2K3H4 | 5.37 | 3 | 745 | 83.3 | 9.06 | 54.9 | 145.1 | 2.6 |
| Gap junction alpha‐1 protein OS=Rattus nor | P08050 | 10.73 | 2 | 382 | 43 | 8.76 | 55 | 145 | 2.6 |
| Pescadillo homolog OS=Rattus norvegicus O | Q3B8N8 | 2.90 | 2 | 586 | 67.6 | 7.2 | 55 | 145 | 2.6 |
| Laminin subunit beta 3 OS=Rattus norvegicu | F1LPI5 | 6.14 | 6 | 1172 | 129 | 7.28 | 55.1 | 144.9 | 2.6 |
| 40S ribosomal protein S17 OS=Rattus norve | D3ZFA8 | 42.22 | 5 | 135 | 15.5 | 9.77 | 55.1 | 144.9 | 2.6 |
| Destrin OS=Rattus norvegicus OX=10116 GN | Q7M0E3 | 14.55 | 2 | 165 | 18.5 | 8.03 | 55.2 | 144.8 | 2.6 |
| 14‐3‐3 protein eta OS=Rattus norvegicus OX | P68511 | 8.94 | 1 | 246 | 28.2 | 4.89 | 55.5 | 144.5 | 2.6 |
| ATP synthase subunit alpha OS=Rattus norv | F1LP05 | 38.16 | 6 | 553 | 59.8 | 9.26 | 55.7 | 144.3 | 2.6 |
| Heat shock 70 kDa protein 1A OS=Rattus no | P0DMW0 | 10.30 | 3 | 641 | 70.1 | 5.82 | 55.7 | 144.3 | 2.6 |
| RNA helicase OS=Rattus norvegicus OX=101 | A0A0G2JTM6 | 9.39 | 6 | 703 | 78.7 | 8.31 | 55.8 | 144.2 | 2.6 |
| Elongation factor 1‐gamma OS=Rattus norv | e Q68FR6 | 11.21 | 4 | 437 | 50 | 6.74 | 55.8 | 144.2 | 2.6 |
| 60S acidic ribosomal protein P2 OS=Rattus n | P02401 | 40.00 | 2 | 115 | 11.7 | 4.54 | 55.8 | 144.2 | 2.6 |
| Ankycorbin OS=Rattus norvegicus OX=1011 | 6 Q5U312 | 17.89 | 13 | 978 | 109.1 | 6.15 | 56.1 | 143.9 | 2.6 |
| PRP4 pre‐mRNA‐processing factor 4 homolo | A0A0G2K249 | 8.33 | 5 | 888 | 103.1 | 9.98 | 56.4 | 143.6 | 2.5 |

| RCG55135, isoform CRA_b OS=Rattus norve | G3V852 | 8.42 | 17 | 2541 | 269.5 | 6.2 | 56.5 | 143.5 | 2.5 |
| --- | --- | --- | --- | --- | --- | --- | --- | --- | --- |
| Cofilin 2 OS=Rattus norvegicus OX=10116 G | M0RC65 | 12.65 | 2 | 166 | 18.7 | 7.88 | 56.5 | 143.5 | 2.5 |
| Carnitine O‐palmitoyltransferase 1, muscle i | Q63704 | 8.42 | 5 | 772 | 88.2 | 8.6 | 56.6 | 143.4 | 2.5 |
| Myosin‐6 OS=Rattus norvegicus OX=10116 | G G3V885 | 62.23 | 39 | 1938 | 223.2 | 5.73 | 56.7 | 143.3 | 2.5 |
| Protein phosphatase 1 regulatory subunit 1 | 2 Q10728 | 16.18 | 15 | 1032 | 115.2 | 5.47 | 56.7 | 143.3 | 2.5 |
| Heat shock protein HSP 90‐beta OS=Rattus | n P34058 | 27.21 | 5 | 724 | 83.2 | 5.03 | 56.8 | 143.2 | 2.5 |
| 40S ribosomal protein S28 OS=Rattus norve | P62859 | 46.38 | 3 | 69 | 7.8 | 10.7 | 56.9 | 143.1 | 2.5 |
| Medium‐chain acyl‐CoA ligase ACSF2, mitoc | Q499N5 | 5.85 | 3 | 615 | 67.8 | 8.12 | 56.9 | 143.1 | 2.5 |
| Dystrophin OS=Rattus norvegicus OX=10116 | P11530 | 7.13 | 21 | 3677 | 425.6 | 5.91 | 57 | 143 | 2.5 |
| Activated RNA polymerase II transcriptional | Q63396 | 8.66 | 1 | 127 | 14.4 | 9.6 | 57.4 | 142.6 | 2.5 |
| ATP synthase F1 subunit delta OS=Rattus no | G3V7Y3 | 8.33 | 1 | 168 | 17.6 | 5.24 | 57.7 | 142.3 | 2.5 |
| Ig‐like domain‐containing protein OS=Rattu | sA0A0G2K6T8 | 18.75 | 1 | 96 | 10.3 | 7.12 | 57.9 | 142.1 | 2.5 |
| Peroxidase OS=Rattus norvegicus OX=10116 | A0A0G2JWB6 | 10.98 | 11 | 1475 | 165.3 | 6.99 | 58.1 | 141.9 | 2.4 |
| RCG41580, isoform CRA_a OS=Rattus norve | B2RZD5 | 9.84 | 1 | 122 | 14.5 | 9.45 | 58.2 | 141.8 | 2.4 |
| Collagen alpha‐1(V) chain OS=Rattus norveg | Q9JI03 | 0.60 | 1 | 1840 | 183.9 | 4.97 | 58.2 | 141.8 | 2.4 |
| Fructose‐bisphosphate aldolase A OS=Rattu | P05065 | 29.95 | 9 | 364 | 39.3 | 8.09 | 58.3 | 141.7 | 2.4 |
| Cytospin‐A OS=Rattus norvegicus OX=10116 | Q2KN99 | 6.08 | 5 | 1118 | 124.3 | 5.8 | 58.4 | 141.6 | 2.4 |
| Band 4.1 OS=Rattus norvegicus OX=10116 G | D4A6Q4 | 1.70 | 1 | 766 | 85.3 | 5.39 | 58.4 | 141.6 | 2.4 |
| Glutathione synthetase OS=Rattus norvegic | P46413 | 4.64 | 2 | 474 | 52.3 | 5.67 | 58.5 | 141.5 | 2.4 |
| Scribble planar cell polarity protein OS=Ratt | D3ZWS0 | 0.60 | 1 | 1663 | 179.7 | 5.17 | 58.5 | 141.5 | 2.4 |
| F‐actin‐capping protein subunit beta OS=Ra | t Q5XI32 | 33.82 | 8 | 272 | 30.6 | 6 | 58.6 | 141.4 | 2.4 |
| RING‐type E3 ubiquitin transferase OS=Ratt | D4ACF2 | 38.85 | 17 | 471 | 54.1 | 6.7 | 58.7 | 141.3 | 2.4 |
| Protein chibby homolog 2 OS=Rattus norveg | Q6AXV6 | 2.46 | 1 | 447 | 52 | 6.89 | 58.7 | 141.3 | 2.4 |
| EF‐hand domain‐containing protein D2 OS= | R Q4FZY0 | 36.40 | 7 | 239 | 26.7 | 5.07 | 58.8 | 141.2 | 2.4 |

| Unconventional myosin‐Ic OS=Rattus norve | g | Q63355 | 41.76 | 36 | 1044 | 119.7 | 9.39 | 59 | 141 | 2.4 |
| --- | --- | --- | --- | --- | --- | --- | --- | --- | --- | --- |
| Myosin regulatory light chain RLC‐A OS=Rat | t | P13832 | 62.79 | 6 | 172 | 19.9 | 4.81 | 59.2 | 140.8 | 2.4 |
| Protein phosphatase 1 regulatory subunit O |  | D3ZIC4 | 20.44 | 13 | 993 | 111.3 | 5.62 | 59.4 | 140.6 | 2.4 |
| Pre‐mRNA 3'‐end‐processing factor FIP1 OS | = | Q5U317 | 11.75 | 4 | 536 | 60.2 | 5.57 | 59.4 | 140.6 | 2.4 |
| T‐complex protein 1 subunit gamma OS=Rat |  | Q6P502 | 6.24 | 3 | 545 | 60.6 | 6.64 | 59.4 | 140.6 | 2.4 |
| T‐complex protein 1 subunit beta OS=Rattu | s | Q5XIM9 | 3.74 | 2 | 535 | 57.4 | 6.46 | 59.6 | 140.4 | 2.4 |
| Protein‐glutamine gamma‐glutamyltransfer |  | Q9WVJ6 | 22.01 | 11 | 686 | 76.9 | 5.1 | 59.9 | 140.1 | 2.3 |
| RNA helicase OS=Rattus norvegicus OX=101 |  | B2RYL8 | 21.70 | 10 | 622 | 69.8 | 6.8 | 59.9 | 140.1 | 2.3 |
| L‐lactate dehydrogenase B chain OS=Rattus |  | P42123 | 20.66 | 5 | 334 | 36.6 | 6.05 | 60 | 140 | 2.3 |
| Similar to PS1D protein (Predicted) OS=Ratt |  | D3ZCQ7 | 9.54 | 2 | 241 | 27.5 | 9.63 | 60 | 140 | 2.3 |
| Microtubule‐associated protein 4 OS=Rattu | s | Q5M7W5 | 2.46 | 2 | 1057 | 110.2 | 4.73 | 60 | 140 | 2.3 |
| 60 kDa heat shock protein, mitochondrial O |  | P63039 | 28.45 | 12 | 573 | 60.9 | 6.18 | 60.1 | 139.9 | 2.3 |
| Nucleolar protein 16 OS=Rattus norvegicus | O | Q1RP77 | 24.16 | 4 | 178 | 21.1 | 9.91 | 60.1 | 139.9 | 2.3 |
| 28S ribosomal protein S7, mitochondrial OS |  | Q5I0K8 | 4.96 | 1 | 242 | 28.2 | 10.02 | 60.1 | 139.9 | 2.3 |
| Non‐specific serine/threonine protein kinas | eA0A0G2K8N1 | | 1.92 | 1 | 8031 | 874.2 | 6.01 | 60.1 | 139.9 | 2.3 |
| 60S ribosomal protein L26 OS=Rattus norve | g P12749 | | 17.24 | 3 | 145 | 17.3 | 10.62 | 60.2 | 139.8 | 2.3 |
| Myosin phosphatase Rho‐interacting protei | nA0A140TA95 | | 27.90 | 19 | 993 | 113 | 6.16 | 60.3 | 139.7 | 2.3 |
| Y‐box‐binding protein 1 OS=Rattus norvegic | Q3ZAV2 | | 18.10 | 3 | 326 | 36.2 | 9.88 | 60.4 | 139.6 | 2.3 |
| Annexin A5 OS=Rattus norvegicus OX=1011 | 6 P14668 | | 17.24 | 5 | 319 | 35.7 | 5.05 | 60.4 | 139.6 | 2.3 |
| Actin‐related protein 2/3 complex subunit 5 | Q4KLF8 | | 31.13 | 2 | 151 | 16.3 | 5.67 | 60.4 | 139.6 | 2.3 |
| Integrin beta OS=Rattus norvegicus OX=101 | A0A0G2JSK5 | | 2.13 | 1 | 798 | 88.3 | 6.01 | 60.5 | 139.5 | 2.3 |
| Histone H1.1 OS=Rattus norvegicus OX=101 | D4A3K5 | | 25.70 | 3 | 214 | 22 | 10.99 | 60.7 | 139.3 | 2.3 |
| Vitronectin OS=Rattus norvegicus OX=1011 | 6 Q3KR94 | | 4.81 | 2 | 478 | 54.7 | 6.05 | 60.8 | 139.2 | 2.3 |
| Actin‐related protein 2/3 complex subunit 5 | A1L108 | | 16.34 | 2 | 153 | 17 | 6.8 | 60.9 | 139.1 | 2.3 |

| Protein‐glutamine gamma‐glutamyltransfer | P23606 | 2.55 | 2 | 824 | 90.7 | 6.47 | 60.9 | 139.1 | 2.3 |
| --- | --- | --- | --- | --- | --- | --- | --- | --- | --- |
| Similar to 60S ribosomal protein L23a OS=R | a F1LT35 | 31.41 | 4 | 156 | 17.7 | 10.46 | 61 | 139 | 2.3 |
| KN motif and ankyrin repeat domain‐contai | n D4ACC2 | 4.52 | 3 | 840 | 90.2 | 5.47 | 61 | 139 | 2.3 |
| PRKC apoptosis WT1 regulator protein OS=R | Q62627 | 10.54 | 2 | 332 | 35.8 | 5.74 | 61 | 139 | 2.3 |
| Tropomyosin alpha‐1 chain OS=Rattus norv | eA0A0G2K7F7 | 57.76 | 8 | 303 | 34.6 | 4.81 | 61.1 | 138.9 | 2.3 |
| Myosin heavy chain 7B OS=Rattus norvegicu | B6RK61 | 4.12 | 1 | 1941 | 221.5 | 5.91 | 61.1 | 138.9 | 2.3 |
| Trifunctional enzyme subunit alpha, mitoch | o Q64428 | 16.64 | 9 | 763 | 82.6 | 9.06 | 61.1 | 138.9 | 2.3 |
| Heat shock cognate 71 kDa protein OS=Ratt | P63018 | 31.73 | 14 | 646 | 70.8 | 5.52 | 61.2 | 138.8 | 2.3 |
| 60S ribosomal protein L34 OS=Rattus norve | g B2RZD4 | 7.69 | 1 | 117 | 13.3 | 11.47 | 61.2 | 138.8 | 2.3 |
| Tropomyosin alpha‐3 chain OS=Rattus norv | e Q63610 | 44.76 | 5 | 248 | 29 | 4.78 | 61.3 | 138.7 | 2.3 |
| Laminin subunit gamma 2 OS=Rattus norveg | F1LRH4 | 5.96 | 5 | 1192 | 130.8 | 6.21 | 61.5 | 138.5 | 2.3 |
| 60S ribosomal protein L39 OS=Rattus norve | g P62893 | 19.61 | 1 | 51 | 6.4 | 12.56 | 61.5 | 138.5 | 2.3 |
| Troponin T, cardiac muscle OS=Rattus norve | P50753 | 42.81 | 15 | 299 | 35.7 | 4.98 | 61.7 | 138.3 | 2.2 |
| 40S ribosomal protein S2 OS=Rattus norveg | D3ZIY7 | 29.74 | 7 | 269 | 29.5 | 9.28 | 61.7 | 138.3 | 2.2 |
| 40S ribosomal protein S30 OS=Rattus norve | Q5BJN7 | 9.02 | 3 | 133 | 14.4 | 10.17 | 61.7 | 138.3 | 2.2 |
| 40S ribosomal protein S26 OS=Rattus norve | A0A0G2JY64 | 20.87 | 2 | 115 | 12.9 | 10.11 | 61.7 | 138.3 | 2.2 |
| Plasminogen activator inhibitor 1 RNA‐bindi | Q6AXS5 | 3.93 | 1 | 407 | 44.7 | 8.54 | 61.7 | 138.3 | 2.2 |
| Guanine nucleotide‐binding protein‐like 3 O | Q811S9 | 49.07 | 22 | 538 | 60.6 | 9.14 | 61.8 | 138.2 | 2.2 |
| RNA binding motif protein 39 OS=Rattus no | r Q5BJP4 | 9.92 | 4 | 524 | 58.6 | 10.13 | 61.8 | 138.2 | 2.2 |
| Malate dehydrogenase, cytoplasmic OS=Rat | O88989 | 15.27 | 4 | 334 | 36.5 | 6.58 | 61.8 | 138.2 | 2.2 |
| Profilin‐1 OS=Rattus norvegicus OX=10116 | G P62963 | 32.14 | 3 | 140 | 14.9 | 8.28 | 62 | 138 | 2.2 |
| LLP homolog, long‐term synaptic facilitation | B0BN98 | 22.22 | 2 | 126 | 14.9 | 10.24 | 62 | 138 | 2.2 |
| Phosphoinositide phospholipase C OS=Rattu | D4A978 | 23.35 | 13 | 788 | 89.4 | 6.93 | 62.2 | 137.8 | 2.2 |
| Succinate‐‐CoA ligase [GDP‐forming] subuni | F1LPV8 | 5.10 | 2 | 431 | 46.6 | 7.71 | 62.2 | 137.8 | 2.2 |

| Myosin light chain 4 OS=Rattus norvegicus | O | P17209 | 51.30 | 9 | 193 | 21.3 | 5.03 | 62.3 | 137.7 | 2.2 |
| --- | --- | --- | --- | --- | --- | --- | --- | --- | --- | --- |
| Nexilin OS=Rattus norvegicus OX=10116 GN |  | Q9Z2J4 | 33.54 | 2 | 656 | 78.3 | 5.36 | 62.4 | 137.6 | 2.2 |
| FLII, actin‐remodeling protein OS=Rattus no |  | Q5RKI5 | 11.02 | 13 | 1270 | 144.8 | 6 | 62.4 | 137.6 | 2.2 |
| Multiple myeloma tumor‐associated protein |  | Q5M9I6 | 22.69 | 5 | 260 | 29.3 | 9.85 | 62.5 | 137.5 | 2.2 |
| Mitochondrial 18 kDa protein OS=Rattus no |  | Q5XIG9 | 7.83 | 1 | 166 | 18.4 | 9.45 | 62.5 | 137.5 | 2.2 |
| Alpha‐centractin OS=Rattus norvegicus OX= |  | P85515 | 2.66 | 1 | 376 | 42.6 | 6.64 | 62.6 | 137.4 | 2.2 |
| Neural cell adhesion molecule 1 OS=Rattus | n | F1LUV9 | 2.64 | 2 | 833 | 91.6 | 4.91 | 62.7 | 137.3 | 2.2 |
| 60S ribosomal protein L18 OS=Rattus norve | g | P12001 | 10.11 | 2 | 188 | 21.6 | 11.78 | 62.7 | 137.3 | 2.2 |
| MAP7 domain‐containing 1 OS=Rattus norv | e | D4A644 | 1.71 | 1 | 817 | 90.4 | 9.98 | 62.7 | 137.3 | 2.2 |
| Fragile X mental retardation syndrome‐relat |  | Q5XI81 | 16.55 | 6 | 568 | 63.9 | 6.39 | 62.8 | 137.2 | 2.2 |
| Zinc finger protein 91 OS=Rattus norvegicus |  | F1MAJ8 | 10.56 | 5 | 568 | 63.2 | 7.77 | 62.8 | 137.2 | 2.2 |
| Hexokinase OS=Rattus norvegicus OX=1011 |  | M0RAQ6 | 6.16 | 5 | 893 | 99.5 | 7.14 | 62.8 | 137.2 | 2.2 |
| Similar to RIKEN cDNA 1500041N16, isoform |  | G3V6J9 | 9.21 | 2 | 315 | 34.4 | 6.05 | 62.8 | 137.2 | 2.2 |
| 40S ribosomal protein S6 OS=Rattus norveg |  | P62755 | 40.56 | 13 | 249 | 28.7 | 10.84 | 62.9 | 137.1 | 2.2 |
| Keratin, type II cytoskeletal 8 OS=Rattus nor |  | Q10758 | 12.84 | 1 | 483 | 54 | 6 | 63.2 | 136.8 | 2.2 |
| Filamin A OS=Rattus norvegicus OX=10116 | G | C0JPT7 | 4.74 | 6 | 2639 | 280.3 | 6.04 | 63.2 | 136.8 | 2.2 |
| ADP‐ribosylation factor 3 OS=Rattus norveg |  | P61206 | 19.89 | 3 | 181 | 20.6 | 7.43 | 63.2 | 136.8 | 2.2 |
| 40S ribosomal protein S4 OS=Rattus norveg |  | X1WI37 | 35.11 | 3 | 262 | 29.4 | 10.15 | 63.3 | 136.7 | 2.2 |
| RCG21137 OS=Rattus norvegicus OX=10116 |  | G3V9N1 | 7.29 | 2 | 288 | 32 | 8.68 | 63.3 | 136.7 | 2.2 |
| Tropomyosin alpha‐4 chain OS=Rattus norv | e | P09495 | 43.95 | 4 | 248 | 28.5 | 4.69 | 63.4 | 136.6 | 2.2 |
| Adiponectin a OS=Rattus norvegicus OX=10 | 1 | G3V7N9 | 14.62 | 3 | 253 | 26.6 | 8.82 | 63.5 | 136.5 | 2.1 |
| Phosphatidylethanolamine‐binding protein |  | P31044 | 14.44 | 2 | 187 | 20.8 | 5.8 | 63.5 | 136.5 | 2.1 |
| Isocitrate dehydrogenase [NAD] subunit bet |  | Q68FX0 | 14.29 | 5 | 385 | 42.3 | 8.75 | 63.6 | 136.4 | 2.1 |
| Myoglobin OS=Rattus norvegicus OX=10116 |  | Q9QZ76 | 9.74 | 1 | 154 | 17.1 | 8.1 | 63.6 | 136.4 | 2.1 |

| ELAV‐like protein 1 OS=Rattus norvegicus O | X B5DF91 | 3.37 | 1 | 326 | 36.1 | 9.04 | 63.6 | 136.4 | 2.1 |
| --- | --- | --- | --- | --- | --- | --- | --- | --- | --- |
| Stress‐70 protein, mitochondrial OS=Rattus | P48721 | 27.39 | 14 | 679 | 73.8 | 6.3 | 63.7 | 136.3 | 2.1 |
| Cofilin‐1 OS=Rattus norvegicus OX=10116 G | P45592 | 7.83 | 1 | 166 | 18.5 | 8.09 | 63.7 | 136.3 | 2.1 |
| Spectrin beta chain OS=Rattus norvegicus O | G3V6S0 | 28.10 | 53 | 2356 | 273.1 | 5.73 | 63.8 | 136.2 | 2.1 |
| RNA helicase OS=Rattus norvegicus OX=101 | A0A0G2K719 | 15.56 | 7 | 662 | 73.1 | 7.18 | 63.8 | 136.2 | 2.1 |
| Xin actin‐binding repeat‐containing protein | F1LMC2 | 7.36 | 17 | 3302 | 373.8 | 6.04 | 63.9 | 136.1 | 2.1 |
| 40S ribosomal protein S27‐like OS=Rattus n | o P24051 | 15.48 | 1 | 84 | 9.5 | 9.45 | 64 | 136 | 2.1 |
| FMR1 autosomal homolog 2 OS=Rattus nor | v B1H2A6 | 3.26 | 2 | 675 | 74.3 | 6.1 | 64.1 | 135.9 | 2.1 |
| Plectin OS=Rattus norvegicus OX=10116 GN | A0A0G2K1J5 | 16.41 | 59 | 4387 | 498.9 | 5.66 | 64.7 | 135.3 | 2.1 |
| Coiled‐coil domain‐containing 191 (Fragmen | A0A1W2Q6A6 | 1.34 | 1 | 597 | 68.8 | 9.19 | 64.7 | 135.3 | 2.1 |
| Aldo‐keto reductase family 1 member B1 O | S P07943 | 13.61 | 4 | 316 | 35.8 | 6.71 | 64.8 | 135.2 | 2.1 |
| Protein Wnt‐5a OS=Rattus norvegicus OX=1 | Q9QXQ7 | 2.89 | 1 | 380 | 42.3 | 8.46 | 64.8 | 135.2 | 2.1 |
| Spectrin alpha chain, non‐erythrocytic 1 OS | = Q6IRK8 | 26.67 | 1 | 2452 | 282.2 | 5.31 | 65 | 135 | 2.1 |
| Unconventional myosin‐Va OS=Rattus norve | A0A0G2K4Y7 | 8.70 | 12 | 1828 | 212 | 8.59 | 65 | 135 | 2.1 |
| Peroxisomal multifunctional enzyme type 2 | P97852 | 3.13 | 2 | 735 | 79.4 | 8.57 | 65 | 135 | 2.1 |
| Transcription elongation factor, mitochondr | A0A0G2JYK4 | 5.03 | 2 | 338 | 38.4 | 9.63 | 65 | 135 | 2.1 |
| 60S ribosomal protein L35 OS=Rattus norve | g P17078 | 30.08 | 5 | 123 | 14.5 | 11.05 | 65.1 | 134.9 | 2.1 |
| WD repeat domain 33 OS=Rattus norvegicu | s F1LT09 | 2.18 | 2 | 1331 | 145.3 | 9.13 | 65.1 | 134.9 | 2.1 |
| Dihydrolipoyllysine‐residue acetyltransferas | P08461 | 2.22 | 1 | 632 | 67.1 | 8.53 | 65.2 | 134.8 | 2.1 |
| Protein‐L‐isoaspartate (D‐aspartate) O‐meth | D3ZY20 | 8.36 | 2 | 359 | 40.7 | 6.7 | 65.5 | 134.5 | 2.1 |
| Prohibitin OS=Rattus norvegicus OX=10116 | A0A0G2KB63 | 26.26 | 7 | 297 | 33.1 | 9.83 | 65.6 | 134.4 | 2.0 |
| Complement C1s subcomponent OS=Rattus | D4A1S0 | 2.06 | 1 | 679 | 76 | 4.93 | 65.6 | 134.4 | 2.0 |
| Cortactin, isoform CRA_c OS=Rattus norveg | i D3ZGE6 | 18.86 | 9 | 546 | 61 | 5.31 | 65.8 | 134.2 | 2.0 |
| 40S ribosomal protein S18 OS=Rattus norve | D3ZM33 | 39.47 | 8 | 152 | 17.8 | 10.99 | 65.8 | 134.2 | 2.0 |

| 3‐hydroxyacyl‐CoA dehydrogenase type‐2 O | O70351 | 19.54 | 4 | 261 | 27.2 | 8.78 | 65.8 | 134.2 | 2.0 |
| --- | --- | --- | --- | --- | --- | --- | --- | --- | --- |
| 60S ribosomal protein L17 OS=Rattus norve | g P24049 | 39.67 | 7 | 184 | 21.4 | 10.18 | 65.9 | 134.1 | 2.0 |
| Nebulette OS=Rattus norvegicus OX=10116 | A0A0G2JWS2 | 6.51 | 5 | 952 | 108.9 | 7.34 | 65.9 | 134.1 | 2.0 |
| Actin‐related protein 2 OS=Rattus norvegicu | Q5M7U6 | 27.66 | 8 | 394 | 44.7 | 6.74 | 66.1 | 133.9 | 2.0 |
| Alpha‐crystallin B chain OS=Rattus norvegic | u P23928 | 44.00 | 6 | 175 | 20.1 | 7.33 | 66.1 | 133.9 | 2.0 |
| Ras homolog family member T1 OS=Rattus | nA0A0G2K777 | 1.33 | 1 | 752 | 86 | 6.79 | 66.1 | 133.9 | 2.0 |
| Acetyl‐CoA acetyltransferase, mitochondria | P17764 | 19.81 | 7 | 424 | 44.7 | 8.76 | 66.3 | 133.7 | 2.0 |
| Alpha‐actinin‐4 OS=Rattus norvegicus OX=1 | A0A0G2K013 | 40.70 | 16 | 892 | 102.9 | 5.5 | 66.6 | 133.4 | 2.0 |
| Superoxide dismutase [Cu‐Zn] OS=Rattus no | P07632 | 15.58 | 2 | 154 | 15.9 | 6.35 | 66.8 | 133.2 | 2.0 |
| 40S ribosomal protein S14 OS=Rattus norve | Q6PDV6 | 31.79 | 6 | 151 | 16.3 | 10.05 | 66.9 | 133.1 | 2.0 |
| 40S ribosomal protein S20 OS=Rattus norve | A0A0G2JY70 | 21.43 | 3 | 112 | 12.6 | 10.15 | 67 | 133 | 2.0 |
| 39S ribosomal protein L15, mitochondrial O | A0A0G2K9B4 | 3.05 | 1 | 295 | 33.6 | 10.1 | 67 | 133 | 2.0 |
| 60 kDa poly(U)‐binding‐splicing factor OS=R | A0A0H2UHZ6 | 13.50 | 5 | 563 | 60.1 | 5.29 | 67.1 | 132.9 | 2.0 |
| 3‐beta‐hydroxysterol Delta (14)‐reductase O | A0A0G2JU83 | 2.58 | 1 | 620 | 70.6 | 9.47 | 67.3 | 132.7 | 2.0 |
| PDZ and LIM domain protein 5 OS=Rattus n | o Q62920 | 19.80 | 7 | 591 | 63.2 | 8.38 | 67.5 | 132.5 | 2.0 |
| Putative 60S ribosomal protein L37a OS=Rat | P61515 | 29.35 | 2 | 92 | 10.3 | 10.43 | 67.6 | 132.4 | 2.0 |
| Actin‐related protein 2/3 complex subunit 3 | B2GV73 | 14.04 | 2 | 178 | 20.5 | 8.6 | 67.7 | 132.3 | 2.0 |
| Filamin‐C OS=Rattus norvegicus OX=10116 | G D3ZHA0 | 21.90 | 40 | 2726 | 290.8 | 5.9 | 67.8 | 132.2 | 1.9 |
| Receptor of activated protein C kinase 1 OS | = P63245 | 14.83 | 4 | 317 | 35.1 | 7.69 | 67.8 | 132.2 | 1.9 |
| Nexilin OS=Rattus norvegicus OX=10116 GN | A0A0G2K6I3 | 34.93 | 1 | 670 | 79.8 | 5.27 | 67.9 | 132.1 | 1.9 |
| Serine and arginine repetitive matrix 1 OS=R | B2RYB3 | 14.76 | 8 | 908 | 102.4 | 11.85 | 67.9 | 132.1 | 1.9 |
| Mitochondrial import inner membrane tran | D3ZJX5 | 3.12 | 1 | 353 | 39.8 | 8.13 | 67.9 | 132.1 | 1.9 |
| CCT‐theta OS=Rattus norvegicus OX=10116 | D4ACB8 | 12.77 | 6 | 548 | 59.6 | 5.54 | 68.1 | 131.9 | 1.9 |
| Glyceraldehyde‐3‐phosphate dehydrogenas | P04797 | 48.35 | 10 | 333 | 35.8 | 8.03 | 68.2 | 131.8 | 1.9 |

| Leucine‐rich repeat flightless‐interacting pr | o Q4V7E8 | 21.28 | 8 | 437 | 49.7 | 5.95 | 68.2 | 131.8 | 1.9 |
| --- | --- | --- | --- | --- | --- | --- | --- | --- | --- |
| Prohibitin 1 OS=Rattus norvegicus OX=1011 | P67779 | 23.53 | 6 | 272 | 29.8 | 5.76 | 68.2 | 131.8 | 1.9 |
| RCG60540, isoform CRA_a OS=Rattus norve | Q3KR55 | 5.44 | 1 | 239 | 27.8 | 8.81 | 68.2 | 131.8 | 1.9 |
| Creatine kinase B‐type OS=Rattus norvegicu | P07335 | 7.09 | 2 | 381 | 42.7 | 5.67 | 68.3 | 131.7 | 1.9 |
| BUD23, rRNA methyltransferase and riboso | m B1H275 | 5.69 | 1 | 281 | 31.5 | 8.88 | 68.3 | 131.7 | 1.9 |
| Tropomyosin beta chain OS=Rattus norvegi | c P58775 | 42.96 | 5 | 284 | 32.8 | 4.7 | 68.4 | 131.6 | 1.9 |
| EWS RNA‐binding protein 1 OS=Rattus norv | eA0A140UHY3 | 5.75 | 2 | 661 | 68.9 | 9.29 | 68.4 | 131.6 | 1.9 |
| 40S ribosomal protein S3 OS=Rattus norveg | P62909 | 52.67 | 11 | 243 | 26.7 | 9.66 | 68.6 | 131.4 | 1.9 |
| 40S ribosomal protein S16 OS=Rattus norve | P62250 | 45.21 | 10 | 146 | 16.4 | 10.21 | 68.7 | 131.3 | 1.9 |
| Transcription intermediary factor 1‐beta OS | O08629 | 4.43 | 2 | 835 | 88.9 | 5.77 | 68.7 | 131.3 | 1.9 |
| RNA‐binding motif protein 25‐like 1 OS=Rat | t D3Z8R4 | 4.04 | 2 | 817 | 96.5 | 6.39 | 68.8 | 131.2 | 1.9 |
| Histone H1.0 OS=Rattus norvegicus OX=101 | P43278 | 23.71 | 4 | 194 | 20.9 | 10.9 | 69.2 | 130.8 | 1.9 |
| Sorting and assembly machinery componen | Q6AXV4 | 8.74 | 4 | 469 | 51.9 | 6.8 | 69.2 | 130.8 | 1.9 |
| Delta(14)‐sterol reductase LBR OS=Rattus n | o O08984 | 5.48 | 2 | 620 | 70.7 | 9.57 | 69.3 | 130.7 | 1.9 |
| ATP synthase subunit gamma, mitochondria | P35435 | 22.71 | 6 | 273 | 30.2 | 8.84 | 69.4 | 130.6 | 1.9 |
| ATP synthase subunit O, mitochondrial OS= | R Q06647 | 15.49 | 2 | 213 | 23.4 | 10.02 | 69.4 | 130.6 | 1.9 |
| tRNA methyltransferase 10 homolog C OS=R | Q5U2R4 | 7.73 | 3 | 414 | 48.3 | 9.44 | 69.4 | 130.6 | 1.9 |
| Transforming protein RhoA OS=Rattus norv | e P61589 | 13.47 | 2 | 193 | 21.8 | 6.1 | 69.5 | 130.5 | 1.9 |
| Protein transport protein Sec61 subunit alp | h P61621 | 1.89 | 1 | 476 | 52.2 | 8.06 | 69.5 | 130.5 | 1.9 |
| 40S ribosomal protein SA OS=Rattus norveg | P38983 | 43.39 | 9 | 295 | 32.8 | 4.87 | 69.6 | 130.4 | 1.9 |
| Synaptopodin 2‐like OS=Rattus norvegicus O | D3ZZ68 | 14.93 | 9 | 978 | 103 | 9.73 | 69.6 | 130.4 | 1.9 |
| Proton‐translocating NAD(P)(+) transhydrog | Q5BJZ3 | 13.44 | 10 | 1086 | 113.8 | 7.88 | 69.7 | 130.3 | 1.9 |
| 40S ribosomal protein S9 OS=Rattus norveg | P29314 | 20.62 | 7 | 194 | 22.6 | 10.65 | 69.7 | 130.3 | 1.9 |
| Actinin alpha 2 OS=Rattus norvegicus OX=1 | 0 D3ZCV0 | 61.41 | 34 | 894 | 103.8 | 5.45 | 69.9 | 130.1 | 1.9 |

| 40S ribosomal protein S3a OS=Rattus norve | g P49242 | 46.59 | 13 | 264 | 29.9 | 9.73 | 69.9 | 130.1 | 1.9 |
| --- | --- | --- | --- | --- | --- | --- | --- | --- | --- |
| Long‐chain specific acyl‐CoA dehydrogenase | P15650 | 16.74 | 6 | 430 | 47.8 | 7.74 | 69.9 | 130.1 | 1.9 |
| Serine/threonine‐protein phosphatase 2A c | a P62716 | 6.15 | 2 | 309 | 35.6 | 5.43 | 69.9 | 130.1 | 1.9 |
| H1.2 linker histone, cluster member OS=Rat | A0A0G2K654 | 35.85 | 3 | 212 | 21.3 | 11 | 70 | 130 | 1.9 |
| Muscular LMNA‐interacting protein (Fragm | eA0A096MJS3 | 17.05 | 9 | 774 | 82.2 | 8.88 | 70 | 130 | 1.9 |
| Cysteine and glycine‐rich protein 3 OS=Ratt | u G3V7U0 | 26.80 | 3 | 194 | 20.8 | 8.54 | 70.2 | 129.8 | 1.8 |
| Ig‐like domain‐containing protein OS=Rattu | s M0RAZ1 | 10.78 | 1 | 102 | 11.1 | 5.96 | 70.2 | 129.8 | 1.8 |
| Actin, beta‐like 2 OS=Rattus norvegicus OX= | D3ZRN3 | 22.07 | 2 | 376 | 41.9 | 5.49 | 70.4 | 129.6 | 1.8 |
| Actin‐like protein 3 OS=Rattus norvegicus O | A0A0G2K1C0 | 32.46 | 10 | 419 | 47.6 | 6.18 | 70.4 | 129.6 | 1.8 |
| 60S ribosomal protein L24 OS=Rattus norve | g P83732 | 32.48 | 5 | 157 | 17.8 | 11.25 | 70.4 | 129.6 | 1.8 |
| Sorbin and SH3 domain‐containing protein | 1 F1M840 | 5.52 | 2 | 724 | 81.3 | 6.98 | 70.4 | 129.6 | 1.8 |
| MICOS complex subunit MIC60 OS=Rattus n | A0A0G2JVH4 | 27.19 | 16 | 776 | 86.2 | 5.85 | 70.5 | 129.5 | 1.8 |
| Endoplasmin OS=Rattus norvegicus OX=101 | Q66HD0 | 11.82 | 6 | 804 | 92.7 | 4.81 | 70.5 | 129.5 | 1.8 |
| 60S ribosomal protein L38 OS=Rattus norve | g D4AD70 | 50.00 | 4 | 70 | 8.2 | 10.1 | 70.5 | 129.5 | 1.8 |
| Myozenin 2 OS=Rattus norvegicus OX=1011 | A0A0G2KAQ5 | 20.83 | 4 | 264 | 29.8 | 7.55 | 70.5 | 129.5 | 1.8 |
| Elongation factor 1‐delta OS=Rattus norvegi | Q68FR9 | 8.54 | 2 | 281 | 31.3 | 5.06 | 70.5 | 129.5 | 1.8 |
| Histone H1.4 OS=Rattus norvegicus OX=101 | P15865 | 37.90 | 2 | 219 | 22 | 11.11 | 70.6 | 129.4 | 1.8 |
| Fascin OS=Rattus norvegicus OX=10116 GN | =A0A0G2KAM4 | 5.28 | 2 | 492 | 53.8 | 7.4 | 70.6 | 129.4 | 1.8 |
| Adenosylhomocysteinase OS=Rattus norveg | P10760 | 2.78 | 1 | 432 | 47.5 | 6.54 | 70.6 | 129.4 | 1.8 |
| Nucleolar protein 6 OS=Rattus norvegicus O | A0A096MKG2 | 2.01 | 2 | 1143 | 127.7 | 7.25 | 70.7 | 129.3 | 1.8 |
| Myosin, heavy polypeptide 9, non‐muscle O | G3V6P7 | 70.56 | 12 | 1960 | 226.3 | 5.66 | 70.8 | 129.2 | 1.8 |
| Histone H1.5 OS=Rattus norvegicus OX=101 | D3ZBN0 | 30.18 | 6 | 222 | 22.6 | 10.96 | 70.8 | 129.2 | 1.8 |
| Elongation factor G, mitochondrial OS=Ratt | u F1LMM9 | 1.20 | 1 | 749 | 83.4 | 7.03 | 70.8 | 129.2 | 1.8 |
| Myosin XVIIIb OS=Rattus norvegicus OX=10 | 1 M0R6Z9 | 3.76 | 8 | 2551 | 284.1 | 6.48 | 70.9 | 129.1 | 1.8 |

| Elongation factor 2 OS=Rattus norvegicus O | X | P05197 | 11.42 | 8 | 858 | 95.2 | 6.83 | 71.1 | 128.9 | 1.8 |
| --- | --- | --- | --- | --- | --- | --- | --- | --- | --- | --- |
| 60S ribosomal protein L4 OS=Rattus norvegi |  | Q6P3V9 | 4.99 | 2 | 421 | 47.3 | 11.02 | 71.2 | 128.8 | 1.8 |
| DEAD box protein 5 OS=Rattus norvegicus O |  | Q6AYI1 | 15.45 | 5 | 615 | 69.2 | 8.92 | 71.4 | 128.6 | 1.8 |
| Endoplasmic reticulum chaperone BiP OS=R |  | P06761 | 26.30 | 11 | 654 | 72.3 | 5.16 | 71.5 | 128.5 | 1.8 |
| 40S ribosomal protein S8 OS=Rattus norveg |  | P62243 | 52.88 | 10 | 208 | 24.2 | 10.32 | 71.6 | 128.4 | 1.8 |
| Putative oxidoreductase GLYR1 OS=Rattus n |  | Q5RKH0 | 7.07 | 3 | 552 | 60.4 | 9.13 | 71.6 | 128.4 | 1.8 |
| Cytochrome c‐1 OS=Rattus norvegicus OX=1 |  | D3ZFQ8 | 11.04 | 2 | 326 | 35.4 | 9.26 | 71.6 | 128.4 | 1.8 |
| Thioredoxin OS=Rattus norvegicus OX=1011 |  | P11232 | 12.38 | 1 | 105 | 11.7 | 4.92 | 71.7 | 128.3 | 1.8 |
| RNA helicase OS=Rattus norvegicus OX=101 |  | Q6P3V8 | 15.02 | 5 | 406 | 46.1 | 5.48 | 71.8 | 128.2 | 1.8 |
| Embryonal Fyn‐associated substrate OS=Rat |  | B1WBZ1 | 8.91 | 3 | 561 | 59 | 5 | 71.8 | 128.2 | 1.8 |
| Insulin‐like growth factor 2 receptor OS=Rat |  | G3V824 | 0.93 | 2 | 2481 | 273.4 | 5.71 | 71.8 | 128.2 | 1.8 |
| Complement C1q subcomponent subunit C |  | P31722 | 3.67 | 1 | 245 | 25.7 | 8.32 | 71.8 | 128.2 | 1.8 |
| Citrate synthase, mitochondrial OS=Rattus n |  | Q8VHF5 | 16.31 | 6 | 466 | 51.8 | 8.35 | 71.9 | 128.1 | 1.8 |
| Actin‐related protein 2/3 complex subunit 4 |  | B2RZ72 | 16.07 | 3 | 168 | 19.7 | 8.43 | 71.9 | 128.1 | 1.8 |
| Vanin 1 OS=Rattus norvegicus OX=10116 GN |  | Q4KLZ0 | 5.08 | 2 | 512 | 57 | 5.55 | 71.9 | 128.1 | 1.8 |
| 40S ribosomal protein S23 OS=Rattus norve |  | P62268 | 48.95 | 5 | 143 | 15.8 | 10.49 | 72 | 128 | 1.8 |
| Thyroid hormone receptor‐associated prote |  | Q5M7V8 | 3.15 | 2 | 951 | 108.2 | 10.15 | 72 | 128 | 1.8 |
| Poly(rC)‐binding protein 2 OS=Rattus norve | g | Q6AYU5 | 8.49 | 2 | 365 | 38.6 | 6.79 | 72.1 | 127.9 | 1.8 |
| Galectin‐1 OS=Rattus norvegicus OX=10116 |  | P11762 | 20.74 | 2 | 135 | 14.8 | 5.26 | 72.1 | 127.9 | 1.8 |
| Non‐muscle caldesmon OS=Rattus norvegic |  | Q62736 | 4.71 | 2 | 531 | 60.5 | 6.58 | 72.1 | 127.9 | 1.8 |
| Immunoglobulin heavy constant mu OS=Rat |  | F1LM30 | 6.46 | 3 | 480 | 52.9 | 6.25 | 72.2 | 127.8 | 1.8 |
| Cysteine‐rich protein 2 OS=Rattus norvegicu |  | P36201 | 18.27 | 2 | 208 | 22.7 | 8.63 | 72.4 | 127.6 | 1.8 |
| Myl9 protein OS=Rattus norvegicus OX=101 |  | B0BMS8 | 62.21 | 5 | 172 | 19.8 | 4.92 | 72.5 | 127.5 | 1.8 |
| Histidine‐‐tRNA ligase OS=Rattus norvegicus |  | F1M9C9 | 6.11 | 3 | 507 | 57.3 | 7.85 | 72.5 | 127.5 | 1.8 |

| Proteasome subunit alpha type‐3 OS=Rattu | s P18422 | 4.71 | 1 | 255 | 28.4 | 5.44 | 72.5 | 127.5 | 1.8 |
| --- | --- | --- | --- | --- | --- | --- | --- | --- | --- |
| Cytoskeleton‐associated protein 4 OS=Rattu | D3ZH41 | 11.66 | 3 | 326 | 36.3 | 4.82 | 72.6 | 127.4 | 1.8 |
| 40S ribosomal protein S25 OS=Rattus norve | A0A0H2UHU0 | 27.42 | 5 | 124 | 13.6 | 10.11 | 72.8 | 127.2 | 1.7 |
| Ig‐like domain‐containing protein OS=Rattu | s F1M5X4 | 12.10 | 1 | 124 | 13.7 | 5.41 | 72.8 | 127.2 | 1.7 |
| 60S ribosomal protein L11 OS=Rattus norve | g P62914 | 41.57 | 6 | 178 | 20.2 | 9.6 | 72.9 | 127.1 | 1.7 |
| 40S ribosomal protein S15a OS=Rattus norv | D3ZLL8 | 32.31 | 4 | 130 | 14.8 | 10.13 | 72.9 | 127.1 | 1.7 |
| Peptidyl‐prolyl cis‐trans isomerase FKBP1A | O Q62658 | 25.93 | 2 | 108 | 11.9 | 8.16 | 72.9 | 127.1 | 1.7 |
| Xin actin‐binding repeat‐containing 1 OS=Ra | D4ABA9 | 36.69 | 32 | 1153 | 125.7 | 5.41 | 73 | 127 | 1.7 |
| Moesin OS=Rattus norvegicus OX=10116 GN | A0A096MK30 | 10.40 | 6 | 577 | 67.8 | 6.47 | 73 | 127 | 1.7 |
| Delta(3,5)‐Delta(2,4)‐dienoyl‐CoA isomerase | Q62651 | 7.95 | 2 | 327 | 36.1 | 7.99 | 73 | 127 | 1.7 |
| Proteasome subunit beta OS=Rattus norveg | G3V8U9 | 10.78 | 2 | 232 | 25.7 | 6.34 | 73 | 127 | 1.7 |
| 40S ribosomal protein S11 OS=Rattus norve | P62282 | 34.81 | 6 | 158 | 18.4 | 10.3 | 73.1 | 126.9 | 1.7 |
| CCN family member 2 OS=Rattus norvegicus | Q9R1E9 | 17.58 | 6 | 347 | 37.7 | 7.87 | 73.1 | 126.9 | 1.7 |
| Myosin‐7 OS=Rattus norvegicus OX=10116 | G G3V8B0 | 63.10 | 45 | 1935 | 222.8 | 5.76 | 73.3 | 126.7 | 1.7 |
| Proteasome subunit alpha type‐4 OS=Rattu | s P21670 | 6.51 | 2 | 261 | 29.5 | 7.72 | 73.4 | 126.6 | 1.7 |
| 40S ribosomal protein S4 OS=Rattus norveg | D3ZX01 | 27.48 | 1 | 262 | 29.2 | 10.27 | 73.5 | 126.5 | 1.7 |
| Four and a half LIM domains protein 1 OS=R | A0A0G2K338 | 21.83 | 6 | 339 | 37.9 | 9.09 | 73.5 | 126.5 | 1.7 |
| Actin‐related protein 2/3 complex subunit 2 | P85970 | 12.00 | 4 | 300 | 34.4 | 7.36 | 73.6 | 126.4 | 1.7 |
| Uncharacterized protein (Fragment) OS=Rat | F1LWK7 | 10.33 | 3 | 600 | 67 | 8.51 | 73.8 | 126.2 | 1.7 |
| H1.3 linker histone, cluster member OS=Rat | M0R7B4 | 30.00 | 1 | 220 | 22.2 | 11.11 | 74 | 126 | 1.7 |
| 60S ribosomal protein L10 OS=Rattus norve | g Q6PDV7 | 29.44 | 8 | 214 | 24.6 | 10.08 | 74 | 126 | 1.7 |
| Ras‐related protein Rab‐7a OS=Rattus norve | P09527 | 9.66 | 2 | 207 | 23.5 | 6.7 | 74.2 | 125.8 | 1.7 |
| Keratin 86 OS=Rattus norvegicus OX=10116 | A0A0G2QC11 | 15.78 | 8 | 488 | 53.4 | 5.87 | 74.3 | 125.7 | 1.7 |
| Arginine and glutamate‐rich protein 1 OS=R | Q5BJT0 | 5.90 | 2 | 271 | 32.9 | 10.36 | 74.4 | 125.6 | 1.7 |

| IKAROS family zinc finger 4 OS=Rattus norve | D4A636 | 7.34 | 3 | 586 | 64.1 | 6.86 | 74.5 | 125.5 | 1.7 |
| --- | --- | --- | --- | --- | --- | --- | --- | --- | --- |
| Zinc finger protein 771 OS=Rattus norvegicu | F1M1R7 | 12.62 | 3 | 317 | 35.7 | 8.82 | 74.5 | 125.5 | 1.7 |
| Troponin I, slow skeletal muscle OS=Rattus | nA0A096MIZ5 | 41.71 | 11 | 187 | 21.7 | 9.58 | 74.7 | 125.3 | 1.7 |
| 60S acidic ribosomal protein P0 OS=Rattus n | P19945 | 3.15 | 1 | 317 | 34.2 | 6.25 | 74.7 | 125.3 | 1.7 |
| Cleavage and polyadenylation‐specific facto | B5DEL2 | 9.17 | 11 | 1439 | 160.7 | 6.39 | 74.8 | 125.2 | 1.7 |
| Aldehyde dehydrogenase, mitochondrial OS | F1LN88 | 13.49 | 6 | 519 | 56.5 | 7.33 | 74.8 | 125.2 | 1.7 |
| 60S ribosomal protein L29 OS=Rattus norve | g P25886 | 12.18 | 2 | 156 | 17.3 | 11.78 | 74.9 | 125.1 | 1.7 |
| 60S ribosomal protein L13 OS=Rattus norve | g P41123 | 24.64 | 5 | 211 | 24.3 | 11.55 | 75 | 125 | 1.7 |
| Catalase OS=Rattus norvegicus OX=10116 G | P04762 | 7.40 | 3 | 527 | 59.7 | 7.5 | 75 | 125 | 1.7 |
| Voltage‐dependent anion‐selective channel | A0A0G2JSR0 | 7.07 | 1 | 283 | 30.8 | 8.59 | 75.1 | 124.9 | 1.7 |
| Malate dehydrogenase, mitochondrial OS=R | P04636 | 27.51 | 8 | 338 | 35.7 | 8.68 | 75.3 | 124.7 | 1.7 |
| Ig‐like domain‐containing protein OS=Rattu | s F1M3H2 | 9.09 | 1 | 99 | 11.2 | 6.61 | 75.4 | 124.6 | 1.7 |
| Tubulin beta chain OS=Rattus norvegicus OX | G3V7C6 | 47.99 | 3 | 548 | 61.1 | 4.86 | 75.5 | 124.5 | 1.6 |
| Coiled‐coil‐helix‐coiled‐coil‐helix domain co | D3ZUX5 | 15.42 | 3 | 227 | 26.4 | 8.13 | 75.5 | 124.5 | 1.6 |
| 60S ribosomal protein L28 OS=Rattus norve | g D3ZJD3 | 24.82 | 4 | 137 | 15.6 | 11.93 | 75.8 | 124.2 | 1.6 |
| Ig‐like domain‐containing protein OS=Rattu | s D3ZQR5 | 10.34 | 1 | 116 | 12.8 | 7.12 | 75.8 | 124.2 | 1.6 |
| 60S ribosomal protein L27a OS=Rattus norv | eA0A0G2K7W6 | 31.08 | 4 | 148 | 16.6 | 10.99 | 75.9 | 124.1 | 1.6 |
| Synaptopodin‐2 OS=Rattus norvegicus OX=1 | D4A702 | 10.38 | 8 | 1262 | 135.9 | 8.51 | 76 | 124 | 1.6 |
| 2‐phospho‐D‐glycerate hydro‐lyase OS=Ratt | M0R5J4 | 27.65 | 7 | 434 | 47 | 6.37 | 76.3 | 123.7 | 1.6 |
| LLGL scribble cell polarity complex compone | A0A0G2JU05 | 3.34 | 3 | 1077 | 119.6 | 8.19 | 76.3 | 123.7 | 1.6 |
| Ubiquitin‐40S ribosomal protein S27a OS=R | a P62982 | 51.28 | 7 | 156 | 17.9 | 9.64 | 76.5 | 123.5 | 1.6 |
| BAG cochaperone 2 OS=Rattus norvegicus O | F1LQH9 | 4.72 | 1 | 212 | 23.8 | 6.09 | 76.5 | 123.5 | 1.6 |
| ADP/ATP translocase OS=Rattus norvegicus | Q6P9Y4 | 55.37 | 9 | 298 | 32.9 | 9.74 | 76.6 | 123.4 | 1.6 |
| HP domain‐containing protein OS=Rattus no | A0A0G2JW01 | 34.19 | 9 | 427 | 48.6 | 8.07 | 76.6 | 123.4 | 1.6 |

| Polyadenylate‐binding protein 1 OS=Rattus | Q9EPH8 | 4.09 | 2 | 636 | 70.7 | 9.5 | 76.8 | 123.2 | 1.6 |
| --- | --- | --- | --- | --- | --- | --- | --- | --- | --- |
| CWF19‐like cell cycle control factor 2 OS=Ra | D4A877 | 1.13 | 1 | 888 | 102.9 | 8.37 | 76.8 | 123.2 | 1.6 |
| Proteasome subunit beta type‐2 OS=Rattus | P40307 | 7.46 | 1 | 201 | 22.9 | 7.44 | 76.9 | 123.1 | 1.6 |
| F‐actin‐capping protein subunit alpha‐2 OS= | Q3T1K5 | 70.63 | 12 | 286 | 32.9 | 5.85 | 77.1 | 122.9 | 1.6 |
| Fatty acid‐binding protein, heart OS=Rattus | P07483 | 27.82 | 3 | 133 | 14.8 | 6.33 | 77.1 | 122.9 | 1.6 |
| Ig gamma‐2B chain C region OS=Rattus norv | P20761 | 4.80 | 1 | 333 | 36.5 | 7.64 | 77.3 | 122.7 | 1.6 |
| RuvB‐like 1 OS=Rattus norvegicus OX=1011 | 6 P60123 | 2.85 | 1 | 456 | 50.2 | 6.42 | 77.5 | 122.5 | 1.6 |
| GMP reductase 1 OS=Rattus norvegicus OX= | Q9Z244 | 6.38 | 2 | 345 | 37.5 | 6.98 | 77.5 | 122.5 | 1.6 |
| Band 4.1‐like protein 5 OS=Rattus norvegicu | D3ZK45 | 2.78 | 1 | 504 | 57.6 | 9.29 | 77.6 | 122.4 | 1.6 |
| Elongation factor 1‐alpha OS=Rattus norveg | M0R757 | 33.12 | 5 | 462 | 50.1 | 9.01 | 77.7 | 122.3 | 1.6 |
| Unconventional myosin‐XVIIIa OS=Rattus no | A0A0G2JY08 | 35.74 | 59 | 2054 | 233.2 | 6.24 | 77.8 | 122.2 | 1.6 |
| Alpha‐actinin‐1 OS=Rattus norvegicus OX=1 | Q6T487 | 32.82 | 8 | 914 | 105.4 | 5.39 | 77.9 | 122.1 | 1.6 |
| 40S ribosomal protein S21 OS=Rattus norve | D3ZSE0 | 24.10 | 3 | 83 | 9.1 | 8.51 | 77.9 | 122.1 | 1.6 |
| Heat shock protein beta‐1 OS=Rattus norve | g P42930 | 28.64 | 5 | 206 | 22.9 | 6.55 | 78 | 122 | 1.6 |
| Cytochrome c oxidase subunit 4 isoform 1, | m P10888 | 7.10 | 1 | 169 | 19.5 | 9.44 | 78.1 | 121.9 | 1.6 |
| Complement component 1 Q subcomponen | O35796 | 5.02 | 1 | 279 | 31 | 4.87 | 78.1 | 121.9 | 1.6 |
| Cleavage and polyadenylation specificity fac | G3V6W7 | 5.99 | 3 | 685 | 77.6 | 5.6 | 78.2 | 121.8 | 1.6 |
| Myosin light chain 3 OS=Rattus norvegicus | O P16409 | 73.50 | 14 | 200 | 22.1 | 5.1 | 78.3 | 121.7 | 1.6 |
| Ig‐like domain‐containing protein OS=Rattu | s M0RDL2 | 12.75 | 1 | 102 | 11.3 | 7.39 | 78.3 | 121.7 | 1.6 |
| Tubulin beta‐5 chain OS=Rattus norvegicus | O P69897 | 56.98 | 3 | 444 | 49.6 | 4.89 | 78.8 | 121.2 | 1.5 |
| 40S ribosomal protein S13 OS=Rattus norve | P62278 | 32.45 | 5 | 151 | 17.2 | 10.54 | 78.9 | 121.1 | 1.5 |
| LRRGT00052 OS=Rattus norvegicus OX=101 | Q6TXE7 | 11.93 | 22 | 2531 | 278 | 8 | 79.1 | 120.9 | 1.5 |
| 40S ribosomal protein S24 OS=Rattus norve | A0A0H2UHH9 | 20.61 | 2 | 131 | 15.2 | 10.89 | 79.3 | 120.7 | 1.5 |
| Myosin heavy chain 14 OS=Rattus norvegicu | F1LNF0 | 50.65 | 88 | 2000 | 228.8 | 5.55 | 79.4 | 120.6 | 1.5 |

| Actin, cytoplasmic 1 OS=Rattus norvegicus O | P60711 | 92.27 | 15 | 375 | 41.7 | 5.48 | 79.4 | 120.6 | 1.5 |
| --- | --- | --- | --- | --- | --- | --- | --- | --- | --- |
| ADP/ATP translocase 2 OS=Rattus norvegicu | Q09073 | 47.65 | 6 | 298 | 32.9 | 9.73 | 79.7 | 120.3 | 1.5 |
